# Counting days is a spacing incentive that unlocks the potential of low GPA students

**DOI:** 10.1038/s41539-025-00322-5

**Published:** 2025-06-05

**Authors:** Iman YeckehZaare, Paul Resnick

**Affiliations:** 1 MIT Center for Collective Intelligence, Sloan School of Management, Massachusetts Institute of Technology, Cambridge, MA USA; 2 Research, Honor Education, San Francisco, MA USA; 3 School of Information, University of Michigan, Ann Arbor, MI USA

**Keywords:** Education, Human behaviour

## Abstract

Spacing and retrieval practice enhance learning, but students often underuse these strategies. We tested a simple grading incentive, which we call Counting Days, in two RCTs: one randomizing 143 students within a course and another randomizing 71 instructors. The “counting questions” control condition awarded points for each practice question answered, while the “counting days” treatment assignment awarded points for each day that a student answered a set of questions. In the within-class experiment, the counting days group earned higher exam scores, mediated by spacing practice over more days. Spacing was especially beneficial for lower-GPA students: the correlation between course exam scores and GPA in prior courses was significantly lower for the counting days group. In the between-instructor experiment, there was no way to compare learning outcomes between instructors, but both the number of days and a number of questions practiced were significantly higher under the counting days condition.

## Introduction

The science of learning and memory has identified a set of “desirable difficulties,” so-called because while they contribute to learning, students find them challenging and may not recognize their value^1–5^. Zepeda et al.^4^ argued a need for mechanisms to motivate students “to embrace or, at minimum cope, with [these] difficulties.”

Among these desirable difficulties, spacing is particularly noteworthy for its robust and reliable effect on long-term learning. Massing is better for short-term performance but spacing is better for long-term learning^6–8^. The spacing effect is “one of the most robust and reliable effects in all of memory research”^8–10^. The “new theory of disuse” explains it as long-term learning resulting from forgetting between study sessions^5,11^. Studies demonstrating the substantial benefits of spacing on knowledge retention and inductive learning^8,12–15^ have included participants from 3-year-old children learning fundamental concepts and categories^13,16^ to 60-year-old adults acquiring new knowledge and skills^13,14,17^.

Previous experiments that have demonstrated the positive effects of spaced practice have done so by forcing students to space. For example, classroom time is devoted to it^6,18–21^ or subjects are brought in to a lab (in-person or remotely) to perform assigned tasks^7,12–17,22,23^. Unfortunately, when students have a choice, spacing tends to be underutilized^2,24–27^. Left to their own devices, students cram for exams rather than studying a little each day^3,27–30^.

One reason for underutlization of spacing is that students incorrectly perceive that spacing leads to lower performance^1–4,30–32^. Massing, by contrast, creates an “illusion of mastery”^1,5^: it helps with short-term performance without generating long-term retention. Students who were asked about their judgment of learning reported massing as more effective than spacing, even after experiencing the effectiveness of spacing^1,27,32^. Students also find spacing challenging and they tend to think that more difficulty means less effective learning, which negatively affects their willingness to use spacing^1,2,32,33^.

Another reason students underutilize spacing is that it requires a high degree of self-regulation. Many people are convinced that frequent exercise will improve their long-term health, but it is difficult to maintain an exercise regimen^34^. Similarly, even when students set an intention to study a little on a lot of days, they rarely maintain the habit^3,30^.

Merely informing students about the benefits of spacing or encouraging them to do so is not sufficient to alter student behavior^21,35,36^. Even students who reported that they were aware of the benefits of spacing reported that they did not regularly do so^27^. One potential counterpoint comes from Li et al.^37^, where a section of 100 students who received more frequent reminders to use a practice tool used it much more and received higher grades.

Insufficient spacing may be especially problematic for low-performing students. A large-scale observational study^38^ on a psychology MOOC found that low-ability students, and those who are more likely to leave assignments incomplete, benefit more from spacing. Hartwig and Dunlosky^28^ explained that students with lower GPAs are more likely to make their study decisions based on deadlines and engage in cramming. Finn^39^ identified differences in study habits as one factor that explains differences in student performance. Thus, interventions that lead students to successfully engage in spacing may help lower-GPA students to achieve up to their potential.

Given the potential value of altering students’ study practices to increase spacing, there is an open question of how to effectively incentivize it. Incentives for frequent activity have been implemented in video games^40^, learning^41,42^, and fitness^43^. The most common form is a gamification of “streaks,” where missing a day of activity breaks the sequence, restarting the counter at zero. This can motivate people to extend streaks, especially once they are long. For example, on Duolingo, learners with longer streaks were more likely to extend their streaks for another day^41^. Another form counts days of activity without requiring an uninterrupted streak. For example, one exercise app encouraged participants to set a goal for the number of days in a week that they would exceed a step-count threshold^43^.

Following the latter idea, we designed a grade-based incentive that rewards the number of active days. A student earns course points for each day they correctly answer a set of practice questions. We refer to this incentive mechanism as “counting days.”

The rationale behind this incentive design is that it only rewards spaced activity. An equivalent amount of total activity massed on the few days just before the exams would earn fewer course points. Spacing is more beneficial with sessions separated by 24-hour intervals rather than shorter ones such as 30 minutes^44^. One reason may be that sleep cycles between sessions allow for memory consolidation, which further enhances learning^45^. Thus, our incentive provides points per day rather than points per practice session.

We implemented the counting days incentive in a retrieval practice tool. Retrieval practice happens when previously learned information is retrieved from memory, typically by answering a question. It has been found to improve long-term learning^46–48^ and protect memory against acute stress^49^. Retrieval practice is especially helpful when spaced rather than massed because it allows for the forgetting and relearning process, which strengthens memory traces and makes the information more durable^5,8,10,50^.

We implemented two distinct incentive conditions. In the intervention condition, “Counting Days,” students earn a fixed increment of points for every day on which they correctly answer at least ten practice questions, thereby promoting spaced learning. In contrast, in the control condition, “Counting Questions,” points are awarded on a per-question basis without any requirement to distribute practice over multiple days.

In a preliminary study, the Counting Days incentive was given to all students for one semester, which allowed for refinement of the tool. In that semester, more practice was correlated with higher exam performance^51^.

Here, we report on two randomized controlled experiments, where some students or entire classes were assigned to the Counting Days condition and others to the Counting Questions condition. In Experiment 1, conducted in Fall 2018 at a large public university, 143 students in an introductory programming course were randomized at the individual level to either the Counting Days or Counting Questions condition. In Experiment 2, 71 instructors from multiple high schools and colleges taught over 1600 students, with instructors randomly assigned to one of the two conditions (ensuring no student participated in both experiments). Practice behavior was measured in both experiments. For Experiment 1, students’ prior GPA and exam performance in the course were also measured. Our hypotheses are that incentivizing practice distributed across multiple days (Counting Days) would foster more effective spacing of study sessions, leading to higher final exam scores, and that the benefits of the Counting Days condition would be greater for students with lower prior GPAs. For the full methodological details, please refer to the Methods section at the end.

## Results

### Experiment 1: Within-class

In the within-class experiment, final exam scores were the primary outcome measure, and the number of days and total number of questions practiced were intermediate behavioral outcomes. Table 1 presents summary statistics of these outcome variables for the within-class experiment, with each variable displayed in three rows. The mean final exam score in the counting days treatment was 85.3% vs. 81.7% in the counting questions condition. The number of days practiced was much higher (mean 38.5 vs. 17.3), and the total number of questions answered was a little higher (mean 415.0 vs. 392.5). Table 2 confirms that these differences are statistically significant, according to both parametric t-tests and non-parametric Wilcoxon rank-sum (Mann-Whitney) tests.

**Table 1:** Summary statistics for the within-class experiment

	Group	Min	Median	Max	Mean	SD
**Final Exam Score (in %)**	Counting Days	35.6	87.3	100	85.3	10.0
	Counting Questions	49.1	83.3	100	81.7	11.6
**Practice Days**	Counting Days	16	39	48	38.5	5.0
	Counting Questions	4	17	40	17.3	7.4
**Practiced Questions**	Counting Days	190	412	662	415.0	61.7
	Counting Questions	170	401	444	392.5	46.3

Descriptive statistics, by condition, for each of the three outcome variables.

**Table 2:** Significance tests for the within-class experiment

	Two-sample t-test with equal variances	Two-sample Wilcoxon rank-sum (Mann-Whitney) test
**Final Exam Score**	1.988*	1.968*
**Practiced Days**	20.436***	9.767***
**Practiced Questions**	2.380**	2.745**

Significance levels are indicated as *** *p* < 0.001; ** *p* < 0.01; * *p* < 0.05.

To assess whether the counting days treatment affected exam scores differently for low- and high-GPA students, we estimated a Beta regression model with an interaction term between the treatment and GPA. We also estimated negative binomial regressions for the count outcomes of the number of days practiced and the number of questions practiced. The regression models include control variables for gender, ethnicity, academic level, and native language (English or not). Table 3 presents the results.

**Table 3:** Regression results for the within-class experiment

	Final Exam Score (Beta)	Days Practiced (Neg. Bin.)	Questions Practiced (Neg. Bin.)
**Counting Days** **(vs. Questions)**	2.215** (0.811)	1.638*** (0.305)	0.216 (0.186)
**GPA**	0.784*** (0.155)	0.223** (0.072)	0.093* (0.037)
**Counting Days** × **GPA**	−0.605* (0.235)	−0.247** (0.088)	−0.049 (0.054)
**Female** **(vs. Male)**	−0.139 (0.121)	0.133*** (0.040)	0.018 (0.027)
**Asian** **(vs. White)**	0.099 (0.142)	−0.091* (0.044)	−0.041 (0.030)
**Other Ethnicity** **(vs. White)**	− 0.026 (0.145)	−0.066 (0.048)	−0.035 (0.032)
**Junior** **(vs. Sophomore)**	0.312* (0.146)	−0.059 (0.045)	−0.062* (0.031)
**Senior** **(vs. Sophomore)**	0.387** (0.149)	−0.036 (0.046)	0.004 (0.031)
**NonNativeEnglish** **(vs. NativeEnglish)**	0.344* (0.135)	0.011 (0.041)	−0.004 (0.028)
**[Intercept]**	−1.280* (0.522)	2.056*** (0.245)	5.675*** (0.126)
**[Parameter]**	**Scale:** 2.788*** (0.118)	**Alpha:** 0.004*** (0.006)	**Alpha:** 0.016*** (0.002)
**Log Likelihood**	156.444	−470.348	−775.635
**# of Obs**.	143	143	143
*χ*^2^	42.203***	200.502***	23.109**

Column names indicate dependent variables (Final Exam Score, Days Practiced, Questions Practiced). Coefficients represent log odds ratios. Significance levels are denoted as *** *p* < 0.001; ** *p* < 0.01; * *p* < 0.05.

To assess whether the counting days treatment affected exam scores differently for low- and high-GPA students, we estimated a Beta regression model with an interaction term between the treatment and GPA. We also estimated negative binomial regressions for the count outcomes of the number of days practiced and the number of questions practiced. The regression models include control variables for gender, ethnicity, academic level, and native language (English or not). Table 3 presents the results. As detailed in Table 3, the positive effect of the counting days condition on final exam scores and its significant negative interaction with GPA clearly indicate that lower-GPA students benefit more from the Counting Days incentive.

**Fig. 1:**
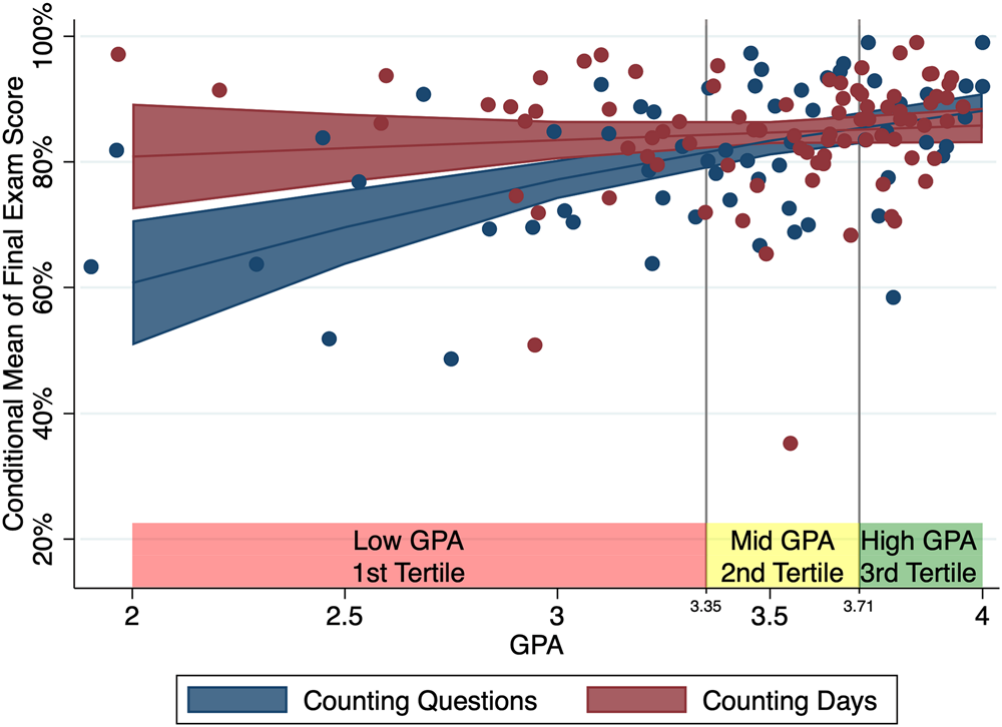
Predicted final exam scores in the within-class experiment. The estimated effect of GPA on final exam scores, based on the Beta regression model predictions of the within-class experiment. Dots represent individual students. Shaded areas represent 95% confidence intervals.

Figure 1 plots the predicted means of final exam scores as a function of GPA, based on the beta regression results. The lines are curved rather than straight due to using a beta regression rather than ordinary linear regression. The red line being above the blue line in the figure comes from the positive coefficient on Counting Days (2.215). The upward slope of the lines comes from the positive coefficient on GPA (0.784). The negative coefficient on the interaction term (−0.605) leads the slope to be much lower in the Counting Days condition: the effect of GPA on exam score is much smaller in this condition.

As a test of the difference in linear correlation between GPA and exam score for a range of GPAs from 2.893 (tenth percentile) to 3.548 (mean), we used the “emtrends” command from the “emmeans” package in R. Under the counting questions condition, the estimated trend is 0.109 (*p* < 0.001). Under the counting days condition, the estimated trend is nearly zero, −0.009 (*p* = 0.704). The contrast of − 0.118 between the two trends is statistically significant (*p* < 0.01).

**Fig. 2:**
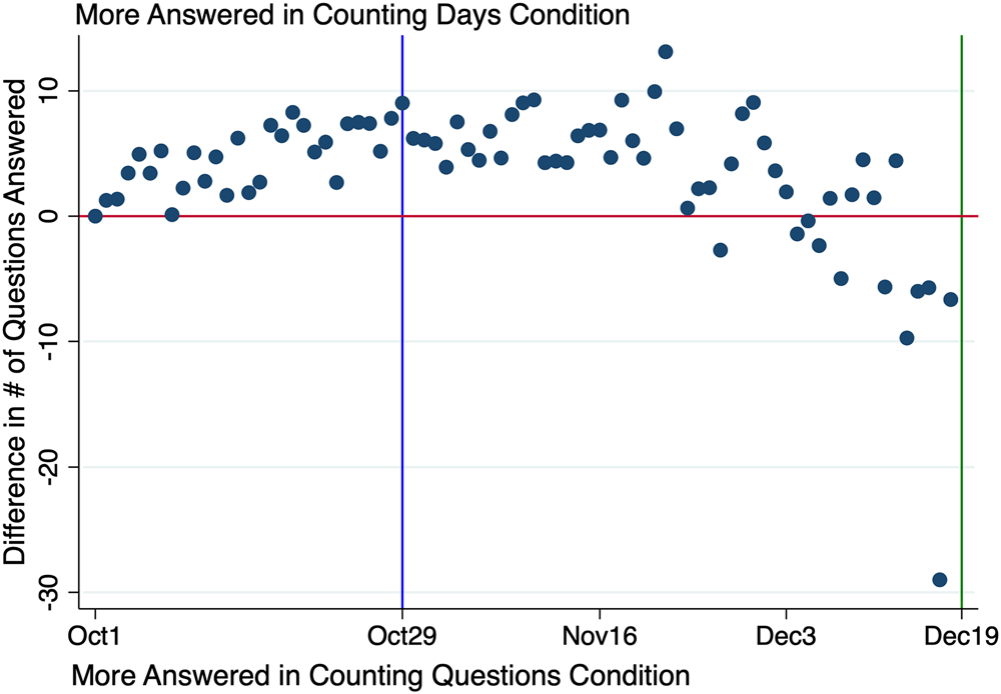
Procrastination patterns in the within-class experiment. Each dot represents one day of activity, calculated as the difference in the average number of questions answered per student. Dots above the red line indicate that the Counting Days group answered more questions on that day. The blue vertical line marks the midterm exam date, and the green line marks the final exam date.

Figure 2 shows that the counting days incentive was effective at influencing student behavior to space out their practice. On most days, students in the counting days condition answered more questions. At the end of the semester, this was reversed indicating that counting questions students procrastinated until late in the semester and then tried to make up for it.

**Fig. 3:**
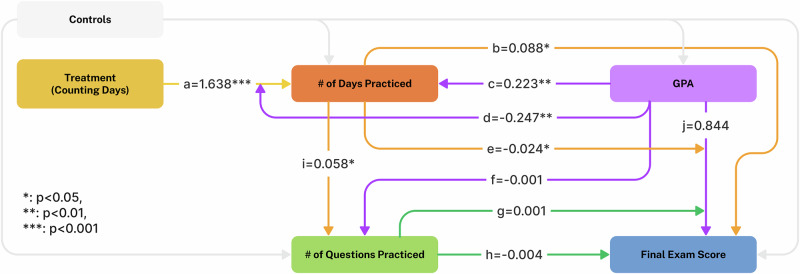
GSEM of mediated effects in the within-class experiment. Boxes represent measured variables. Arrows indicate the modeled direction of effects and coefficients indicate the estimated effect sizes. Interaction terms are shown as arrows pointing to other arrows.

To better understand the mechanism by which the treatment helped low-GPA students, we conducted a mediation analysis using Generalized Structural Equation Modeling (GSEM). We assume that prior GPA, as an indicator of the effectiveness of the student’s normal study habits, affects the amount and spacing of practice and, through other unobserved study behaviors, also affects the final exam score. The full model specifications are included in Supplementary Note 2. The path diagram (with results) is shown in Fig. 3.

We again include controls for gender, ethnicity, academic level, and native language, though they are omitted from the figure. Because of randomization, both controls and GPA are drawn from the same distributions in both conditions. However, the relationship between GPA and exam scores may be influenced by these control variables, making it appropriate to include them in our models to ensure accurate interpretations of the treatment effects.

Figure 3 shows the results of the GSEM analysis. The treatment had a large impact on the number of days practiced (*a*). Students with higher GPA practiced on more days (*c*), but the effect of the treatment on practice days was smaller for those with higher GPA (*d*). More days of practice was positively correlated with the total number of questions practiced (*i*). More days of practice increased exam scores (*b*) and *decreased* the correlation between GPA and exam scores (*e*). We do not observe any significant effect of the number of questions practiced on the final exam score (*h*) or its interaction effect on the correlation of GPA and final exam score (*g*). Overall, the analysis shows that the treatment’s impact on both exam scores and the correlation between prior GPA and exam scores was mediated almost entirely by the number of days practiced.

Note that in the figure, the effects on days of practice are controlling for the number of questions answered. Thus, the positive coefficient *b* = 0.088 implies that, for a pool of students with the same GPA who answered the same total number of questions, spacing out that practice over more days was associated with higher exam scores. Similarly, spacing out practice over more days was associated with a reduction in the correlation of prior GPA with exam scores (*e* = −0.024).

**Fig. 4:**
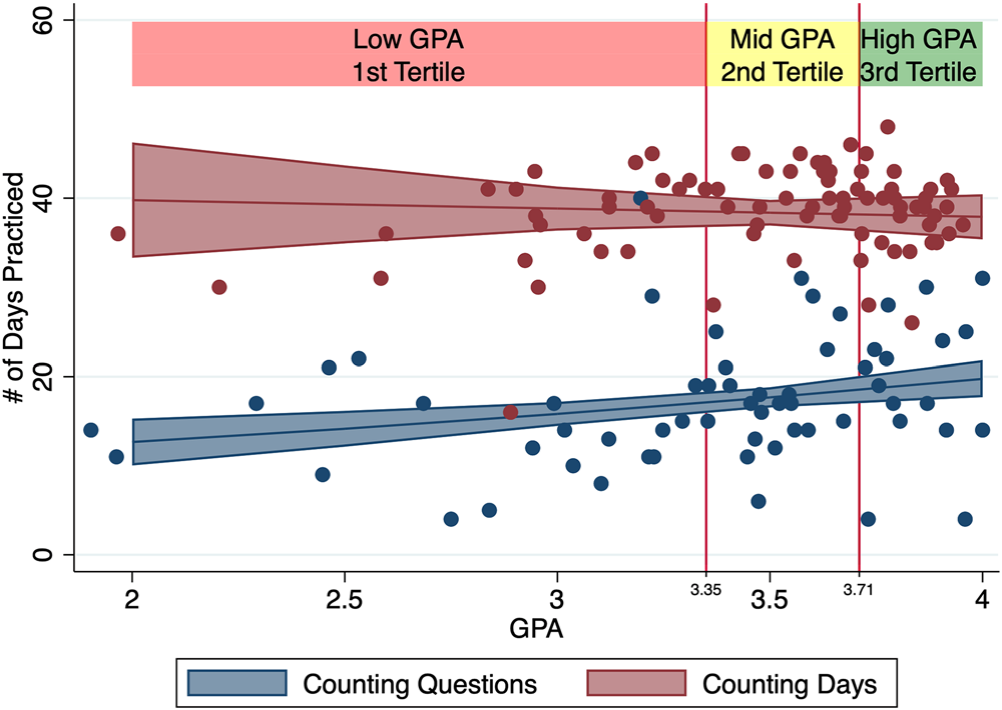
Predicted days of practice in the within-class experiment. The predicted number of days practiced as a function of GPA, based on the GSEM analysis. Dots represent individual students. Shaded regions represent 95% confidence intervals.

To provide intuitions about the mediation, we plot estimated effects based on marginal means post-estimation from the GSEM analysis. Figure 4 shows the effects of GPA and treatment on the number of days practiced. The counting days incentive was highly effective at altering student behavior at all levels of GPA: the number of days practiced more than doubled.

### Experiment 2: Between-Instructors

For the second, between-instructor experiment, we received anonymized data and did not have access to GPAs, exam scores, or control variables. Thus, it was not possible to test the impact of the incentives on exam performance or its differential impact for students with high and low GPAs. The experiment does, however, provide a large-scale check on the impact of the counting days incentive on the behavioral outcomes: number of days and questions practiced.

Students in the counting days condition averaged 17.4 days of practice vs. 12.0 in the counting questions condition, as shown in Table 4. Both a t-test and rank-sum test confirm that the difference was statistically significant, as shown in Table 5. Students in the counting days condition also answered somewhat more total questions. This difference was significant according to the rank-sum test but not the t-test.

**Table 4:** Summary statistics for the between-instructors experiment

Variable	Condition	Min	Median	Mean	Max	SD
# of Days Practiced Per Student	Counting Days	1	7	17.4	254	21.52
	Counting Questions	1	6	12.0	80	14.58
# of Questions Practiced Per Student	Counting Days	1	100	217.0	2,971	277.21
	Counting Questions	1	104	197.4	1,040	209.75

Descriptive statistics, by condition, for each of the two behavioral outcome variables.

**Table 5:** Significance tests for the between-instructor experiment

	Two-sample t-test with equal variances	Two-sample Wilcoxon rank-sum (Mann–Whitney) test
**Practiced Days**	5.616***	9.767***
**Practiced Questions**	1.544	2.745**

Significance levels are denoted as *** *p* < 0.001; ** *p* < 0.01.

## Discussion

Due to the experimentally proven advantages of spacing, multiple studies have argued that instructors should exhort their students to study more often, not just study more^8,52^. Mere exhortation, however, appears not to be enough. In settings where students have the option of cramming, many do so^3,27–30^.

Our first major finding is that a small change in incentives led to a large change in the number of days of practice, as evidenced by both within-class and between-instructor experiments. From the within-class experiment, we find that it had a slightly larger behavioral effect for lower GPA students, but the high GPA students also used the practice tool on many more days in the Counting Days condition.

Our second major finding is a confirmation, in a field experiment, of the value of spacing. In a correlational study, Zhang et al.^42^ found that students who engaged in more study sessions on ungraded material did better on exams, and that, comparing across approximately two-week segments within a course, students did better on end-of-segment assessments in segments in which they engaged in more study sessions on graded material. It did not employ random assignment, making it harder to make causal claims, and did not distinguish between the effect of more total study time (amount of studying) and more study sessions (spacing).

In our within-course experiment, the improved exam scores in the counting days group were not simply due to an increased amount of practice but were primarily the result of increased *spacing* of practice over more days. Suggestive evidence (see Table 1 and Fig. 4) comes from the fact that the counting days group practiced on more than twice as many days on average (38.5 vs. 17.3), but only answered a few more total questions (415 vs. 392.5). More direct evidence for this comes from the GSEM analysis (Fig. 3). The number of days practiced remained a significant predictor of final exam scores, even when controlling for the total number of questions practiced. Moreover, there was no significant additional explanatory power from the number of questions practiced, once the number of days was controlled for. This underscores the importance of spacing as a learning strategy and confirms that the advantages of the spacing effect were indeed captured by our incentive mechanism.

Helping low-GPA students perform up to their potential is an important educational goal that has inspired experimentation with course activities and structures^53^. Our third major finding, from the within-class experiment, is that in the Counting Days condition, exam performance nearly equalized for students with low and high GPAs in prior courses. For the highest GPA students, the spacing incentive was not correlated with higher exam performance, even though it was effective at inducing more spaced practice. This may indicate that higher-GPA students with fewer practice days were already employing other effective study strategies, including spaced use of other materials. In contrast, for the lowest GPA students, the spacing induced by the treatment led to better exam performance.

Jackson initially popularized the term “hidden curriculum” to refer to lessons about the need for compliance and competition in day-to-day interactions in classrooms, a curriculum that students from more privileged backgrounds may have an easier time picking up without explicit instruction^54^. It is now used more broadly, especially in the context of higher education, to include lessons about behaviors that will lead to academic success within the structure of courses, such as turning assignments in on time. In this context, spaced studying is a practice that is analogous to elements of the hidden curriculum; some students have acquired it even before they arrive at college, while others need to be taught or nudged. On the other hand, it is not quite an element of the hidden curriculum, since it contributes to learning rather than merely to getting good grades. Indeed, the Counting Days grading incentive may depend for its effectiveness on students having already internalized the hidden curriculum lesson that they should do whatever activities the instructor assigns points for, because instructors use grading points to signal where they think students should allocate effort to enhance their learning. Thus, it may be more effective as an incentive in more elite institutions. It would be an interesting direction for future research to compare its effectiveness in institutions serving students with different socioeconomic backgrounds.

It seems implausible that the counting days grading incentive, applied to a small portion of the course grade, will always be sufficient to nearly equalize the exam performance of low- and high-GPA students, even if it does differentially help low-GPA students. Supplementary Note 3 reports analyses for three other semesters where all students had the counting days treatment. In all three semesters, the vast majority of students used the practice tool on more than 30 days, suggesting that the incentive was effective at inducing spacing behavior. The correlation between prior GPA and exam scores was negligible in one semester, but not in the other two, where students with higher GPAs in prior courses scored better on exams in the course. Even if it is not a panacea, it appears that the counting days incentive provides scaffolding that helps low-GPA students learn somewhat more effectively.

The counting days incentive to induce spacing is partially aligned with other psychological and behavioral principles. It creates goals that are specific, measurable, and challenging, consistent with goal-setting theory^55^. The goals are achievable, enhancing an individual’s sense of competence, an important element of self-determination theory^56^. It leaves students considerable autonomy to choose the days and times at which to practice, another critical element of self-determination theory. It does not, however, provide complete autonomy, since students do not set their own goals for how much practice to do on how many days. Moreover, the assignment of goals may interfere with students’ commitment to the goals, a key feature of goal-setting theory. Further research could explore ways to provide more autonomy over the goal-setting process while still achieving the desired behavioral outcomes.

One possible limitation of the results is that they may be specific to the particular domain and level of introductory programming. It seems plausible that counting days as a grading incentive would work equally well to incentivize spaced activity of other kinds, as long as that activity was measurable. However, the impact of spaced activity might be specific to the particular kind of activity. It also could depend on using similar question types for assessing learning outcomes as those used in the practice activity.

Counting days is a simple grading incentive that had a large impact in inducing spacing. It is relatively easy to implement for activities that are delivered and tracked through course management systems. We encourage the integration of the counting days approach into courses, either through grading incentives or other creative elements, as part of a campaign to induce students to adopt spaced studying strategies.

## Methods

### Apparatus

**Fig. 5:**
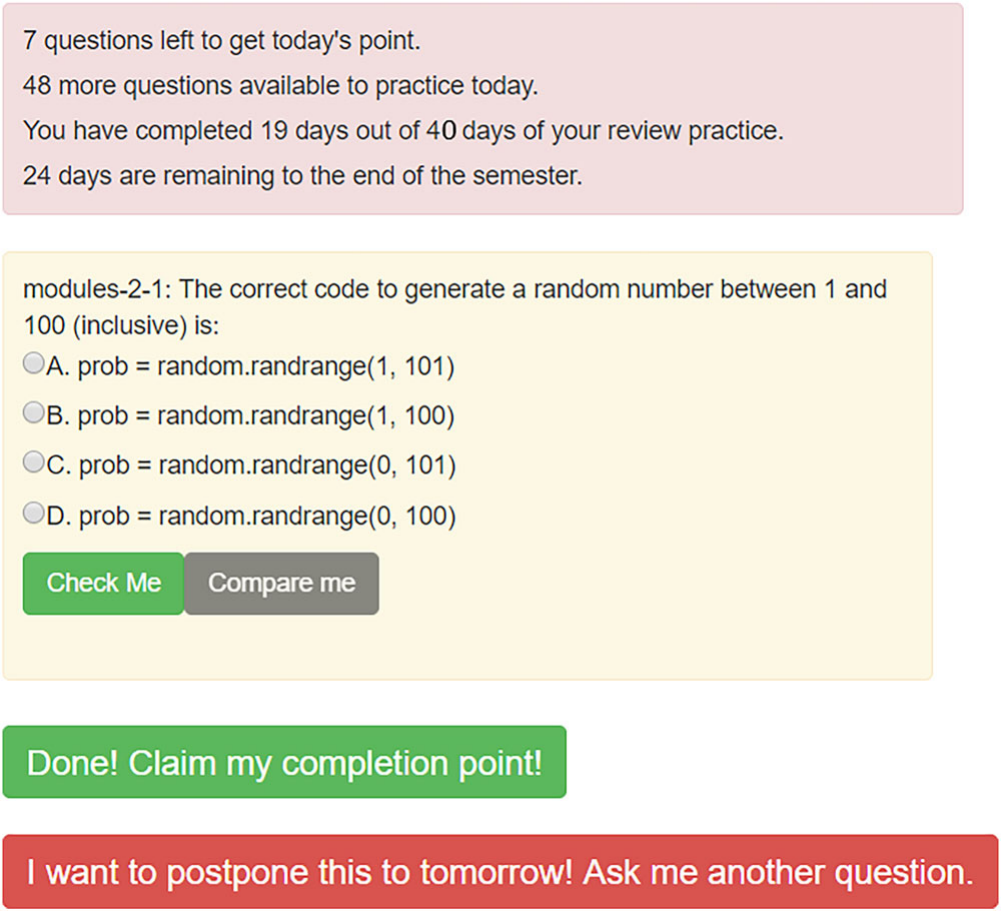
The Practice Tool Sample Interface. The figure displays the interface of the practice tool as seen by a student. Questions are selected from topics scheduled for practice or review on that day, with adaptive scheduling based on performance.

Both experiments used the same technical apparatus, a retrieval practice tool that is built into the Runestone online textbook platform^51^. It presents a student with one question at a time, drawn from a bank of questions associated with a textbook, divided into topics defined by that textbook. Once a topic is covered in the course, it is scheduled to be visited in the practice tool. The questions were of two types^57^:

- **214 Multiple-choice** questions (e.g, Fig. 5); the median time to answer these was 19 seconds.
- **186 Active-code** questions, where students could write and execute code to get immediate feedback about the quality and accuracy of their answers (see Supplementary Fig. 1 for an example); the median time to answer these questions was 50 seconds.

The final exam consisted of analogous but new multiple-choice and coding questions, as well as some questions of a different type that asked students to predict what would print out as a result of executing a provided block of code. The exam was delivered on paper, so they could not run any code.

When a student loads the web page for the practice tool (see Fig. 5), they see a question selected from one of the topics scheduled to be visited or revisited that day. If a student answers questions from a topic quickly and correctly, the topic is scheduled to be revisited many days later; if not, it is scheduled for sooner. The practice tool employs an adaptive spacing algorithm inspired by the SuperMemo 2 algorithm^58^. This algorithm schedules topics to be revisited at increasing intervals based on the student’s performance, effectively implementing an expanding spacing schedule. When a student answers questions from a topic correctly and quickly, the interval before that topic is scheduled again increases. Conversely, if a student struggles with a topic, it is scheduled for review sooner. This adaptive approach optimizes learning by reinforcing material just as it is on the verge of being forgotten. It is important to note that this adaptive spacing algorithm inherently interleaves topics by cycling through various subjects based on individual performance. Because both the Counting Days and Counting Questions groups utilize the same interleaving mechanism, any differences in learning outcomes can be attributed solely to the additional spacing incentive provided in the Counting Days condition. The algorithm spreads topics over multiple sessions but does not automatically induce students to engage in many practice sessions on different days.

### Experiment 1: Within-Class

The first experiment involved students in a single offering of an introductory Python programming course at a large public university. Students were randomized to either the counting days condition or a control condition. The textbook’s question bank had 400 questions covering 177 topics.

Table 6 shows the participant funnel and exclusions in the within-class experiment. A total of 199 participants were randomized to one of the two conditions. Each student was assigned independently, based on a virtual coin flip for that student, the first time that they opened the practice tool.

**Table 6:** Participant funnel in the within-class experiment

	Total	Counting Days	Counting Questions
All participants who ever used the practice tool (# who were randomized)	199	110	89
All students with prior GPA	159	89	70
Completed course final exam	157	89	68
Took course for grade	143	83	60

The funnel traces participants through key stages, ending with the 143 students whose data was used for the analyses reported in this paper.

Forty students had no prior GPA because they were in their first semester. Since our focus is the correlation between the prior GPA and final exam score, we excluded these forty students in the second row. Note that after excluding those students, a few freshmen remained. We treat them as sophomores when reporting demographics because they had taken courses in a previous semester and had prior GPA.

The third row shows that two students, both under the counting questions condition, dropped the course or did not take the final exam. Since our primary outcome measure was final exam score, these students are excluded from the analysis.

Finally, we excluded fourteen students (six from the counting days condition and eight from counting questions) who took the course pass/fail rather than for a letter grade. The rationale for exclusion was that such students might not be affected by the grading incentive in the same way as other students. The course was a pre-requisite for entry into the Information Science major. Those who took the course pass/fail were from other programs where the course was not required. Among them were some students with very high GPAs, but since the course would not count towards their required credits in their program, they chose to take it pass/fail.

Table 7 shows the demographic breakdown of students. The last (right-most) column reports the results of a group *χ*^2^ test to check for balance of the corresponding variable across the two conditions. Table 8 shows the breakdown of prior GPA. The last column reports the results of an F-test. None of the tests are statistically significant (*p* > 0.05), indicating no evidence to reject the randomization balance of the specified variables across the experimental groups.

**Table 7:** Participant demographics in the within-class experiment

**Gender**		Female	Male		*χ*^2^ = 0.382
	Counting Days	54 (65.1%)	29 (34.9%)		
	Counting Questions	36 (60.0%)	24 (40.0%)		
**Ethnicity**		White	Asian	Other Ethnicity	*χ*^2^ = 4.774
	Counting Days	50 (60.2%)	21 (25.3%)	12 (14.5%)	
	Counting Questions	37 (61.7%)	12 (20.0%)	11 (18.3%)	
**Class Level**		Sophomore	Junior	Senior	*χ*^2^ = 0.340
	Counting Days	51 (61.4%)	17 (20.5%)	15 (18.1%)	
	Counting Questions	39 (65.0%)	10 (16.7%)	11 (18.3%)	
**Language Proficiency**		Non-Native English	Native English		*χ*^2^ = 2.313
	Counting Days	17 (20.5%)	66 (79.5%)		
	Counting Questions	19 (31.7%)	41 (68.3%)		

The last column reports the results of a *χ*^2^ test, checking for evidence of imbalance of these variables across the two experimental conditions. None of the tests are statistically significant (*p* > 0.05).

**Table 8:** Prior GPA by condition in the within-class experiment

	Group	Min	Median	Max	Mean	SD	Test
**Prior GPA**	Counting Days	1.967	3.626	3.954	3.482	0.404	*F* = 2.742
	Counting Questions	1.903	3.469	4.000	3.358	0.487	

The last column reports the results of an F-test, checking for evidence of imbalance of prior GPA across the two experimental conditions. The result is not statistically significant (*p* > 0.05).

Ten percent of the course grade, 100 points, was based on the use of the practice tool. Students were randomly assigned to one of two treatment conditions that varied the spacing incentive.

In the Counting Questions (control) condition, each student could earn a quarter of a point per question for correctly answering up to 400 questions. Above the question that was presented, the student saw text like the following:

- 48 more questions are available to practice today.
- So far, you've received 60 points out of 100 possible points for answering 240 questions out of 400 questions to complete your practice.
- 30 days are remaining until the end of the practicing period this semester.

In the Counting Days (treatment) condition, each student could earn the same 100 points in increments of 2.5 points for each day that they correctly answered ten practice questions, for up to 40 days. If they ran out of topics scheduled for that day before reaching ten correct answers, they also earned 2.5 points; at the extreme, a student who had no topics scheduled could earn the day’s points just by opening the web page. Students did not earn additional points for correctly answering more than ten questions on a single day.

While having students practice every day might be ideal for learning, we recognized that students have other obligations. Thus, we configured it so that students could earn the maximum available points with 40 days of activity, an average of about three days per week. We chose ten questions as the threshold to earn a day’s points because it was a round number that could be completed in a reasonable amount of time, typically 5-15 minutes.

In the Counting Days treatment, when a student loaded the web page for the practice tool, above the question that was presented, they saw text like the following:

- 7 questions left to get today's point.
- 48 more questions are available to practice today.
- So far, you've received 60 points out of 100 possible points for completing 24 days out of 40 days of your review practice.
- 30 days are remaining until the end of the practicing period this semester.

### Experiment 2: Between-Instructors

In the second, between-instructor experiment, all instructors covered the same fundamentals of the Java programming course, using identical learning materials, practice questions, and weekly assignments. Instructors assigned to the Counting Days condition configured the points per day of practice, the number of questions to practice per day to get the practice points, and the maximum number of days that students could possibly get points for. Instructors in the Counting Questions condition configured the points per question and a maximum number of questions students could earn points for. Table 9 provides summary statistics for the number of students and instructor configurations.

**Table 9:** Student counts and instructor configurations in the between-instructors experiment

Variable	Condition	Min	Median	Mean	Max	SD
# of Students	Counting Days	5	19	25.92	84	20.68
	Counting Questions	5	23	23.82	54	12.59
Max # of Days to Earn Points	Counting Days	8	50	63.17	200	37.54
Points Per Day of Practice	Counting Days	1	2	3.97	100	9.93
# of Questions to Earn Day’s Points	Counting Days	2	7	7.47	20	2.87
Points Per Question Practiced	Counting Questions	0.1	0.2	0.44	2	0.38
Max # of Questions	Counting Questions	50	500	630.99	10,000	1191.93

Descriptive statistics for the number of students and instructor configurations in the between-instructors experiment.

Randomization occurred at the level of instructors. A total of 71 high school and college instructors taught 97 introductory computer programming courses to 1, 648 students. If an instructor taught multiple classes in the same semester or across different semesters, all their students were assigned the same conditions.

In the counting days treatment group, 44 instructors taught 58 courses with a total of 994 students. Each instructor determined the number of questions needed to earn a day’s points, the number of points per day, and the maximum number of days to earn points in their courses. In the counting questions group, 27 instructors taught 39 courses with 654 students. Each instructor determined for their courses the number of points per question and the maximum number of questions for which students could earn points. The difference in sample sizes between the two conditions is due to independent coin flips for each instructor and because, post-randomization, some instructors did not teach any courses using the tool. Under the Counting Days condition, class sizes ranged from a minimum of 5 students to a maximum of 79 students, while under the Counting Questions condition, class sizes varied from a minimum of 5 students to a maximum of 89 students.

### Informed consent

These two experiments were approved by the University of Michigan Institutional Review Board (IRB) under HUM00141422 and HUM00144387. Students in the within-class experiment were informed about the nature of the research. They did not have a choice about being randomized to treatment or control but had the option, at any time, to request that their data not be used in the research; no one exercised this option.

Instructors in the between-instructors experiment were informed about the nature of the research. They did not have a choice about being randomized to treatment or control but had the option, at any time, to request that their data not be used in the research; no teacher exercised this option.

## Supplementary information

Supplementary Information

Supplementary Dataset 1

## Data Availability

We are not comfortable publishing the raw datasets for the within-class experiment, even without individual student identifiers, because of the risk of reidentification. There were 199 students; with several demographic characteristics and prior GPA, we think that someone who had other sources of information such as the class roster might be able to identify some individuals, revealing sensitive personal information such as their final exam score, prior GPA, and diligence in completing course activities. The anonymized dataset for the between-instructor experiment is available as Supplementary Dataset 1.

## Code availability

The supplementary information document includes a printout of the Stata do file and R Studio notebook that were used to conduct the analyses.
