# Supplementary material for: Counting days is a spacing incentive that unlocks the potential of low GPA students
Source: NPJ Sci Learn. 2025 Jun 5;10:35. doi: 10.1038/s41539-025-00322-5 (PMC12141441; doi:10.1038/s41539-025-00322-5)
Supplement: Supplementary file 1 — Supplementary Information [file 41539_2025_322_MOESM1_ESM.pdf]

Counting Days is a Spacing Incentive that Unlocks the Potential of Low GPA Students - Supplementary Information

Iman YeckehZaare[1*] and Paul Resnick[2]

[1*]MIT Center for Collective Intelligence, Sloan School of Management, Massachusetts Institute of Technology, 245 First Street, E94, Cambridge, 02142, MA, USA.
[2]School of Information, University of Michigan, 105 S State St., Ann Arbor, 48109, MI, USA.

*Corresponding author(s). E-mail(s): oneman@mit.edu;
Contributing authors: presnick@umich.edu;

Supplementary Figure 1: Interactive sample active-code in the practice tool.

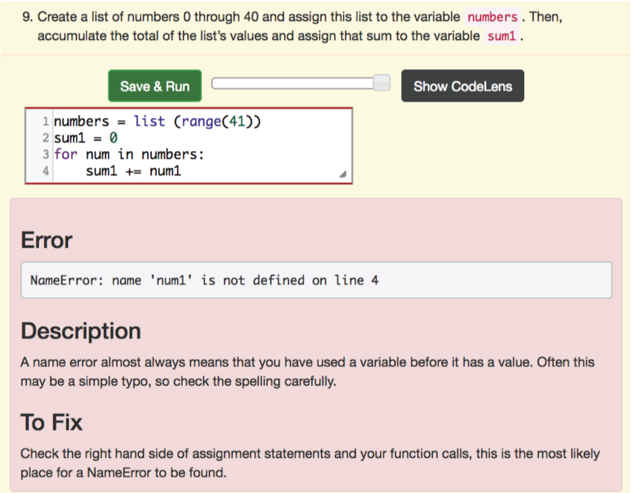

Students are able to write code in the box provided. When they click the Save & Run button, the code is executed. If there is a runtime error, an error message is shown, as in the Figure. If the code runs to completion, an autograder checks whether the code correctly accomplishes the task specified.

Supplementary Table 1: Summary statistics of the numeric variables in the within-class experiment, only for students who took the course Pass/Fail.

	Group	Min	Median	Max	Mean	SD
**Final Exam Score (in %)**	Counting Days	51.5	72.4	83	69.7	13.1
	Counting Questions	16.0	75.5	96	70.1	25.8
**Practice Days**	Counting Days	27	40	44	37.7	6.0
	Counting Questions	1	14	22	12.9	7.3
**Practiced Questions**	Counting Days	287	439	574	445.0	94.2
	Counting Questions	2	400	412	313.8	154.0

This table shows descriptive statistics for the fourteen students omitted from the analysis because they took the course pass/fail. Although there are too few students to perform statistical tests, there are no obvious differences in outcomes between these students and the students who took the course for grades. Due to the small number of observations, we do not report statistical tests between the two conditions.

Supplementary Note 1: Hypothesis Testing Results Robustness Checks

As a robustness check on the beta and negative binomial regression results, we estimated the models excluding the demographic control variables. There were no major differences in the magnitude or significance of the coefficients. See Supplementary Table 2.

As a final robustness check on the beta and negative binomial regression results, we estimated the models including the fourteen students who took the class pass-fail. See Supplementary Table 3. Comparing with Table 3, the effect size on final exam score of counting days and its interaction term with GPA were both reduced in magnitude and no longer statistically significant. There were also substantial changes in the regression estimates for days practiced and questions practiced. This may be due to the noise introduced by students who did not care much about getting the grading points from the practice tool or whose performance on the exam reflected prior knowledge rather than learning in the course. For example, there was at least one student from the computer science department who took the course pass-fail under the Counting Questions condition, did not answer any practice questions nor attend class, but got a high final exam score.

Supplementary Table 2: Beta regression results of the within-class experiment.

	**Final Exam Score (Beta)**	**Days Practiced (Neg. Bin.)**	**Questions Practiced (Neg. Bin.)**
**Counting Days (vs. Questions)**	2.625** (0.842)	1.360*** (0.316)	0.139 (0.184)
**GPA**	0.657*** (0.161)	0.248*** (0.074)	0.097** (0.037)
**Counting Days × GPA**	-0.726** (0.245)	-0.168 (0.091)	-0.027 (0.053)
**[Intercept]**	-0.713 (0.534)	2.011*** (0.255)	5.645*** (0.127)
**[Parameter]**	**Scale:** 13.573*** (1.594)	**Alpha:** 0.010 (0.007)	**Alpha:** 0.017*** (0.002)
**Log Likelihood**	144.049	-480.101	-779.858
**# of Obs.**	143	143	143
χ^2	10.485 ***	191.602	145.651

This table displays the beta regression results for the within-class experiment, conducted as a robustness check by excluding demographic control variables. The table reports regression coefficients for three outcome variables: Final Exam Score (Beta regression), Days Practiced (Negative Binomial regression), and Questions Practiced (Negative Binomial regression). Key predictors include "Counting Days," "GPA," and their interaction term "Counting Days × GPA." Standard errors are shown in parentheses. Significance levels are denoted as follows: *** $p < 0.001$; ** $p < 0.01$; * $p < 0.05$. Model parameters, including scale and alpha, as well as log-likelihood values, the number of observations, and Chi-squared statistics, are also reported. Results demonstrate the robustness of the findings when controls are omitted.

Supplementary Table 3: Regression results with controls of the within-class experiment, *including pass-fail students*

	**Final Exam Score (Beta)**	**Days Practiced (Neg. Bin.)**	**Questions Practiced (Neg. Bin.)**
**Counting Days (vs. Questions)**	1.524<br>(0.880)	1.487***<br>(0.307)	0.186<br>(0.294)
**GPA**	0.604***<br>(0.165)	0.170*<br>(0.070)	0.062<br>(0.058)
**Counting Days × GPA**	-0.392<br>(0.256)	-0.195*<br>(0.088)	-0.032<br>(0.085)
**Female (vs. Male)**	-0.112<br>(0.126)	0.151***<br>(0.040)	0.046<br>(0.041)
**Asian (vs. White)**	0.213<br>(0.150)	-0.069<br>(0.044)	-0.036<br>(0.048)
**NonWhiteOrAsian (vs. White)**	-0.038<br>(0.150)	-0.077<br>(0.048)	-0.065<br>(0.050)
**Junior (vs. Sophomore)**	0.213<br>(0.155)	-0.059<br>(0.046)	-0.048<br>(0.050)
**Senior (vs. Sophomore)**	0.085<br>(0.137)	-0.094*<br>(0.043)	-0.050<br>(0.045)
**NonNativeEnglish (vs. NativeEnglish)**	0.375**<br>(0.140)	0.005<br>(0.042)	0.023<br>(0.044)
**[Intercept]**	-0.789<br>(0.566)	2.208***<br>(0.243)	5.745***<br>(0.199)
**[Parameter]**	**Scale:**<br>11.692***<br>(1.307)	**Alpha:**<br>0.008<br>(0.007)	**Alpha:**<br>0.048***<br>(0.006)
**Log Likelihood**	144.452	-525.984	-938.327
**# of Obs.**	157	157	157
χ^2	17.941<br>***	221.274	183.743

Coefficients are presented as log odds ratios with standard errors in parentheses. Significant coefficients are denoted as follows: *** $p < 0.001$; ** $p < 0.01$; * $p < 0.05$. Models were estimated for three dependent variables: Final Exam Score (Beta regression), Days Practiced (Negative Binomial regression), and Questions Practiced (Negative Binomial regression).

Supplementary Note 2: GSEM Results

Supplementary Table 4 shows the results of our Generalized Structural Equation Model (GSEM) estimation for the within-class experiment, with four dependent variables: final exam score, days practiced, questions practiced, and GPA. We used a Beta regression to estimate the final exam score after transforming it to the range $(0, 1)$. We used Negative Binomial regressions to estimate the number of days and questions practiced. We used an OLS to estimate the GPA. We performed this analysis only in STATA using the following command:

```
gsem (c.practice_days c.practice_count c.gpa ///
   c.gpa#c.practice_days c.gpa#c.practice_count ///
   1.female 1.junior 1.senior 1.asian 1.nonwhiteorasian ///
   1.nonnativeenglish -> final_0_1, family(beta) link(logit)) ///

   (i.counting_days c.gpa i.counting_days#c.gpa 1.female 1.junior ///
   1.senior 1.asian 1.nonwhiteorasian 1.nonnativeenglish ->  ///
   practice_days, nbreg) ///

   (c.practice_days c.gpa 1.female 1.junior 1.senior 1.asian ///
   1.nonwhiteorasian 1.nonnativeenglish -> practice_count, nbreg) ///

   (1.female 1.junior 1.senior 1.asian 1.nonwhiteorasian ///
   1.nonnativeenglish -> gpa, )
```

Supplementary Table 4: Results of the GSEM Estimation.

	Final Exam Score	Days Practiced	Questions Practiced	GPA
**Counting Days (vs. Questions)**		a: 1.638*** (0.305)		
**Counting Days#GPA**		b: −0.247** (0.088)		
**Days Practiced**	b: 0.088* (0.039)		i: 0.058* (0.027)	
**Days Practiced#GPA**	e: −0.024* (0.011)			
**Questions Practiced**	h: −0.004 (0.007)			
**Questions Practiced#GPA**	g: 0.001 (0.002)			
**GPA**	j: 0.844 (0.797)	c: 0.223** (0.072)	f: −0.001 (0.025)	
**Female (vs. Male)**	−0.149 (0.124)	0.133*** (0.040)	−0.059* (0.029)	−0.072 (0.095)
**Asian (vs. White)**	0.122 (0.141)	−0.091* (0.044)	−0.023 (0.030)	−0.031 (0.084)
**NonWhiteOrAsian (vs. White)**	−0.026 (0.146)	−0.066 (0.048)	0.001 (0.026)	3.271*** (0.075)
**Junior (vs. Sophomore)**	0.313* (0.149)	−0.059 (0.045)	0.007 (0.030)	−0.072 (0.088)
**Senior (vs. Sophomore)**	0.391** (0.149)	−0.036 (0.046)	−0.029 (0.028)	0.020 (0.097)
**NonNativeEnglish (vs. NativeEnglish)**	0.340* (0.132)	0.011 (0.041)	5.696*** (0.090)	2.779 (0.118)
**[Intercept]**	−1.101 (2.564)	2.056*** (0.245)	0.316*** (0.075)	−5.587 (1.572)
**[Paremeter]**	**logs:** 2.779 (0.118)	**lnalpha:** −5.587 (1.572)	**lnalpha:** −4.283 (0.142)	**var(e.gpa):** 0.167 (0.020)

Dependent variable indicated in the column header. Standard errors are in parentheses. Statistically significant coefficients are marked with asterisks: *** $p < 0.001$; ** $p < 0.01$; * $p < 0.05$. Parameters labeled with letters (e.g., a, b, c) correspond to specific paths in the structural model.

Supplementary Figure 2: Predictive Margins of Days Practiced with 95% CIs for the within-class experiment. Estimated effect of GPA on final exam scores for different numbers of days practiced.

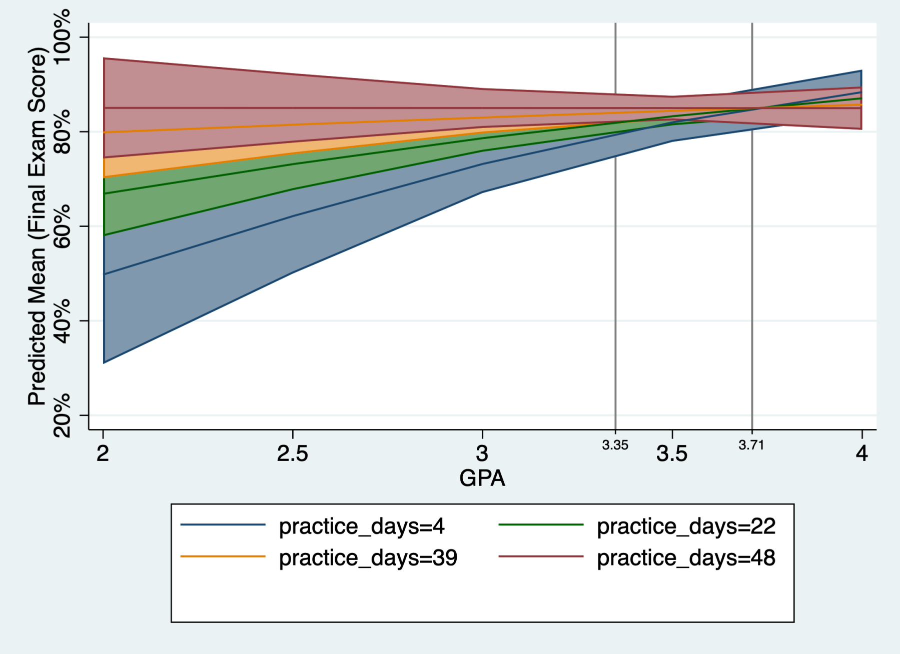

This figure shows the effects of more days of practice on the correlation between GPA and exam scores, regardless of treatment condition, for the within-class experiment. More days of practice lowered the correlation between GPA and final exam score, as indicated by the flatter curves for more days of practice.

Supplementary Note 3: Analysis of GPA correlation with final exam scores in other semesters

In three other semesters of the same course where we conducted the within-class experiment, all students were assigned to the counting days condition. As a robustness check, Supplementary Figures 3, 4, and 5 reproduce the counting days part of Figure 1 from the main study for these other semesters. The results do not paint a clear picture. The correlation between GPA and final exam scores was very small in one of the semesters (Supplementary Figure 5), consistent with the results of our within-semester experiment. However, the correlation was much larger in the other two semesters.

Supplementary Figure 3: Adjusted Predictions with 95% CIs in Winter 2018. Estimated effect of GPA on final exam scores based on the Beta regression model predictions.

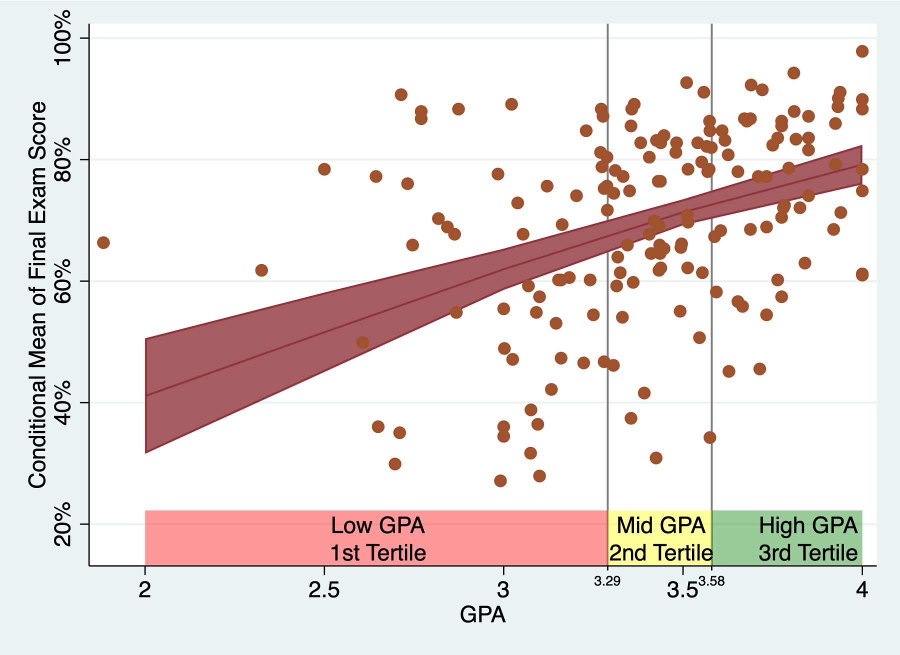

This figure shows the adjusted predictions of final exam scores based on Beta regression models are plotted against students' cumulative GPA for the Winter 2018 semester. The solid line represents the estimated effect of GPA on final exam scores, while the shaded area indicates the 95% confidence intervals. All students were assigned to the counting days condition during this semester.

Supplementary Figure 4: Adjusted Predictions with 95% CIs in Winter 2019. Estimated effect of GPA on final exam scores based on the Beta regression model predictions.

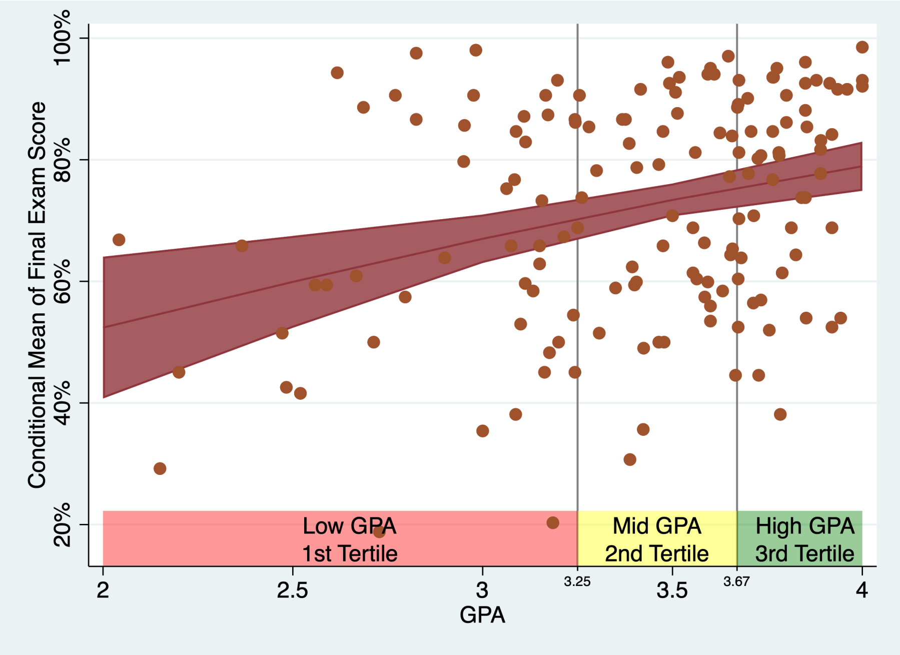

This figure displays the adjusted predictions of final exam scores derived from Beta regression models as a function of students' cumulative GPA in the Winter 2019 semester. The solid line illustrates the estimated effect, and the shaded region represents the 95% confidence intervals. All students participated in the counting days condition in this semester.

Supplementary Figure 5: Adjusted Predictions with 95% CIs in Fall 2019. Estimated effect of GPA on final exam scores based on the Beta regression model predictions.

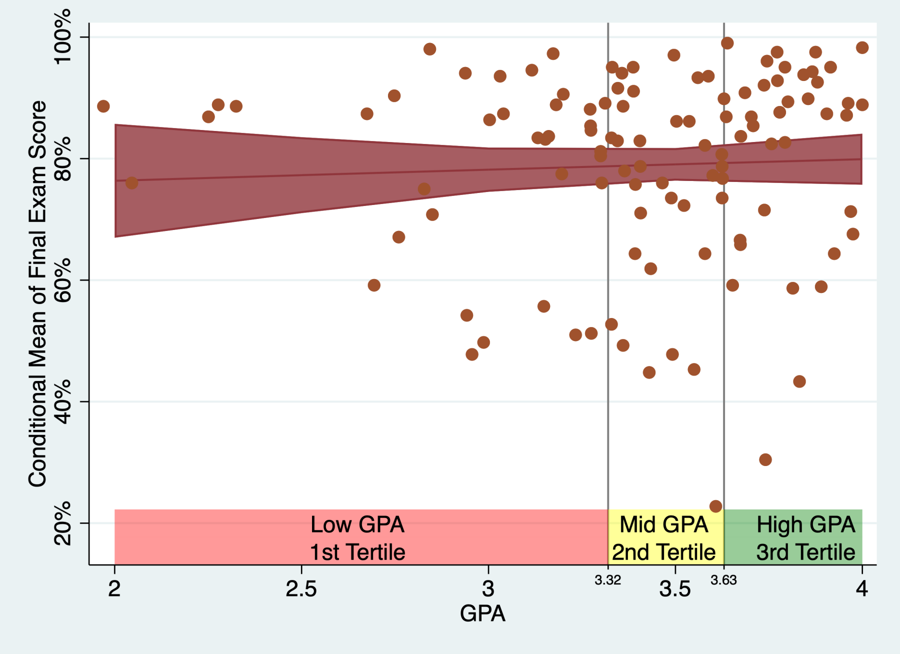

This figure shows the adjusted predictions of final exam scores using Beta regression models plotted against students' cumulative GPA for the Fall 2019 semester. The solid line depicts the estimated effect of GPA on final exam scores, with the shaded area indicating the 95% confidence intervals. All students were in the counting days condition during this semester. Notably, this semester exhibited no significant correlation between GPA and final exam scores, consistent with the findings from our within-semester experiment.

Supplementary Note 4: Stata Do File

~\Desktop\Data and Analysis\Fall2018_Data\Counting Days - Nature 2024 - GSEM_Stata.do

```
// Install the package to generate LaTeX code for tables.
// ssc install estout, replace
// Install the package to generate Word Doc files for tables.
// ssc install asdoc, replace
// Install the package to be able to use the nvals command.
// ssc install egenmore, replace

* Clear the memory before starting. *
clear


/***
*** Daily Practice Analysis ***
***/

* Clear the memory before starting. *
clear

* Specify the path *
global mypath "[YOUR FILES PATH SHOULD GO HERE.]"
cd "$mypath"

// Load the dataset again.
import delimited "DailyPracticeData.csv"

gen p_date = date(practice_date, "MD20Y")
tsset  p_date, daily
list practice_date
list p_date
list practice_count_diff

/***
*** Fig. 3.
How Students Under the Counting Days and Questions Conditions Procrastinate Differently
***/

scatter practice_count_diff p_date, ///
   tlabel(01oct2018 29oct2018 16nov2018 03dec2018 19dec2018, format(%tdmd)) ///
   tline(29oct2018, lcolor(blue)) tline(19dec2018, lcolor(green)) ///
   yline(0) ///
   t1title("More Answered in Counting Days Condition                                             ") ///
   b1title("More Answered in Counting Questions Condition                                     ") ///
   l1title("Difference in # of Questions Answered") ///
   ytitle("") ///
   ttitle("")


* Clear the memory before starting. *
clear

// Load the dataset.
cd "$mypath"
import delimited "CountingDaysFourSemesterData.csv"

/***
*** Fall 2018 experiment Analysis ***
***/

gen counting_days = 0
replace counting_days = 1 if spacing == "Counting Days"

/***
*** Table 2
Apply the data inclusion criteria to generate the student funnel
***/

gen practice_date = date(practicedate, "YMD")
summarize practice_date

* Generate a count of distinct practice_date observations for each user_id
egen practice_days = tag(user_id practice_date) if user_id != . & q > 0 & term == "FA 2018"

// Only consider the data collected in the experiment semester, Fall 2018.
```

```
// Generate the student-level dataframe. Each row should represent a single student.
collapse (first) counting_days (first) gradingtype (first) final (first) final_0_1 (first) gpa (first) sex (first) male (first) female (first) ethnicity (first) white (first) asian (first) nonwhiteorasian (first) nonnativeenglish (first) freshman (first) sophomore (first) junior (first) senior (first) academiclevel (count) practice_count = final (sum) practice_time = duration (sum) practice_days if user_id != . & q > 0 & term == '2018", by(user_id)

summarize practice_days
replace practice_time = practice_time / 3600
summarize practice_time

// The GPA field was populated from an inner join query with student data from
// the registrar. GPA field is blank when the student was not registered for
// this course in this semester (i.e., TAs and other system testers).
tabulate counting_days
tabulate counting_days if gpa > 0 & gpa <= 4

// We exclude those students who did not take the final exam; i.e., withdrew the
// course (at the end of the semester, after paying the tuition) or failed.
tabulate counting_days if final > 0 & final <= 100 & gpa > 0 & gpa <= 4

//We exclude those students who took the course for purposes other than getting
// a letter grade, such as auditing.
tabulate counting_days if gradingtype == "Graded" & final > 0 & final <= 100 ///
& gpa > 0 & gpa <= 4

/***
*** Main Analysis
***/
// The GPA field was populated from an inner join query with student data from
// the registrar. GPA field is blank when the student was not registered for
// this course in this semester (i.e., TAs and other system testers).

// We exclude those students who did not take the final exam; i.e., withdrew the
// course (at the end of the semester, after paying the tuition) or failed.

//We exclude those students who took the course for purposes other than getting
// a letter grade, such as auditing.
keep if gradingtype == "Graded" & final > 0 & final <= 100 & ///
gpa > 0 & gpa <= 4

// After excluding those who have no GPA, a few Freshmen remained. We treat them
// as Sophomores because they had taken courses in the previous semester.
replace sophomore = 1 if freshman == 1
replace academiclevel = "Sophomore" if freshman == 1

// Calculate the GPA standard deviation.
sum gpa

// Define min_gpa as the minimum GPA.
local min_gpa = r(min)

// Define mean_gpa as the average GPA.
local mean_gpa = r(mean)

// Define max_gpa as the maximum GPA.
local max_gpa = r(max)

// Define low_gpa as one standard deviation below the mean GPA.
local low_gpa = r(mean) - r(sd)

// Define low_gpa as one standard deviation above the mean GPA.
local high_gpa = r(mean) + r(sd)

// Define the gap terciles.
pctile gap_terciles = gpa, nq(3)
// GPA 1st tercile.
local gpa_1st = round(r(r1), 0.01)
// GPA 2nd tercile.
local gpa_2nd = round(r(r2), 0.01)
local gpa_mid = round((`gpa_1st' + `gpa_2nd')/2, 0.01)

/***
*** Fig. 2
***/

betareg final_0_1 i.counting_days c.gpa c.gpa#i.counting_days ///
```

```
1.female 1.junior 1.senior 1.asian 1.nonwhiteorasian 1.nonnativeenglish

margins i.counting_days, at(gpa=(2(0.5)4)) atmeans

marginsplot, ///
addplot( ///
scatteri 0.2 2  0.2 `gpa_1st', connect(line) mcolor(none) lwidth(vvvthick) ///
lcolor(red%40) || ///
scatteri 0.1486 2  0.1486 `gpa_1st', connect(line) mcolor(none) lwidth(vvvthick) ///
lcolor(red%40) || ///
scatteri 0.2 `gpa_1st' 0.2 `gpa_2nd', connect(line) mcolor(none) lwidth(vvvthick) ///
lcolor(yellow%40) || ///
scatteri 0.1486 `gpa_1st' 0.1486 `gpa_2nd', connect(line) mcolor(none) lwidth(vvvthick) ///
lcolor(yellow%40) || ///
scatteri 0.2 `gpa_2nd' 0.2 4, connect(line) mcolor(none) lwidth(vvvthick) ///
lcolor(green%40) || ///
scatteri 0.1486 `gpa_2nd'  0.1486 4, connect(line) mcolor(none) lwidth(vvvthick) ///
lcolor(green%40) || ///
scatter final_0_1 gpa ///
if counting_days == 0, mcolor(navy) || ///
scatter final_0_1 gpa ///
if counting_days == 1, mcolor(maroon) ///
legend(order(1 "Counting Questions" 2 "Counting Days")) ///
text(0.2 2.65 "Low GPA" 0.2 `gpa_mid' "Mid GPA" 0.2 3.85 "High GPA" ///
    0.1486 2.65 "1st Tercile" 0.1486 `gpa_mid' "2nd Tercile" ///
    0.1486 3.85 "3rd Tercile")) ///
ylab(.2 "20%" .4 "40%" .6 "60%" .8 "80%" 1 "100%") ///
xmlabel(`gpa_1st' "`gpa_1st'" `gpa_2nd' "`gpa_2nd'") ///
recast(line) recastci(rarea) ///
xline(`gpa_1st' `gpa_2nd', lcolor(gray)) ///
title("") ///
xtitle("GPA") ytitle("Conditional Mean of Final Exam Score")


/***
*** Table 4, 5
***/
// Summary statistics and randomization check.

// clear the stored estimates
est clear

// Final exam score
estpost tabstat final, by(counting_days) ///
statistics(min median max mean sd) columns(statistics)
esttab using TableD2Final.tex, cells("min(fmt(1)) p50(fmt(3)) max(fmt(0)) mean(fmt(3)) sd(fmt(3))") mtitle(Table D2. Final) label replace
asdoc tabstat final, by(counting_days) ///
statistics(min median max mean sd) title(Table D2 Grouped) dec(3) replace
asdoc tabstat final, by(counting_days) ///
statistics(min median max mean sd) title(Table D2) dec(3),

reg final counting_days

// Practice Days
estpost tabstat practice_days, by(counting_days) ///
statistics(min median max mean sd) columns(statistics)
esttab using TableD2DaysPracticed.tex, cells("min(fmt(0)) p50(fmt(0)) max(fmt(0)) mean(fmt(3)) sd(fmt(3))") mtitle(Table D2. Days Practiced) label
replace
asdoc tabstat practice_days, by(counting_days) ///
statistics(min median max mean sd) title(Table D2. Days Practiced) dec(3)
asdoc tabstat practice_days, ///
statistics(min median max mean sd) title(Table D2. Days Practiced) dec(3)

reg practice_days counting_days

// Practiced Questions
estpost tabstat practice_count, by(counting_days) ///
statistics(min median max mean sd) columns(statistics)
esttab using TableD2PracticedQuestions.tex, cells("min(fmt(0)) p50(fmt(0)) max(fmt(0)) mean(fmt(3)) sd(fmt(3))") mtitle(Table D2. Practiced Questi
label replace
asdoc tabstat practice_count, by(counting_days) ///
statistics(min median max mean sd) title(Table D2. Practiced Questions) dec(3)
asdoc tabstat practice_count, ///
statistics(min median max mean sd) title(Table D2. Practiced Questions) dec(3)

reg practice_count counting_days
```

```
// GPA
estpost tabstat gpa, by(counting_days) ///
statistics(min median max mean sd) columns(statistics)
esttab using TableD2GPA.tex, cells("min(fmt(3)) p50(fmt(3)) max(fmt(3)) mean(fmt(3)) sd(fmt(3))") mtitle(Table D2. GPA) label replace
asdoc tabstat gpa, by(counting_days) ///
statistics(min median max mean sd) title(Table D2. GPA) dec(3)
asdoc tabstat gpa, ///
statistics(min median max mean sd) title(Table D2. GPA) dec(3)

reg gpa counting_days

//
// Table 3
//

* Start with clean slate
flexmat reset

*Store the first table in location 1
local next 1

count if counting_days == 1
local total_days = `r(N)'
count
local total_questions = `r(N)' - `total_days'

flexmat addparts, title(Table D3)  location(`next')
flexmat addcell, data(Total) row(1) col(2) location(`next')
flexmat addcell, data(Counting Days) row(1) col(3) location(`next')
flexmat addcell, data(Counting Questions) row(1) col(4) location(`next')
flexmat addcell, data(X2 Test) row(1) col(5) location(`next')

// Sex: Female
tabulate sex counting_days, chi2
local total_N = `r(N)'
local r_chi2 = string(`r(chi2)', "%7.3f")
local r_chi2_p = ""
if `r(p)' < 0.05 local r_chi2_p = "*"
if `r(p)' < 0.01 local r_chi2_p = "**"
if `r(p)' < 0.001 local r_chi2_p = "***"
local r_chi2_star = "`r_chi2' `r_chi2_p'"

count if female == 1
local total_1_perc = string((`r(N)' * 100) / `total_N', "%7.1f")
local total_1 = "`r(N)'\; `total_1_perc'%"

count if female == 1 & counting_days == 1
local total_1_days_perc = string((`r(N)' * 100) / `total_days', "%7.1f")
local total_1_days = "`r(N)'\; `total_1_days_perc'%"

count if female == 1 & counting_days == 0
local total_1_questions_perc = string((`r(N)'*100) / `total_questions', "%7.1f")
local total_1_questions = "`r(N)'\; `total_1_questions_perc'%"

// eststo clear
// estadd local cat_var "Female"
// estadd local total_1_1 "`r(N)'   `total_1_perc'%"
// estadd local total_1_days_1 "`r(N)'   `total_1_days_perc'%"
// estadd local total_1_questions_1 "`r(N)'   `total_1_questions_perc'%"
// estadd local test_1 "X2=`r_chi2' `r_chi2_p'"
//
// esttab using TableD3.tex, scalars ("cat_var total_1_1 total_1_days_1 total_1_questions_1 test_1") mtitle(Table D3) label replace ///
// noobs nonumber collabels(none) gaps

flexmat addrow, data(Female,`total_1',`total_1_days',`total_1_questions',`r_chi2_star') ///
dec(3) row(2) col(1) location(`next')

// Sex: Male
count if male == 1
local total_1_perc = string((`r(N)' * 100) / `total_N', "%7.1f")
local total_1 = "`r(N)'\; `total_1_perc'%"

count if male == 1 & counting_days == 1
local total_1_days_perc = string((`r(N)' * 100) / `total_days', "%7.1f")
local total_1_days = "`r(N)'\; `total_1_days_perc'%"
```

```
count if male == 1 & counting_days == 0
local total_1_questions_perc = string((`r(N)'*100) / `total_questions', "%7.1f")
local total_1_questions = "`r(N)'\; `total_1_questions_perc'%"

flexmat addrow, data(Male,`total_1',`total_1_days',`total_1_questions',`r_chi2_star') ///
dec(3) row(3) col(1) location(`next')

// White
tabulate ethnicity counting_days, chi2
local total_N = `r(N)'
local r_chi2 = string(`r(chi2)', "%7.3f")
local r_chi2_p = ""
if `r(p)' < 0.05 local r_chi2_p = "*"
if `r(p)' < 0.01 local r_chi2_p = "**"
if `r(p)' < 0.001 local r_chi2_p = "***"
local r_chi2_star = "`r_chi2' `r_chi2_p'"

count if white == 1
local total_1_perc = string((`r(N)' * 100) / `total_N', "%7.1f")
local total_1 = "`r(N)'\; `total_1_perc'%"

count if white == 1 & counting_days == 1
local total_1_days_perc = string((`r(N)' * 100) / `total_days', "%7.1f")
local total_1_days = "`r(N)'\; `total_1_days_perc'%"

count if white == 1 & counting_days == 0
local total_1_questions_perc = string((`r(N)'*100) / `total_questions', "%7.1f")
local total_1_questions = "`r(N)'\; `total_1_questions_perc'%"

flexmat addrow, data(White,`total_1',`total_1_days',`total_1_questions',`r_chi2_star') ///
dec(3) row(4) col(1) location(`next')

// Asian
count if asian == 1
local total_1_perc = string((`r(N)' * 100) / `total_N', "%7.1f")
local total_1 = "`r(N)'\; `total_1_perc'%"

count if asian == 1 & counting_days == 1
local total_1_days_perc = string((`r(N)' * 100) / `total_days', "%7.1f")
local total_1_days = "`r(N)'\; `total_1_days_perc'%"

count if asian == 1 & counting_days == 0
local total_1_questions_perc = string((`r(N)'*100) / `total_questions', "%7.1f")
local total_1_questions = "`r(N)'\; `total_1_questions_perc'%"

flexmat addrow, data(Asian,`total_1',`total_1_days',`total_1_questions',`r_chi2_star') ///
dec(3) row(5) col(1) location(`next')

// NonWhiteOrAsian
count if nonwhiteorasian == 1
local total_1_perc = string((`r(N)' * 100) / `total_N', "%7.1f")
local total_1 = "`r(N)'\; `total_1_perc'%"

count if nonwhiteorasian == 1 & counting_days == 1
local total_1_days_perc = string((`r(N)' * 100) / `total_days', "%7.1f")
local total_1_days = "`r(N)'\; `total_1_days_perc'%"

count if nonwhiteorasian == 1 & counting_days == 0
local total_1_questions_perc = string((`r(N)'*100) / `total_questions', "%7.1f")
local total_1_questions = "`r(N)'\; `total_1_questions_perc'%"

flexmat addrow, data(Neither White or Asian,`total_1',`total_1_days',`total_1_questions',`r_chi2_star') ///
dec(3) row(6) col(1) location(`next')

// Academic Level
tabulate academiclevel counting_days, chi2
local total_N = `r(N)'
local r_chi2 = string(`r(chi2)', "%7.3f")
local r_chi2_p = ""
if `r(p)' < 0.05 local r_chi2_p = "*"
if `r(p)' < 0.01 local r_chi2_p = "**"
if `r(p)' < 0.001 local r_chi2_p = "***"
local r_chi2_star = "`r_chi2' `r_chi2_p'"

count if academiclevel == "Sophomore"
```

```
local total_1_perc = string((`r(N)' * 100) / `total_N', "%7.1f")
local total_1 = "`r(N)'\; `total_1_perc'%"

count if academiclevel == "Sophomore" & counting_days == 1
local total_1_days_perc = string((`r(N)' * 100) / `total_days', "%7.1f")
local total_1_days = "`r(N)'\; `total_1_days_perc'%"

count if academiclevel == "Sophomore" & counting_days == 0
local total_1_questions_perc = string((`r(N)'*100) / `total_questions', "%7.1f")
local total_1_questions = "`r(N)'\; `total_1_questions_perc'%"

flexmat addrow, data(Sophomore,`total_1',`total_1_days',`total_1_questions',`r_chi2_star') ///
dec(3) row(7) col(1) location(`next')

count if academiclevel == "Junior"
local total_1_perc = string((`r(N)' * 100) / `total_N', "%7.1f")
local total_1 = "`r(N)'\; `total_1_perc'%"

count if academiclevel == "Junior" & counting_days == 1
local total_1_days_perc = string((`r(N)' * 100) / `total_days', "%7.1f")
local total_1_days = "`r(N)'\; `total_1_days_perc'%"

count if academiclevel == "Junior" & counting_days == 0
local total_1_questions_perc = string((`r(N)'*100) / `total_questions', "%7.1f")
local total_1_questions = "`r(N)'\; `total_1_questions_perc'%"

flexmat addrow, data(Junior,`total_1',`total_1_days',`total_1_questions',`r_chi2_star') ///
dec(3) row(8) col(1) location(`next')

count if academiclevel == "Senior"
local total_1_perc = string((`r(N)' * 100) / `total_N', "%7.1f")
local total_1 = "`r(N)'\; `total_1_perc'%"

count if academiclevel == "Senior" & counting_days == 1
local total_1_days_perc = string((`r(N)' * 100) / `total_days', "%7.1f")
local total_1_days = "`r(N)'\; `total_1_days_perc'%"

count if academiclevel == "Senior" & counting_days == 0
local total_1_questions_perc = string((`r(N)'*100) / `total_questions', "%7.1f")
local total_1_questions = "`r(N)'\; `total_1_questions_perc'%"

flexmat addrow, data(Senior,`total_1',`total_1_days',`total_1_questions',`r_chi2_star') ///
dec(3) row(9) col(1) location(`next')

// NonNative English Speakers
tabulate nonnativeenglish counting_days, chi2
local total_N = `r(N)'
local r_chi2 = string(`r(chi2)', "%7.3f")
local r_chi2_p = ""
if `r(p)' < 0.05 local r_chi2_p = "*"
if `r(p)' < 0.01 local r_chi2_p = "**"
if `r(p)' < 0.001 local r_chi2_p = "***"
local r_chi2_star = "`r_chi2' `r_chi2_p'"

count if nonnativeenglish == 1
local total_1_perc = string((`r(N)' * 100) / `total_N', "%7.1f")
local total_1 = "`r(N)'\; `total_1_perc'%"

count if nonnativeenglish == 1 & counting_days == 1
local total_1_days_perc = string((`r(N)' * 100) / `total_days', "%7.1f")
local total_1_days = "`r(N)'\; `total_1_days_perc'%"

count if nonnativeenglish == 1 & counting_days == 0
local total_1_questions_perc = string((`r(N)'*100) / `total_questions', "%7.1f")
local total_1_questions = "`r(N)'\; `total_1_questions_perc'%"

flexmat addrow, data(NonNative English Speakers,`total_1',`total_1_days',`total_1_questions',`r_chi2_star') ///
dec(3) row(10) col(1) location(`next')


/***
*** Table 6
***/
// Comparing the outcomes across the two conditions using two-sided t-test and
// Wilcoxon rank sum test with continuity correction.

```

```
// Create a new variable as the reverse of the existing dichotomous variable.
gen counting_days_r = 1 - counting_days

* Increment the location to store a new table
loc ++next

flexmat addparts, title(Table E4) location(`next')
flexmat addcell, data(t-test) row(1) col(2) location(`next')
flexmat addcell, data(Wilcoxon rank-sum) row(1) col(3) location(`next')

ttest final, by(counting_days_r)

local r_t = string(`r(t)', "%7.3f")
local r_t_p = ""
if `r(p_l)' < 0.05 local r_t_p = "*"
if `r(p_l)' < 0.01 local r_t_p = "**"
if `r(p_l)' < 0.001 local r_t_p = "***"
local r_t_p_star = "`r_t'`r_t_p'"

ranksum final, by(counting_days_r) exact

local r_z = string(`r(z)', "%7.3f")
local r_z_p = ""
if `r(p_l)' < 0.05 local r_z_p = "*"
if `r(p_l)' < 0.01 local r_z_p = "**"
if `r(p_l)' < 0.001 local r_z_p = "***"
local r_z_p_star = "`r_z'`r_z_p'"

flexmat addrow, data(Final Exam Score,`r_t_p_star',`r_z_p_star') ///
dec(3) row(2) col(1) location(`next')

// Practice Days
ttest practice_days, by(counting_days_r)

local r_t = string(`r(t)', "%7.3f")
local r_t_p = ""
if `r(p_l)' < 0.05 local r_t_p = "*"
if `r(p_l)' < 0.01 local r_t_p = "**"
if `r(p_l)' < 0.001 local r_t_p = "***"
local r_t_p_star = "`r_t'`r_t_p'"

ranksum practice_days, by(counting_days_r) exact

local r_z = string(`r(z)', "%7.3f")
local r_z_p = ""
if `r(p_l)' < 0.05 local r_z_p = "*"
if `r(p_l)' < 0.01 local r_z_p = "**"
if `r(p_l)' < 0.001 local r_z_p = "***"
local r_z_p_star = "`r_z'`r_z_p'"

flexmat addrow, data(Practice Days,`r_t_p_star',`r_z_p_star') ///
dec(3) row(3) col(1) location(`next')

// Practiced Questions
ttest practice_count, by(counting_days_r)

local r_t = string(`r(t)', "%7.3f")
local r_t_p = ""
if `r(p_l)' < 0.05 local r_t_p = "*"
if `r(p_l)' < 0.01 local r_t_p = "**"
if `r(p_l)' < 0.001 local r_t_p = "***"
local r_t_p_star = "`r_t'`r_t_p'"

ranksum practice_count, by(counting_days_r) exact

local r_z = string(`r(z)', "%7.3f")
local r_z_p = ""
if `r(p_l)' < 0.05 local r_z_p = "*"
if `r(p_l)' < 0.01 local r_z_p = "**"
if `r(p_l)' < 0.001 local r_z_p = "***"
local r_z_p_star = "`r_z'`r_z_p'"

flexmat addrow, data(Practiced Questions,`r_t_p_star',`r_z_p_star') ///
dec(3) row(4) col(1) location(`next')

asdocx export, save(Tables.tex)
```

```
/***
*** Tables 7, D2,
***/

// Regression Results
// Table D2 - Column 1
betareg final_0_1 i.counting_days c.gpa c.gpa#i.counting_days

local r_1_title = e(title)
local r_1_link_function = e(linkf)
local r_1_slink_function = e(linkt)
local r_1_log_likelihood = string(e(ll), "%7.3f")
local r_1_chi2 = string(e(chi2), "%7.3f")
local r_1_p = ""
if e(p) < 0.05 local r_1_p = "*"
if e(p) < 0.01 local r_1_p = "**"
if e(p) < 0.001 local r_1_p = "***"
local r_1_n = e(N)

local r_1_chi2_p_star = "\begin{tabular}[c]{@{}l@{}}`r_1_chi2'\\ `r_1_p'\end{tabular}"

local r_1_counting_days = string(r(table)[1,2], "%7.3f")
local r_1_counting_days_se = string(r(table)[2,2], "%7.3f")
local r_1_counting_days_p = ""
if r(table)[4,2] < 0.05 local r_1_counting_days_p = "*"
if r(table)[4,2] < 0.01 local r_1_counting_days_p = "**"
if r(table)[4,2] < 0.001 local r_1_counting_days_p = "***"

local r_1_counting_days_p_star = "\begin{tabular}[c]{@{}l@{}}`r_1_counting_days'`r_1_counting_days_p'\\ (`r_1_counting_days_se')\end{tabular}"

local r_1_gpa = string(r(table)[1,3], "%7.3f")
local r_1_gpa_se = string(r(table)[2,3], "%7.3f")
local r_1_gpa_p = ""
if r(table)[4,3] < 0.05 local r_1_gpa_p = "*"
if r(table)[4,3] < 0.01 local r_1_gpa_p = "**"
if r(table)[4,3] < 0.001 local r_1_gpa_p = "***"

local r_1_gpa_p_star = "\begin{tabular}[c]{@{}l@{}}`r_1_gpa'`r_1_gpa_p'\\ (`r_1_gpa_se')\end{tabular}"

local r_1_counting_days_gpa = string(r(table)[1,5], "%7.3f")
local r_1_counting_days_gpa_se = string(r(table)[2,5], "%7.3f")
local r_1_counting_days_gpa_p = ""
if r(table)[4,5] < 0.05 local r_1_counting_days_gpa_p = "*"
if r(table)[4,5] < 0.01 local r_1_counting_days_gpa_p = "**"
if r(table)[4,5] < 0.001 local r_1_counting_days_gpa_p = "***"

local r_1_counting_days_gpa_p_star = "\begin{tabular}[c]{@{}l@{}}`r_1_counting_days_gpa'`r_1_counting_days_gpa_p'\\ (`r_1_counting_days_gp↩
a_se')\end{tabular}"

local r_1_const = string(r(table)[1,6], "%7.3f")
local r_1_const_se = string(r(table)[2,6], "%7.3f")
local r_1_const_p = ""
if r(table)[4,6] < 0.05 local r_1_const_p = "*"
if r(table)[4,6] < 0.01 local r_1_const_p = "**"
if r(table)[4,6] < 0.001 local r_1_const_p = "***"

local r_1_const_p_star = "\begin{tabular}[c]{@{}l@{}}`r_1_const'`r_1_const_p'\\ (`r_1_const_se')\end{tabular}"

local r_1_scale = string(r(table)[1,7], "%7.3f")
local r_1_scale_se = string(r(table)[2,7], "%7.3f")
local r_1_scale_p = ""
if r(table)[4,7] < 0.05 local r_1_scale_p = "*"
if r(table)[4,7] < 0.01 local r_1_scale_p = "**"
if r(table)[4,7] < 0.001 local r_1_scale_p = "***"

local r_1_scale_p_star = "\begin{tabular}[c]{@{}l@{}}Scale:\\ `r_1_scale'`r_1_scale_p'\\ (`r_1_scale_se')\end{tabular}"

local r_1_scale_p_star = "\begin{tabular}[c]{@{}l@{}}Scale:\\ `r_1_scale'`r_1_scale_p'\\ (`r_1_scale_se')\end{tabular}"

// Table 7 - Column 1
betareg final_0_1 i.counting_days c.gpa c.gpa#i.counting_days ///
1.female 1.junior 1.senior 1.asian 1.nonwhiteorasian 1.nonnativeenglish

local r_2_title = e(title)
```

```
local r_2_link_function = e(linkf)
local r_2_slink_function = e(linkt)
local r_2_log_likelihood = string(e(ll), "%7.3f")
local r_2_chi2 = string(e(chi2), "%7.3f")
local r_2_p = ""
if e(p) < 0.05 local r_2_p = "*"
if e(p) < 0.01 local r_2_p = "**"
if e(p) < 0.001 local r_2_p = "***"
local r_2_n = e(N)

local r_2_chi2_p_star = "\begin{tabular}[c]{@{}l@{}}`r_2_chi2'\\ `r_2_p'\end{tabular}"

local r_2_counting_days = string(r(table)[1,2], "%7.3f")
local r_2_counting_days_se = string(r(table)[2,2], "%7.3f")
local r_2_counting_days_p = ""
if r(table)[4,2] < 0.05 local r_2_counting_days_p = "*"
if r(table)[4,2] < 0.01 local r_2_counting_days_p = "**"
if r(table)[4,2] < 0.001 local r_2_counting_days_p = "***"

local r_2_counting_days_p_star = "\begin{tabular}[c]{@{}l@{}}`r_2_counting_days'`r_2_counting_days_p'\\ (`r_2_counting_days_se')\end{tabular}"

local r_2_gpa = string(r(table)[1,3], "%7.3f")
local r_2_gpa_se = string(r(table)[2,3], "%7.3f")
local r_2_gpa_p = ""
if r(table)[4,3] < 0.05 local r_2_gpa_p = "*"
if r(table)[4,3] < 0.01 local r_2_gpa_p = "**"
if r(table)[4,3] < 0.001 local r_2_gpa_p = "***"

local r_2_gpa_p_star = "\begin{tabular}[c]{@{}l@{}}`r_2_gpa'`r_2_gpa_p'\\ (`r_2_gpa_se')\end{tabular}"

local r_2_counting_days_gpa = string(r(table)[1,5], "%7.3f")
local r_2_counting_days_gpa_se = string(r(table)[2,5], "%7.3f")
local r_2_counting_days_gpa_p = ""
if r(table)[4,5] < 0.05 local r_2_counting_days_gpa_p = "*"
if r(table)[4,5] < 0.01 local r_2_counting_days_gpa_p = "**"
if r(table)[4,5] < 0.001 local r_2_counting_days_gpa_p = "***"

local r_2_counting_days_gpa_p_star = "\begin{tabular}[c]{@{}l@{}}`r_2_counting_days_gpa'`r_2_counting_days_gpa_p'\\ (`r_2_counting_days_gpa_se')\end{tabular}"

local r_2_female = string(r(table)[1,6], "%7.3f")
local r_2_female_se = string(r(table)[2,6], "%7.3f")
local r_2_female_p = ""
if r(table)[4,6] < 0.05 local r_2_female_p = "*"
if r(table)[4,6] < 0.01 local r_2_female_p = "**"
if r(table)[4,6] < 0.001 local r_2_female_p = "***"

local r_2_female_p_star = "\begin{tabular}[c]{@{}l@{}}`r_2_female'`r_2_female_p'\\ (`r_2_female_se')\end{tabular}"

local r_2_junior = string(r(table)[1,7], "%7.3f")
local r_2_junior_se = string(r(table)[2,7], "%7.3f")
local r_2_junior_p = ""
if r(table)[4,7] < 0.05 local r_2_junior_p = "*"
if r(table)[4,7] < 0.01 local r_2_junior_p = "**"
if r(table)[4,7] < 0.001 local r_2_junior_p = "***"

local r_2_junior_p_star = "\begin{tabular}[c]{@{}l@{}}`r_2_junior'`r_2_junior_p'\\ (`r_2_junior_se')\end{tabular}"

local r_2_senior = string(r(table)[1,8], "%7.3f")
local r_2_senior_se = string(r(table)[2,8], "%7.3f")
local r_2_senior_p = ""
if r(table)[4,8] < 0.05 local r_2_senior_p = "*"
if r(table)[4,8] < 0.01 local r_2_senior_p = "**"
if r(table)[4,8] < 0.001 local r_2_senior_p = "***"

local r_2_senior_p_star = "\begin{tabular}[c]{@{}l@{}}`r_2_senior'`r_2_senior_p'\\ (`r_2_senior_se')\end{tabular}"

local r_2_asian = string(r(table)[1,9], "%7.3f")
local r_2_asian_se = string(r(table)[2,9], "%7.3f")
local r_2_asian_p = ""
if r(table)[4,9] < 0.05 local r_2_asian_p = "*"
if r(table)[4,9] < 0.01 local r_2_asian_p = "**"
if r(table)[4,9] < 0.001 local r_2_asian_p = "***"

local r_2_asian_p_star = "\begin{tabular}[c]{@{}l@{}}`r_2_asian'`r_2_asian_p'\\ (`r_2_asian_se')\end{tabular}"
```

```
local r_2_nonwhiteorasian = string(r(table)[1,10], "%7.3f")
local r_2_nonwhiteorasian_se = string(r(table)[2,10], "%7.3f")
local r_2_nonwhiteorasian_p = ""
if r(table)[4,10] < 0.05 local r_2_nonwhiteorasian_p = "*"
if r(table)[4,10] < 0.01 local r_2_nonwhiteorasian_p = "**"
if r(table)[4,8] < 0.001 local r_2_nonwhiteorasian_p = "***"

local r_2_nonwhiteorasian_p_star = "\begin{tabular}[c]{@{}l@{}}`r_2_nonwhiteorasian'`r_2_nonwhiteorasian_p'\\ (`r_2_nonwhiteorasian_se')\end{tabul

local r_2_nonnativeenglish = string(r(table)[1,11], "%7.3f")
local r_2_nonnativeenglish_se = string(r(table)[2,11], "%7.3f")
local r_2_nonnativeenglish_p = ""
if r(table)[4,11] < 0.05 local r_2_nonnativeenglish_p = "*"
if r(table)[4,11] < 0.01 local r_2_nonnativeenglish_p = "**"
if r(table)[4,11] < 0.001 local r_2_nonnativeenglish_p = "***"

local r_2_nonnativeenglish_p_star = "\begin{tabular}[c]{@{}l@{}}`r_2_nonnativeenglish'`r_2_nonnativeenglish_p'\\ (`r_2_nonnativeenglish_se')\end{tabular}"

local r_2_const = string(r(table)[1,12], "%7.3f")
local r_2_const_se = string(r(table)[2,12], "%7.3f")
local r_2_const_p = ""
if r(table)[4,12] < 0.05 local r_2_const_p = "*"
if r(table)[4,12] < 0.01 local r_2_const_p = "**"
if r(table)[4,12] < 0.001 local r_2_const_p = "***"

local r_2_const_p_star = "\begin{tabular}[c]{@{}l@{}}`r_2_const'`r_2_const_p'\\ (`r_2_const_se')\end{tabular}"

local r_2_scale = string(r(table)[1,13], "%7.3f")
local r_2_scale_se = string(r(table)[2,13], "%7.3f")
local r_2_scale_p = ""
if r(table)[4,13] < 0.05 local r_2_scale_p = "*"
if r(table)[4,13] < 0.01 local r_2_scale_p = "**"
if r(table)[4,13] < 0.001 local r_2_scale_p = "***"

local r_2_scale_p_star = "\begin{tabular}[c]{@{}l@{}}Scale:\\ `r_2_scale'`r_2_scale_p'\\ (`r_2_scale_se')\end{tabular}"

// Table D2 - Column 2
nbreg practice_days i.counting_days c.gpa c.gpa#i.counting_days

local r_3_title = e(title)
local r_3_log_likelihood = string(e(ll), "%7.3f")
local r_3_chi2 = string(e(chi2), "%7.3f")
local r_3_p = ""
if e(p) < 0.05 local r_3_p = "*"
if e(p) < 0.01 local r_3_p = "**"
if e(p) < 0.001 local r_3_p = "***"
local r_3_n = e(N)

local r_3_chi2_p_star = "\begin{tabular}[c]{@{}l@{}}`r_3_chi2'\\ `r_3_p'\end{tabular}"

local r_3_counting_days = string(r(table)[1,2], "%7.3f")
local r_3_counting_days_se = string(r(table)[2,2], "%7.3f")
local r_3_counting_days_p = ""
if r(table)[4,2] < 0.05 local r_3_counting_days_p = "*"
if r(table)[4,2] < 0.01 local r_3_counting_days_p = "**"
if r(table)[4,2] < 0.001 local r_3_counting_days_p = "***"

local r_3_counting_days_p_star = "\begin{tabular}[c]{@{}l@{}}`r_3_counting_days'`r_3_counting_days_p'\\ (`r_3_counting_days_se')\end{tabular}"

local r_3_gpa = string(r(table)[1,3], "%7.3f")
local r_3_gpa_se = string(r(table)[2,3], "%7.3f")
local r_3_gpa_p = ""
if r(table)[4,3] < 0.05 local r_3_gpa_p = "*"
if r(table)[4,3] < 0.01 local r_3_gpa_p = "**"
if r(table)[4,3] < 0.001 local r_3_gpa_p = "***"

local r_3_gpa_p_star = "\begin{tabular}[c]{@{}l@{}}`r_3_gpa'`r_3_gpa_p'\\ (`r_3_gpa_se')\end{tabular}"

local r_3_counting_days_gpa = string(r(table)[1,5], "%7.3f")
local r_3_counting_days_gpa_se = string(r(table)[2,5], "%7.3f")
local r_3_counting_days_gpa_p = ""
if r(table)[4,5] < 0.05 local r_3_counting_days_gpa_p = "*"
if r(table)[4,5] < 0.01 local r_3_counting_days_gpa_p = "**"
if r(table)[4,5] < 0.001 local r_3_counting_days_gpa_p = "***"
```

```
local r_3_counting_days_gpa_p_star = "\begin{tabular}[c]{@{}l@{}}`r_3_counting_days_gpa'`r_3_counting_days_gpa_p'\\ (`r_3_counting_days_gpa_se')\end{tabular}"

local r_3_const = string(r(table)[1,6], "%7.3f")
local r_3_const_se = string(r(table)[2,6], "%7.3f")
local r_3_const_p = ""
if r(table)[4,6] < 0.05 local r_3_const_p = "*"
if r(table)[4,6] < 0.01 local r_3_const_p = "**"
if r(table)[4,6] < 0.001 local r_3_const_p = "***"

local r_3_const_p_star = "\begin{tabular}[c]{@{}l@{}}`r_3_const'`r_3_const_p'\\ (`r_3_const_se')\end{tabular}"

local r_3_scale = string(r(table)[1,8], "%7.3f")
local r_3_scale_se = string(r(table)[2,8], "%7.3f")
local r_3_scale_p = "***"

local r_3_scale_p_star = "\begin{tabular}[c]{@{}l@{}}Alpha:\\ `r_3_scale'`r_3_scale_p'\\ (`r_3_scale_se')\end{tabular}"

// Table 7 - Column 2
nbreg practice_days i.counting_days c.gpa c.gpa#i.counting_days ///
1.female 1.junior 1.senior 1.asian 1.nonwhiteorasian 1.nonnativeenglish

local r_4_title = e(title)
local r_4_log_likelihood = string(e(ll), "%7.3f")
local r_4_chi2 = string(e(chi2), "%7.3f")
local r_4_p = ""
if e(p) < 0.05 local r_4_p = "*"
if e(p) < 0.01 local r_4_p = "**"
if e(p) < 0.001 local r_4_p = "***"
local r_4_n = e(N)

local r_4_chi2_p_star = "\begin{tabular}[c]{@{}l@{}}`r_4_chi2'\\ `r_4_p'\end{tabular}"

local r_4_counting_days = string(r(table)[1,2], "%7.3f")
local r_4_counting_days_se = string(r(table)[2,2], "%7.3f")
local r_4_counting_days_p = ""
if r(table)[4,2] < 0.05 local r_4_counting_days_p = "*"
if r(table)[4,2] < 0.01 local r_4_counting_days_p = "**"
if r(table)[4,2] < 0.001 local r_4_counting_days_p = "***"

local r_4_counting_days_p_star = "\begin{tabular}[c]{@{}l@{}}`r_4_counting_days'`r_4_counting_days_p'\\ (`r_4_counting_days_se')\end{tabular}"

local r_4_gpa = string(r(table)[1,3], "%7.3f")
local r_4_gpa_se = string(r(table)[2,3], "%7.3f")
local r_4_gpa_p = ""
if r(table)[4,3] < 0.05 local r_4_gpa_p = "*"
if r(table)[4,3] < 0.01 local r_4_gpa_p = "**"
if r(table)[4,3] < 0.001 local r_4_gpa_p = "***"

local r_4_gpa_p_star = "\begin{tabular}[c]{@{}l@{}}`r_4_gpa'`r_4_gpa_p'\\ (`r_4_gpa_se')\end{tabular}"

local r_4_counting_days_gpa = string(r(table)[1,5], "%7.3f")
local r_4_counting_days_gpa_se = string(r(table)[2,5], "%7.3f")
local r_4_counting_days_gpa_p = ""
if r(table)[4,5] < 0.05 local r_4_counting_days_gpa_p = "*"
if r(table)[4,5] < 0.01 local r_4_counting_days_gpa_p = "**"
if r(table)[4,5] < 0.001 local r_4_counting_days_gpa_p = "***"

local r_4_counting_days_gpa_p_star = "\begin{tabular}[c]{@{}l@{}}`r_4_counting_days_gpa'`r_4_counting_days_gpa_p'\\ (`r_4_counting_days_gpa_se')\end{tabular}"

local r_4_female = string(r(table)[1,6], "%7.3f")
local r_4_female_se = string(r(table)[2,6], "%7.3f")
local r_4_female_p = ""
if r(table)[4,6] < 0.05 local r_4_female_p = "*"
if r(table)[4,6] < 0.01 local r_4_female_p = "**"
if r(table)[4,6] < 0.001 local r_4_female_p = "***"

local r_4_female_p_star = "\begin{tabular}[c]{@{}l@{}}`r_4_female'`r_4_female_p'\\ (`r_4_female_se')\end{tabular}"

local r_4_junior = string(r(table)[1,7], "%7.3f")
local r_4_junior_se = string(r(table)[2,7], "%7.3f")
local r_4_junior_p = ""
if r(table)[4,7] < 0.05 local r_4_junior_p = "*"
if r(table)[4,7] < 0.01 local r_4_junior_p = "**"
if r(table)[4,7] < 0.001 local r_4_junior_p = "***"
```

```
local r_4_junior_p_star = "\begin{tabular}[c]{@{}l@{}}`r_4_junior'`r_4_junior_p'\\ (`r_4_junior_se')\end{tabular}"

local r_4_senior = string(r(table)[1,8], "%7.3f")
local r_4_senior_se = string(r(table)[2,8], "%7.3f")
local r_4_senior_p = ""
if r(table)[4,8] < 0.05 local r_4_senior_p = "*"
if r(table)[4,8] < 0.01 local r_4_senior_p = "**"
if r(table)[4,8] < 0.001 local r_4_senior_p = "***"

local r_4_senior_p_star = "\begin{tabular}[c]{@{}l@{}}`r_4_senior'`r_4_senior_p'\\ (`r_4_senior_se')\end{tabular}"

local r_4_asian = string(r(table)[1,9], "%7.3f")
local r_4_asian_se = string(r(table)[2,9], "%7.3f")
local r_4_asian_p = ""
if r(table)[4,9] < 0.05 local r_4_asian_p = "*"
if r(table)[4,9] < 0.01 local r_4_asian_p = "**"
if r(table)[4,9] < 0.001 local r_4_asian_p = "***"

local r_4_asian_p_star = "\begin{tabular}[c]{@{}l@{}}`r_4_asian'`r_4_asian_p'\\ (`r_4_asian_se')\end{tabular}"

local r_4_nonwhiteorasian = string(r(table)[1,10], "%7.3f")
local r_4_nonwhiteorasian_se = string(r(table)[2,10], "%7.3f")
local r_4_nonwhiteorasian_p = ""
if r(table)[4,10] < 0.05 local r_4_nonwhiteorasian_p = "*"
if r(table)[4,10] < 0.01 local r_4_nonwhiteorasian_p = "**"
if r(table)[4,8] < 0.001 local r_4_nonwhiteorasian_p = "***"

local r_4_nonwhiteorasian_p_star = "\begin{tabular}[c]{@{}l@{}}`r_4_nonwhiteorasian'`r_4_nonwhiteorasian_p'\\ (`r_4_nonwhiteorasian_se')\end{tabul

local r_4_nonnativeenglish = string(r(table)[1,11], "%7.3f")
local r_4_nonnativeenglish_se = string(r(table)[2,11], "%7.3f")
local r_4_nonnativeenglish_p = ""
if r(table)[4,11] < 0.05 local r_4_nonnativeenglish_p = "*"
if r(table)[4,11] < 0.01 local r_4_nonnativeenglish_p = "**"
if r(table)[4,11] < 0.001 local r_4_nonnativeenglish_p = "***"

local r_4_nonnativeenglish_p_star = "\begin{tabular}[c]{@{}l@{}}`r_4_nonnativeenglish'`r_4_nonnativeenglish_p'\\ (`r_4_nonnativeenglish_se')\end{tabular}"

local r_4_const = string(r(table)[1,12], "%7.3f")
local r_4_const_se = string(r(table)[2,12], "%7.3f")
local r_4_const_p = ""
if r(table)[4,12] < 0.05 local r_4_const_p = "*"
if r(table)[4,12] < 0.01 local r_4_const_p = "**"
if r(table)[4,12] < 0.001 local r_4_const_p = "***"

local r_4_const_p_star = "\begin{tabular}[c]{@{}l@{}}`r_4_const'`r_4_const_p'\\ (`r_4_const_se')\end{tabular}"

local r_4_scale = string(r(table)[1,14], "%7.3f")
local r_4_scale_se = string(r(table)[2,14], "%7.3f")
local r_4_scale_p = "***"

local r_4_scale_p_star = "\begin{tabular}[c]{@{}l@{}}Alpha:\\ `r_4_scale'`r_4_scale_p'\\ (`r_4_scale_se')\end{tabular}"

// Table D2 - Column 3
nbreg practice_count i.counting_days c.gpa c.gpa#i.counting_days

local r_5_title = e(title)
local r_5_log_likelihood = string(e(ll), "%7.3f")
local r_5_chi2 = string(e(chi2), "%7.3f")
local r_5_p = ""
if e(p) < 0.05 local r_5_p = "*"
if e(p) < 0.01 local r_5_p = "**"
if e(p) < 0.001 local r_5_p = "***"
local r_5_n = e(N)

local r_5_chi2_p_star = "\begin{tabular}[c]{@{}l@{}}`r_5_chi2'\\ `r_5_p'\end{tabular}"

local r_5_counting_days = string(r(table)[1,2], "%7.3f")
local r_5_counting_days_se = string(r(table)[2,2], "%7.3f")
local r_5_counting_days_p = ""
if r(table)[4,2] < 0.05 local r_5_counting_days_p = "*"
if r(table)[4,2] < 0.01 local r_5_counting_days_p = "**"
if r(table)[4,2] < 0.001 local r_5_counting_days_p = "***"
```

```
local r_5_counting_days_p_star = "\begin{tabular}[c]{@{}l@{}}`r_5_counting_days'`r_5_counting_days_p'\\ (`r_5_counting_days_se')\end{tabular}"

local r_5_gpa = string(r(table)[1,3], "%7.3f")
local r_5_gpa_se = string(r(table)[2,3], "%7.3f")
local r_5_gpa_p = ""
if r(table)[4,3] < 0.05 local r_5_gpa_p = "*"
if r(table)[4,3] < 0.01 local r_5_gpa_p = "**"
if r(table)[4,3] < 0.001 local r_5_gpa_p = "***"

local r_5_gpa_p_star = "\begin{tabular}[c]{@{}l@{}}`r_5_gpa'`r_5_gpa_p'\\ (`r_5_gpa_se')\end{tabular}"

local r_5_counting_days_gpa = string(r(table)[1,5], "%7.3f")
local r_5_counting_days_gpa_se = string(r(table)[2,5], "%7.3f")
local r_5_counting_days_gpa_p = ""
if r(table)[4,5] < 0.05 local r_5_counting_days_gpa_p = "*"
if r(table)[4,5] < 0.01 local r_5_counting_days_gpa_p = "**"
if r(table)[4,5] < 0.001 local r_5_counting_days_gpa_p = "***"

local r_5_counting_days_gpa_p_star = "\begin{tabular}[c]{@{}l@{}}`r_5_counting_days_gpa'`r_5_counting_days_gpa_p'\\ (`r_5_counting_days_gp↩
a_se')\end{tabular}"

local r_5_const = string(r(table)[1,6], "%7.3f")
local r_5_const_se = string(r(table)[2,6], "%7.3f")
local r_5_const_p = ""
if r(table)[4,6] < 0.05 local r_5_const_p = "*"
if r(table)[4,6] < 0.01 local r_5_const_p = "**"
if r(table)[4,6] < 0.001 local r_5_const_p = "***"

local r_5_const_p_star = "\begin{tabular}[c]{@{}l@{}}`r_5_const'`r_5_const_p'\\ (`r_5_const_se')\end{tabular}"

local r_5_scale = string(r(table)[1,8], "%7.3f")
local r_5_scale_se = string(r(table)[2,8], "%7.3f")
local r_5_scale_p = "***"

local r_5_scale_p_star = "\begin{tabular}[c]{@{}l@{}}Alpha:\\ `r_5_scale'`r_5_scale_p'\\ (`r_5_scale_se')\end{tabular}"

// Table 7 - Column 3
nbreg practice_count i.counting_days c.gpa c.gpa#i.counting_days ///
1.female 1.junior 1.senior 1.asian 1.nonwhiteorasian 1.nonnativeenglish

local r_6_title = e(title)
local r_6_log_likelihood = string(e(ll), "%7.3f")
local r_6_chi2 = string(e(chi2), "%7.3f")
local r_6_p = ""
if e(p) < 0.05 local r_6_p = "*"
if e(p) < 0.01 local r_6_p = "**"
if e(p) < 0.001 local r_6_p = "***"
local r_6_n = e(N)

local r_6_chi2_p_star = "\begin{tabular}[c]{@{}l@{}}`r_6_chi2'\\ `r_6_p'\end{tabular}"

local r_6_counting_days = string(r(table)[1,2], "%7.3f")
local r_6_counting_days_se = string(r(table)[2,2], "%7.3f")
local r_6_counting_days_p = ""
if r(table)[4,2] < 0.05 local r_6_counting_days_p = "*"
if r(table)[4,2] < 0.01 local r_6_counting_days_p = "**"
if r(table)[4,2] < 0.001 local r_6_counting_days_p = "***"

local r_6_counting_days_p_star = "\begin{tabular}[c]{@{}l@{}}`r_6_counting_days'`r_6_counting_days_p'\\ (`r_6_counting_days_se')\end{tabular}"

local r_6_gpa = string(r(table)[1,3], "%7.3f")
local r_6_gpa_se = string(r(table)[2,3], "%7.3f")
local r_6_gpa_p = ""
if r(table)[4,3] < 0.05 local r_6_gpa_p = "*"
if r(table)[4,3] < 0.01 local r_6_gpa_p = "**"
if r(table)[4,3] < 0.001 local r_6_gpa_p = "***"

local r_6_gpa_p_star = "\begin{tabular}[c]{@{}l@{}}`r_6_gpa'`r_6_gpa_p'\\ (`r_6_gpa_se')\end{tabular}"

local r_6_counting_days_gpa = string(r(table)[1,5], "%7.3f")
local r_6_counting_days_gpa_se = string(r(table)[2,5], "%7.3f")
local r_6_counting_days_gpa_p = ""
if r(table)[4,5] < 0.05 local r_6_counting_days_gpa_p = "*"
if r(table)[4,5] < 0.01 local r_6_counting_days_gpa_p = "**"
if r(table)[4,5] < 0.001 local r_6_counting_days_gpa_p = "***"

```

```
local r_6_counting_days_gpa_p_star = "\begin{tabular}[c]{@{}l@{}}`r_6_counting_days_gpa'`r_6_counting_days_gpa_p'\\ (`r_6_counting_days_gpa_se')\end{tabular}"

local r_6_female = string(r(table)[1,6], "%7.3f")
local r_6_female_se = string(r(table)[2,6], "%7.3f")
local r_6_female_p = ""
if r(table)[4,6] < 0.05 local r_6_female_p = "*"
if r(table)[4,6] < 0.01 local r_6_female_p = "**"
if r(table)[4,6] < 0.001 local r_6_female_p = "***"

local r_6_female_p_star = "\begin{tabular}[c]{@{}l@{}}`r_6_female'`r_6_female_p'\\ (`r_6_female_se')\end{tabular}"

local r_6_junior = string(r(table)[1,7], "%7.3f")
local r_6_junior_se = string(r(table)[2,7], "%7.3f")
local r_6_junior_p = ""
if r(table)[4,7] < 0.05 local r_6_junior_p = "*"
if r(table)[4,7] < 0.01 local r_6_junior_p = "**"
if r(table)[4,7] < 0.001 local r_6_junior_p = "***"

local r_6_junior_p_star = "\begin{tabular}[c]{@{}l@{}}`r_6_junior'`r_6_junior_p'\\ (`r_6_junior_se')\end{tabular}"

local r_6_senior = string(r(table)[1,8], "%7.3f")
local r_6_senior_se = string(r(table)[2,8], "%7.3f")
local r_6_senior_p = ""
if r(table)[4,8] < 0.05 local r_6_senior_p = "*"
if r(table)[4,8] < 0.01 local r_6_senior_p = "**"
if r(table)[4,8] < 0.001 local r_6_senior_p = "***"

local r_6_senior_p_star = "\begin{tabular}[c]{@{}l@{}}`r_6_senior'`r_6_senior_p'\\ (`r_6_senior_se')\end{tabular}"

local r_6_asian = string(r(table)[1,9], "%7.3f")
local r_6_asian_se = string(r(table)[2,9], "%7.3f")
local r_6_asian_p = ""
if r(table)[4,9] < 0.05 local r_6_asian_p = "*"
if r(table)[4,9] < 0.01 local r_6_asian_p = "**"
if r(table)[4,9] < 0.001 local r_6_asian_p = "***"

local r_6_asian_p_star = "\begin{tabular}[c]{@{}l@{}}`r_6_asian'`r_6_asian_p'\\ (`r_6_asian_se')\end{tabular}"

local r_6_nonwhiteorasian = string(r(table)[1,10], "%7.3f")
local r_6_nonwhiteorasian_se = string(r(table)[2,10], "%7.3f")
local r_6_nonwhiteorasian_p = ""
if r(table)[4,10] < 0.05 local r_6_nonwhiteorasian_p = "*"
if r(table)[4,10] < 0.01 local r_6_nonwhiteorasian_p = "**"
if r(table)[4,10] < 0.001 local r_6_nonwhiteorasian_p = "***"

local r_6_nonwhiteorasian_p_star = "\begin{tabular}[c]{@{}l@{}}`r_6_nonwhiteorasian'`r_6_nonwhiteorasian_p'\\ (`r_6_nonwhiteorasian_se')\end{tabul

local r_6_nonnativeenglish = string(r(table)[1,11], "%7.3f")
local r_6_nonnativeenglish_se = string(r(table)[2,11], "%7.3f")
local r_6_nonnativeenglish_p = ""
if r(table)[4,11] < 0.05 local r_6_nonnativeenglish_p = "*"
if r(table)[4,11] < 0.01 local r_6_nonnativeenglish_p = "**"
if r(table)[4,11] < 0.001 local r_6_nonnativeenglish_p = "***"

local r_6_nonnativeenglish_p_star = "\begin{tabular}[c]{@{}l@{}}`r_6_nonnativeenglish'`r_6_nonnativeenglish_p'\\ (`r_6_nonnativeenglish_se')\end{tabular}"

local r_6_const = string(r(table)[1,12], "%7.3f")
local r_6_const_se = string(r(table)[2,12], "%7.3f")
local r_6_const_p = ""
if r(table)[4,12] < 0.05 local r_6_const_p = "*"
if r(table)[4,12] < 0.01 local r_6_const_p = "**"
if r(table)[4,12] < 0.001 local r_6_const_p = "***"

local r_6_const_p_star = "\begin{tabular}[c]{@{}l@{}}`r_6_const'`r_6_const_p'\\ (`r_6_const_se')\end{tabular}"

local r_6_scale = string(r(table)[1,14], "%7.3f")
local r_6_scale_se = string(r(table)[2,14], "%7.3f")
local r_6_scale_p = "***"

local r_6_scale_p_star = "\begin{tabular}[c]{@{}l@{}}Alpha:\\ `r_6_scale'`r_6_scale_p'\\ (`r_6_scale_se')\end{tabular}"

* Increment the location to store a new table
loc ++next
```

```
flexmat addparts, title(Tables 7, D2) location(`next')
flexmat addcell, data(\multicolumn{2}{c}{\textbf{\begin{tabular}[c]{@{}c@{}}Final Exam\\ Score\end{tabular}}}) row(1) col(1) location(`next')
flexmat addcell, data(\multicolumn{2}{c}{\textbf{Practice Days}}) row(1) col(4) location(`next')
flexmat addcell, data(\multicolumn{2}{c}{\textbf{Practice Time}}) row(1) col(6) location(`next')
flexmat addcell, data(\multicolumn{1}{l}{\begin{tabular}[l]{@{}l@{}}Beta\\ W/O\\ Ctrls\\ (1)\end{tabular}}) row(2) col(2) location(`next')
flexmat addcell, data(\multicolumn{1}{l}{\begin{tabular}[l]{@{}l@{}}Beta\\ With\\ Ctrls\\ (2)\end{tabular}}) row(2) col(3) location(`next')
flexmat addcell, data(\multicolumn{1}{l}{\begin{tabular}[l]{@{}l@{}}N. Bin.\\ W/O\\ Ctrls\\ (3)\end{tabular}}) row(2) col(4) location(`next')
flexmat addcell, data(\multicolumn{1}{l}{\begin{tabular}[l]{@{}l@{}}N. Bin.\\ With\\ Ctrls\\ (4)\end{tabular}}) row(2) col(5) location(`next')
flexmat addcell, data(\multicolumn{1}{l}{\begin{tabular}[l]{@{}l@{}}N. Bin.\\ W/O\\ Ctrls\\ (5)\end{tabular}}) row(2) col(6) location(`next')
flexmat addcell, data(\multicolumn{1}{l}{\begin{tabular}[l]{@{}l@{}}N. Bin.\\ With\\ Ctrls\\ (6)\end{tabular}}) row(2) col(7) location(`next')

flexmat addrow, data(\begin{tabular}[c]{@{}l@{}}Counting Days\\ (vs. Questions)\end{tabular},`r_1_counting_days_p_star',`r_2_counting_days_p_↩
star',`r_3_counting_days_p_star',`r_4_counting_days_p_star',`r_5_counting_days_p_star',`r_6_counting_days_p_star') ///
dec(3) row(3) col(1) location(`next')

flexmat addrow, data(GPA,`r_1_gpa_p_star',`r_2_gpa_p_star',`r_3_gpa_p_star',`r_4_gpa_p_star',`r_5_gpa_p_star',`r_6_gpa_p_star') ///
dec(3) row(4) col(1) location(`next')

flexmat addrow, data(\begin{tabular}[c]{@{}l@{}}Counting\\ Days\#GPA\end{tabular},`r_1_counting_days_gpa_p_star',`r_2_counting_days_gp↩
a_p_star',`r_3_counting_days_gpa_p_star',`r_4_counting_days_gpa_p_star',`r_5_counting_days_gpa_p_star',`r_6_counting_days_gpa_p_star') ///
dec(3) row(5) col(1) location(`next')

flexmat addrow, data(\begin{tabular}[c]{@{}l@{}}Female \\ (vs.
Male)\end{tabular},\;,`r_2_female_p_star',\;,`r_4_female_p_star',\;,`r_6_female_p_star') dec(3) row(6) col(1) location(`next')
flexmat addrow, data(\begin{tabular}[c]{@{}l@{}}Asian \\ (vs. White)\end{tabular},\;,`r_2_asian_p_star',\;,`r_4_asian_p_star',\;,`r_6_asian_p_star
dec(3) row(7) col(1) location(`next')
flexmat addrow, data(\begin{tabular}[c]{@{}l@{}}NonWhiteOrAsian \\ (vs. White)\end{tabular},\;,`r_2_nonwhiteorasian_p_star',\;,`r_4_nonwhiteorasia
p_star',\;,`r_6_nonwhiteorasian_p_star') dec(3) row(8) col(1) location(`next')
flexmat addrow, data(\begin{tabular}[c]{@{}l@{}}Junior \\ (vs.
Sophomore)\end{tabular},\;,`r_2_junior_p_star',\;,`r_4_junior_p_star',\;,`r_6_junior_p_star') dec(3) row(9) col(1) location(`next')
flexmat addrow, data(\begin{tabular}[c]{@{}l@{}}Senior \\ (vs.
Sophomore)\end{tabular},\;,`r_2_senior_p_star',\;,`r_4_senior_p_star',\;,`r_6_senior_p_star') dec(3) row(10) col(1) location(`next')
flexmat addrow, data(\begin{tabular}[c]{@{}l@{}}NonNativeEnglish \\ (vs. NativeEnglish)\end{tabular},\;,`r_2_nonnativeenglish↩
_p_star',\;,`r_4_nonnativeenglish_p_star',\;,`r_6_nonnativeenglish_p_star') dec(3) row(11) col(1) location(`next')
flexmat addrow, data([Intercept],`r_1_const_p_star',`r_2_const_p_star',`r_3_const_p_star',`r_4_const_p_star',`r_5_const_p_star',`r_6_const_p_star'
dec(3) row(12) col(1) location(`next')
flexmat addrow, data([Paremeter],`r_1_scale_p_star',`r_2_scale_p_star',`r_3_scale_p_star',`r_4_scale_p_star',`r_5_scale_p_star',`r_6_scale_p_star'
dec(3) row(13) col(1) location(`next')
flexmat addrow, data(\begin{tabular}[c]{@{}l@{}}Log
Likelihood\end{tabular},`r_1_log_likelihood',`r_2_log_likelihood',`r_3_log_likelihood',`r_4_log_likelihood',`r_5_log_likelihood',`r_6_log_likelih
dec(3) row(14) col(1) location(`next')
flexmat addrow, data(\begin{tabular}[c]{@{}l@{}}\# of Obs.\end{tabular},`r_1_n',`r_2_n',`r_3_n',`r_4_n',`r_5_n',`r_6_n') dec(3) row(15) col(1)
location(`next')
flexmat addrow, data($\chi^2$,`r_1_chi2_p_star',`r_2_chi2_p_star',`r_3_chi2_p_star',`r_4_chi2_p_star',`r_5_chi2_p_star',`r_6_chi2_p_star') dec(3)
row(16) col(1) location(`next')


/***
*** Fig. 4: GSEM Analysis
*** Table E4: GSEM Results
***/

* Increment the location to store a new table
loc ++next

gsem (c.practice_days c.practice_count c.gpa ///
   c.gpa#c.practice_days c.gpa#c.practice_count ///
   1.female 1.junior 1.senior 1.asian 1.nonwhiteorasian ///
   1.nonnativeenglish -> final_0_1, family(beta) link(logit)) ///
   (i.counting_days c.gpa i.counting_days#c.gpa 1.female 1.junior ///
   1.senior 1.asian 1.nonwhiteorasian 1.nonnativeenglish ->  ///
   practice_days, nbreg) ///
   (c.practice_days c.gpa 1.female 1.junior 1.senior 1.asian ///
   1.nonwhiteorasian 1.nonnativeenglish -> practice_count, nbreg) ///
   (1.female 1.junior 1.senior 1.asian 1.nonwhiteorasian ///
   1.nonnativeenglish -> gpa, )

local row_num = 1

local r_1_practice_days = string(r(table)[1,`row_num'], "%7.3f")
local r_1_practice_days_se = string(r(table)[2,`row_num'], "%7.3f")
local r_1_practice_days_p = ""
if r(table)[4,`row_num'] < 0.05 local r_1_practice_days_p = "*"
if r(table)[4,`row_num'] < 0.01 local r_1_practice_days_p = "**"
if r(table)[4,`row_num'] < 0.001 local r_1_practice_days_p = "***"

local r_1_practice_days_p_star = "\begin{tabular}[c]{@{}l@{}}b:\;`r_1_practice_days'`r_1_practice_days_p'\\ (`r_1_practice_days_se')\end{tabular}'
```

```
loc ++row_num

local r_1_practice_count = string(r(table)[1,`row_num'], "%7.3f")
local r_1_practice_count_se = string(r(table)[2,`row_num'], "%7.3f")
local r_1_practice_count_p = ""
if r(table)[4,`row_num'] < 0.05 local r_1_practice_count_p = "*"
if r(table)[4,`row_num'] < 0.01 local r_1_practice_count_p = "**"
if r(table)[4,`row_num'] < 0.001 local r_1_practice_count_p = "***"

local r_1_practice_count_p_star = "\begin{tabular}[c]{@{}l@{}}h:\;`r_1_practice_count'`r_1_practice_count_p'\\ (`r_1_practice_count_se')\end{tabul

loc ++row_num

local r_1_gpa = string(r(table)[1,`row_num'], "%7.3f")
local r_1_gpa_se = string(r(table)[2,`row_num'], "%7.3f")
local r_1_gpa_p = ""
if r(table)[4,`row_num'] < 0.05 local r_1_gpa_p = "*"
if r(table)[4,`row_num'] < 0.01 local r_1_gpa_p = "**"
if r(table)[4,`row_num'] < 0.001 local r_1_gpa_p = "***"

local r_1_gpa_p_star = "\begin{tabular}[c]{@{}l@{}}j:\;`r_1_gpa'`r_1_gpa_p'\\ (`r_1_gpa_se')\end{tabular}"

loc ++row_num

local r_1_practice_days_gpa = string(r(table)[1,`row_num'], "%7.3f")
local r_1_practice_days_gpa_se = string(r(table)[2,`row_num'], "%7.3f")
local r_1_practice_days_gpa_p = ""
if r(table)[4,`row_num'] < 0.05 local r_1_practice_days_gpa_p = "*"
if r(table)[4,`row_num'] < 0.01 local r_1_practice_days_gpa_p = "**"
if r(table)[4,`row_num'] < 0.001 local r_1_practice_days_gpa_p = "***"

local r_1_practice_days_gpa_p_star = "\begin{tabular}[c]{@{}l@{}}e:\;`r_1_practice_days_gpa'`r_1_practice_days_gpa_p'\\ (`r_1_practice_days_gp↩
a_se')\end{tabular}"

loc ++row_num

local r_1_practice_count_gpa = string(r(table)[1,`row_num'], "%7.3f")
local r_1_practice_count_gpa_se = string(r(table)[2,`row_num'], "%7.3f")
local r_1_practice_count_gpa_p = ""
if r(table)[4,`row_num'] < 0.05 local r_1_practice_count_gpa_p = "*"
if r(table)[4,`row_num'] < 0.01 local r_1_practice_count_gpa_p = "**"
if r(table)[4,`row_num'] < 0.001 local r_1_practice_count_gpa_p = "***"

local r_1_practice_count_gpa_p_star = "\begin{tabular}[c]{@{}l@{}}g:\;`r_1_practice_count_gpa'`r_1_practice_count_gpa_p'\\ (`r_1_practice_count_g
pa_se')\end{tabular}"

loc ++row_num

local r_1_female = string(r(table)[1,`row_num'], "%7.3f")
local r_1_female_se = string(r(table)[2,`row_num'], "%7.3f")
local r_1_female_p = ""
if r(table)[4,`row_num'] < 0.05 local r_1_female_p = "*"
if r(table)[4,`row_num'] < 0.01 local r_1_female_p = "**"
if r(table)[4,`row_num'] < 0.001 local r_1_female_p = "***"

local r_1_female_p_star = "\begin{tabular}[c]{@{}l@{}}`r_1_female'`r_1_female_p'\\ (`r_1_female_se')\end{tabular}"

loc ++row_num

local r_1_junior = string(r(table)[1,`row_num'], "%7.3f")
local r_1_junior_se = string(r(table)[2,`row_num'], "%7.3f")
local r_1_junior_p = ""
if r(table)[4,`row_num'] < 0.05 local r_1_junior_p = "*"
if r(table)[4,`row_num'] < 0.01 local r_1_junior_p = "**"
if r(table)[4,`row_num'] < 0.001 local r_1_junior_p = "***"

local r_1_junior_p_star = "\begin{tabular}[c]{@{}l@{}}`r_1_junior'`r_1_junior_p'\\ (`r_1_junior_se')\end{tabular}"

loc ++row_num

local r_1_senior = string(r(table)[1,`row_num'], "%7.3f")
local r_1_senior_se = string(r(table)[2,`row_num'], "%7.3f")
local r_1_senior_p = ""
if r(table)[4,`row_num'] < 0.05 local r_1_senior_p = "*"
if r(table)[4,`row_num'] < 0.01 local r_1_senior_p = "**"
```

```
if r(table)[4,`row_num'] < 0.001 local r_1_senior_p = "***"

local r_1_senior_p_star = "\begin{tabular}[c]{@{}l@{}}`r_1_senior'`r_1_senior_p'\\ (`r_1_senior_se')\end{tabular}"

loc ++row_num

local r_1_asian = string(r(table)[1,`row_num'], "%7.3f")
local r_1_asian_se = string(r(table)[2,`row_num'], "%7.3f")
local r_1_asian_p = ""
if r(table)[4,`row_num'] < 0.05 local r_1_asian_p = "*"
if r(table)[4,`row_num'] < 0.01 local r_1_asian_p = "**"
if r(table)[4,`row_num'] < 0.001 local r_1_asian_p = "***"

local r_1_asian_p_star = "\begin{tabular}[c]{@{}l@{}}`r_1_asian'`r_1_asian_p'\\ (`r_1_asian_se')\end{tabular}"

loc ++row_num

local r_1_nonwhiteorasian = string(r(table)[1,`row_num'], "%7.3f")
local r_1_nonwhiteorasian_se = string(r(table)[2,`row_num'], "%7.3f")
local r_1_nonwhiteorasian_p = ""
if r(table)[4,`row_num'] < 0.05 local r_1_nonwhiteorasian_p = "*"
if r(table)[4,`row_num'] < 0.01 local r_1_nonwhiteorasian_p = "**"
if r(table)[4,`row_num'] < 0.001 local r_1_nonwhiteorasian_p = "***"

local r_1_nonwhiteorasian_p_star = "\begin{tabular}[c]{@{}l@{}}`r_1_nonwhiteorasian'`r_1_nonwhiteorasian_p'\\ (`r_1_nonwhiteorasian_se')\end{tabul

loc ++row_num

local r_1_nonnativeenglish = string(r(table)[1,`row_num'], "%7.3f")
local r_1_nonnativeenglish_se = string(r(table)[2,`row_num'], "%7.3f")
local r_1_nonnativeenglish_p = ""
if r(table)[4,`row_num'] < 0.05 local r_1_nonnativeenglish_p = "*"
if r(table)[4,`row_num'] < 0.01 local r_1_nonnativeenglish_p = "**"
if r(table)[4,`row_num'] < 0.001 local r_1_nonnativeenglish_p = "***"

local r_1_nonnativeenglish_p_star = "\begin{tabular}[c]{@{}l@{}}`r_1_nonnativeenglish'`r_1_nonnativeenglish_p'\\ (`r_1_nonnativeenglish_se')\end{tabular}"

loc ++row_num

local r_1_const = string(r(table)[1,`row_num'], "%7.3f")
local r_1_const_se = string(r(table)[2,`row_num'], "%7.3f")
local r_1_const_p = ""
if r(table)[4,`row_num'] < 0.05 local r_1_const_p = "*"
if r(table)[4,`row_num'] < 0.01 local r_1_const_p = "**"
if r(table)[4,`row_num'] < 0.001 local r_1_const_p = "***"

local r_1_const_p_star = "\begin{tabular}[c]{@{}l@{}}`r_1_const'`r_1_const_p'\\ (`r_1_const_se')\end{tabular}"

loc ++row_num
loc ++row_num

local r_2_counting_days = string(r(table)[1,`row_num'], "%7.3f")
local r_2_counting_days_se = string(r(table)[2,`row_num'], "%7.3f")
local r_2_counting_days_p = ""
if r(table)[4,`row_num'] < 0.05 local r_2_counting_days_p = "*"
if r(table)[4,`row_num'] < 0.01 local r_2_counting_days_p = "**"
if r(table)[4,`row_num'] < 0.001 local r_2_counting_days_p = "***"

local r_2_counting_days_p_star = "\begin{tabular}[c]{@{}l@{}}a:\;`r_2_counting_days'`r_2_counting_days_p'\\ (`r_2_counting_days_se')\end{tabular}'

loc ++row_num

local r_2_gpa = string(r(table)[1,`row_num'], "%7.3f")
local r_2_gpa_se = string(r(table)[2,`row_num'], "%7.3f")
local r_2_gpa_p = ""
if r(table)[4,`row_num'] < 0.05 local r_2_gpa_p = "*"
if r(table)[4,`row_num'] < 0.01 local r_2_gpa_p = "**"
if r(table)[4,`row_num'] < 0.001 local r_2_gpa_p = "***"

local r_2_gpa_p_star = "\begin{tabular}[c]{@{}l@{}}c:\;`r_2_gpa'`r_2_gpa_p'\\ (`r_2_gpa_se')\end{tabular}"

loc ++row_num
loc ++row_num

local r_2_counting_days_gpa = string(r(table)[1,`row_num'], "%7.3f")
```

```
local r_2_counting_days_gpa_se = string(r(table)[2,`row_num'], "%7.3f")
local r_2_counting_days_gpa_p = ""
if r(table)[4,`row_num'] < 0.05 local r_2_counting_days_gpa_p = "*"
if r(table)[4,`row_num'] < 0.01 local r_2_counting_days_gpa_p = "**"
if r(table)[4,`row_num'] < 0.001 local r_2_counting_days_gpa_p = "***"

local r_2_counting_days_gpa_p_star = "\begin{tabular}[c]{@{}l@{}}b:\;`r_2_counting_days_gpa'`r_2_counting_days_gpa_p'\\ (`r_2_counting_days_gpa_se')\end{tabular}"

loc ++row_num

local r_2_female = string(r(table)[1,`row_num'], "%7.3f")
local r_2_female_se = string(r(table)[2,`row_num'], "%7.3f")
local r_2_female_p = ""
if r(table)[4,`row_num'] < 0.05 local r_2_female_p = "*"
if r(table)[4,`row_num'] < 0.01 local r_2_female_p = "**"
if r(table)[4,`row_num'] < 0.001 local r_2_female_p = "***"

local r_2_female_p_star = "\begin{tabular}[c]{@{}l@{}}`r_2_female'`r_2_female_p'\\ (`r_2_female_se')\end{tabular}"

loc ++row_num

local r_2_junior = string(r(table)[1,`row_num'], "%7.3f")
local r_2_junior_se = string(r(table)[2,`row_num'], "%7.3f")
local r_2_junior_p = ""
if r(table)[4,`row_num'] < 0.05 local r_2_junior_p = "*"
if r(table)[4,`row_num'] < 0.01 local r_2_junior_p = "**"
if r(table)[4,`row_num'] < 0.001 local r_2_junior_p = "***"

local r_2_junior_p_star = "\begin{tabular}[c]{@{}l@{}}`r_2_junior'`r_2_junior_p'\\ (`r_2_junior_se')\end{tabular}"

loc ++row_num

local r_2_senior = string(r(table)[1,`row_num'], "%7.3f")
local r_2_senior_se = string(r(table)[2,`row_num'], "%7.3f")
local r_2_senior_p = ""
if r(table)[4,`row_num'] < 0.05 local r_2_senior_p = "*"
if r(table)[4,`row_num'] < 0.01 local r_2_senior_p = "**"
if r(table)[4,`row_num'] < 0.001 local r_2_senior_p = "***"

local r_2_senior_p_star = "\begin{tabular}[c]{@{}l@{}}`r_2_senior'`r_2_senior_p'\\ (`r_2_senior_se')\end{tabular}"

loc ++row_num

local r_2_asian = string(r(table)[1,`row_num'], "%7.3f")
local r_2_asian_se = string(r(table)[2,`row_num'], "%7.3f")
local r_2_asian_p = ""
if r(table)[4,`row_num'] < 0.05 local r_2_asian_p = "*"
if r(table)[4,`row_num'] < 0.01 local r_2_asian_p = "**"
if r(table)[4,`row_num'] < 0.001 local r_2_asian_p = "***"

local r_2_asian_p_star = "\begin{tabular}[c]{@{}l@{}}`r_2_asian'`r_2_asian_p'\\ (`r_2_asian_se')\end{tabular}"

loc ++row_num

local r_2_nonwhiteorasian = string(r(table)[1,`row_num'], "%7.3f")
local r_2_nonwhiteorasian_se = string(r(table)[2,`row_num'], "%7.3f")
local r_2_nonwhiteorasian_p = ""
if r(table)[4,`row_num'] < 0.05 local r_2_nonwhiteorasian_p = "*"
if r(table)[4,`row_num'] < 0.01 local r_2_nonwhiteorasian_p = "**"
if r(table)[4,`row_num'] < 0.001 local r_2_nonwhiteorasian_p = "***"

local r_2_nonwhiteorasian_p_star = "\begin{tabular}[c]{@{}l@{}}`r_2_nonwhiteorasian'`r_2_nonwhiteorasian_p'\\ (`r_2_nonwhiteorasian_se')\end{tabul

loc ++row_num

local r_2_nonnativeenglish = string(r(table)[1,`row_num'], "%7.3f")
local r_2_nonnativeenglish_se = string(r(table)[2,`row_num'], "%7.3f")
local r_2_nonnativeenglish_p = ""
if r(table)[4,`row_num'] < 0.05 local r_2_nonnativeenglish_p = "*"
if r(table)[4,`row_num'] < 0.01 local r_2_nonnativeenglish_p = "**"
if r(table)[4,`row_num'] < 0.001 local r_2_nonnativeenglish_p = "***"

local r_2_nonnativeenglish_p_star = "\begin{tabular}[c]{@{}l@{}}`r_2_nonnativeenglish'`r_2_nonnativeenglish_p'\\ (`r_2_nonnativeenglish_se')\end{tabular}"

```

```
loc ++row_num

local r_2_const = string(r(table)[1,`row_num'], "%7.3f")
local r_2_const_se = string(r(table)[2,`row_num'], "%7.3f")
local r_2_const_p = ""
if r(table)[4,`row_num'] < 0.05 local r_2_const_p = "*"
if r(table)[4,`row_num'] < 0.01 local r_2_const_p = "**"
if r(table)[4,`row_num'] < 0.001 local r_2_const_p = "***"

local r_2_const_p_star = "\begin{tabular}[c]{@{}l@{}}`r_2_const'`r_2_const_p'\\ (`r_2_const_se')\end{tabular}"

loc ++row_num
loc ++row_num

local r_3_practice_days = string(r(table)[1,`row_num'], "%7.3f")
local r_3_practice_days_se = string(r(table)[2,`row_num'], "%7.3f")
local r_3_practice_days_p = ""
if r(table)[4,`row_num'] < 0.05 local r_3_practice_days_p = "*"
if r(table)[4,`row_num'] < 0.01 local r_3_practice_days_p = "**"
if r(table)[4,`row_num'] < 0.001 local r_3_practice_days_p = "***"

local r_3_practice_days_p_star = "\begin{tabular}[c]{@{}l@{}}i:\;`r_3_practice_days'`r_3_practice_days_p'\\ (`r_3_practice_days_se')\end{tabular}'

loc ++row_num

local r_3_gpa = string(r(table)[1,`row_num'], "%7.3f")
local r_3_gpa_se = string(r(table)[2,`row_num'], "%7.3f")
local r_3_gpa_p = ""
if r(table)[4,`row_num'] < 0.05 local r_3_gpa_p = "*"
if r(table)[4,`row_num'] < 0.01 local r_3_gpa_p = "**"
if r(table)[4,`row_num'] < 0.001 local r_3_gpa_p = "***"

local r_3_gpa_p_star = "\begin{tabular}[c]{@{}l@{}}f:\;`r_3_gpa'`r_3_gpa_p'\\ (`r_3_gpa_se')\end{tabular}"

loc ++row_num

local r_3_female = string(r(table)[1,`row_num'], "%7.3f")
local r_3_female_se = string(r(table)[2,`row_num'], "%7.3f")
local r_3_female_p = ""
if r(table)[4,`row_num'] < 0.05 local r_3_female_p = "*"
if r(table)[4,`row_num'] < 0.01 local r_3_female_p = "**"
if r(table)[4,`row_num'] < 0.001 local r_3_female_p = "***"

local r_3_female_p_star = "\begin{tabular}[c]{@{}l@{}}`r_3_female'`r_3_female_p'\\ (`r_3_female_se')\end{tabular}"

loc ++row_num

local r_3_junior = string(r(table)[1,`row_num'], "%7.3f")
local r_3_junior_se = string(r(table)[2,`row_num'], "%7.3f")
local r_3_junior_p = ""
if r(table)[4,`row_num'] < 0.05 local r_3_junior_p = "*"
if r(table)[4,`row_num'] < 0.01 local r_3_junior_p = "**"
if r(table)[4,`row_num'] < 0.001 local r_3_junior_p = "***"

local r_3_junior_p_star = "\begin{tabular}[c]{@{}l@{}}`r_3_junior'`r_3_junior_p'\\ (`r_3_junior_se')\end{tabular}"

loc ++row_num

local r_3_senior = string(r(table)[1,`row_num'], "%7.3f")
local r_3_senior_se = string(r(table)[2,`row_num'], "%7.3f")
local r_3_senior_p = ""
if r(table)[4,`row_num'] < 0.05 local r_3_senior_p = "*"
if r(table)[4,`row_num'] < 0.01 local r_3_senior_p = "**"
if r(table)[4,`row_num'] < 0.001 local r_3_senior_p = "***"

local r_3_senior_p_star = "\begin{tabular}[c]{@{}l@{}}`r_3_senior'`r_3_senior_p'\\ (`r_3_senior_se')\end{tabular}"

loc ++row_num

local r_3_asian = string(r(table)[1,`row_num'], "%7.3f")
local r_3_asian_se = string(r(table)[2,`row_num'], "%7.3f")
local r_3_asian_p = ""
if r(table)[4,`row_num'] < 0.05 local r_3_asian_p = "*"
if r(table)[4,`row_num'] < 0.01 local r_3_asian_p = "**"
if r(table)[4,`row_num'] < 0.001 local r_3_asian_p = "***"
```

```
local r_3_asian_p_star = "\begin{tabular}[c]{@{}l@{}}`r_3_asian'`r_3_asian_p'\\ (`r_3_asian_se')\end{tabular}"

loc ++row_num

local r_3_nonwhiteorasian = string(r(table)[1,`row_num'], "%7.3f")
local r_3_nonwhiteorasian_se = string(r(table)[2,`row_num'], "%7.3f")
local r_3_nonwhiteorasian_p = ""
if r(table)[4,`row_num'] < 0.05 local r_3_nonwhiteorasian_p = "*"
if r(table)[4,`row_num'] < 0.01 local r_3_nonwhiteorasian_p = "**"
if r(table)[4,`row_num'] < 0.001 local r_3_nonwhiteorasian_p = "***"

local r_3_nonwhiteorasian_p_star = "\begin{tabular}[c]{@{}l@{}}`r_3_nonwhiteorasian'`r_3_nonwhiteorasian_p'\\ (`r_3_nonwhiteorasian_se')\end{tabul

loc ++row_num

local r_3_nonnativeenglish = string(r(table)[1,`row_num'], "%7.3f")
local r_3_nonnativeenglish_se = string(r(table)[2,`row_num'], "%7.3f")
local r_3_nonnativeenglish_p = ""
if r(table)[4,`row_num'] < 0.05 local r_3_nonnativeenglish_p = "*"
if r(table)[4,`row_num'] < 0.01 local r_3_nonnativeenglish_p = "**"
if r(table)[4,`row_num'] < 0.001 local r_3_nonnativeenglish_p = "***"

local r_3_nonnativeenglish_p_star = "\begin{tabular}[c]{@{}l@{}}`r_3_nonnativeenglish'`r_3_nonnativeenglish_p'\\ (`r_3_nonnativeenglish_se')\end{tabular}"

loc ++row_num

local r_3_const = string(r(table)[1,`row_num'], "%7.3f")
local r_3_const_se = string(r(table)[2,`row_num'], "%7.3f")
local r_3_const_p = ""
if r(table)[4,`row_num'] < 0.05 local r_3_const_p = "*"
if r(table)[4,`row_num'] < 0.01 local r_3_const_p = "**"
if r(table)[4,`row_num'] < 0.001 local r_3_const_p = "***"

local r_3_const_p_star = "\begin{tabular}[c]{@{}l@{}}`r_3_const'`r_3_const_p'\\ (`r_3_const_se')\end{tabular}"

loc ++row_num
loc ++row_num

local r_4_female = string(r(table)[1,`row_num'], "%7.3f")
local r_4_female_se = string(r(table)[2,`row_num'], "%7.3f")
local r_4_female_p = ""
if r(table)[4,`row_num'] < 0.05 local r_4_female_p = "*"
if r(table)[4,`row_num'] < 0.01 local r_4_female_p = "**"
if r(table)[4,`row_num'] < 0.001 local r_4_female_p = "***"

local r_4_female_p_star = "\begin{tabular}[c]{@{}l@{}}`r_4_female'`r_4_female_p'\\ (`r_4_female_se')\end{tabular}"

loc ++row_num

local r_4_junior = string(r(table)[1,`row_num'], "%7.3f")
local r_4_junior_se = string(r(table)[2,`row_num'], "%7.3f")
local r_4_junior_p = ""
if r(table)[4,`row_num'] < 0.05 local r_4_junior_p = "*"
if r(table)[4,`row_num'] < 0.01 local r_4_junior_p = "**"
if r(table)[4,`row_num'] < 0.001 local r_4_junior_p = "***"

local r_4_junior_p_star = "\begin{tabular}[c]{@{}l@{}}`r_4_junior'`r_4_junior_p'\\ (`r_4_junior_se')\end{tabular}"

loc ++row_num

local r_4_senior = string(r(table)[1,`row_num'], "%7.3f")
local r_4_senior_se = string(r(table)[2,`row_num'], "%7.3f")
local r_4_senior_p = ""
if r(table)[4,`row_num'] < 0.05 local r_4_senior_p = "*"
if r(table)[4,`row_num'] < 0.01 local r_4_senior_p = "**"
if r(table)[4,`row_num'] < 0.001 local r_4_senior_p = "***"

local r_4_senior_p_star = "\begin{tabular}[c]{@{}l@{}}`r_4_senior'`r_4_senior_p'\\ (`r_4_senior_se')\end{tabular}"

loc ++row_num

local r_4_asian = string(r(table)[1,`row_num'], "%7.3f")
local r_4_asian_se = string(r(table)[2,`row_num'], "%7.3f")
local r_4_asian_p = ""
if r(table)[4,`row_num'] < 0.05 local r_4_asian_p = "*"
```

```
if r(table)[4,`row_num'] < 0.01 local r_4_asian_p = "**"
if r(table)[4,`row_num'] < 0.001 local r_4_asian_p = "***"

local r_4_asian_p_star = "\begin{tabular}[c]{@{}l@{}}`r_4_asian'`r_4_asian_p'\\ (`r_4_asian_se')\end{tabular}"

loc ++row_num

local r_4_nonwhiteorasian = string(r(table)[1,`row_num'], "%7.3f")
local r_4_nonwhiteorasian_se = string(r(table)[2,`row_num'], "%7.3f")
local r_4_nonwhiteorasian_p = ""
if r(table)[4,`row_num'] < 0.05 local r_4_nonwhiteorasian_p = "*"
if r(table)[4,`row_num'] < 0.01 local r_4_nonwhiteorasian_p = "**"
if r(table)[4,`row_num'] < 0.001 local r_4_nonwhiteorasian_p = "***"

local r_4_nonwhiteorasian_p_star = "\begin{tabular}[c]{@{}l@{}}`r_4_nonwhiteorasian'`r_4_nonwhiteorasian_p'\\ (`r_4_nonwhiteorasian_se')\end{tabul

loc ++row_num

local r_4_nonnativeenglish = string(r(table)[1,`row_num'], "%7.3f")
local r_4_nonnativeenglish_se = string(r(table)[2,`row_num'], "%7.3f")
local r_4_nonnativeenglish_p = ""
if r(table)[4,`row_num'] < 0.05 local r_4_nonnativeenglish_p = "*"
if r(table)[4,`row_num'] < 0.01 local r_4_nonnativeenglish_p = "**"
if r(table)[4,`row_num'] < 0.001 local r_4_nonnativeenglish_p = "***"

local r_4_nonnativeenglish_p_star = "\begin{tabular}[c]{@{}l@{}}`r_4_nonnativeenglish'`r_4_nonnativeenglish_p'\\ (`r_4_nonnativeenglish↩
_se')\end{tabular}"

loc ++row_num

local r_4_const = string(r(table)[1,`row_num'], "%7.3f")
local r_4_const_se = string(r(table)[2,`row_num'], "%7.3f")
local r_4_const_p = ""
if r(table)[4,`row_num'] < 0.05 local r_4_const_p = "*"
if r(table)[4,`row_num'] < 0.01 local r_4_const_p = "**"
if r(table)[4,`row_num'] < 0.001 local r_4_const_p = "***"

local r_4_const_p_star = "\begin{tabular}[c]{@{}l@{}}`r_4_const'`r_4_const_p'\\ (`r_4_const_se')\end{tabular}"

local r_1_parameter = string(r(table)[1,41], "%7.3f")
local r_1_parameter_se = string(r(table)[2,41], "%7.3f")

local r_1_parameter_p_star = "\begin{tabular}[c]{@{}l@{}}logs:\\ `r_1_parameter'\\ (`r_1_parameter_se')\end{tabular}"

local r_2_parameter = string(r(table)[1,42], "%7.3f")
local r_2_parameter_se = string(r(table)[2,42], "%7.3f")

local r_2_parameter_p_star = "\begin{tabular}[c]{@{}l@{}}lnalpha:\\ `r_2_parameter'\\ (`r_2_parameter_se')\end{tabular}"

local r_3_parameter = string(r(table)[1,43], "%7.3f")
local r_3_parameter_se = string(r(table)[2,43], "%7.3f")

local r_3_parameter_p_star = "\begin{tabular}[c]{@{}l@{}}lnalpha:\\ `r_3_parameter'\\ (`r_3_parameter_se')\end{tabular}"

local r_4_parameter = string(r(table)[1,44], "%7.3f")
local r_4_parameter_se = string(r(table)[2,44], "%7.3f")

local r_4_parameter_p_star = "\begin{tabular}[c]{@{}l@{}}var(e.gpa):\\ `r_4_parameter'\\ (`r_4_parameter_se')\end{tabular}"

* Increment the location to store a new table
loc ++next

flexmat addparts, title(Table E4) location(`next')
flexmat addcell, data(\textbf{\begin{tabular}[c]{@{}c@{}}Final Exam\\ Score\end{tabular}}) row(1) col(2) location(`next')
flexmat addcell, data(\textbf{\begin{tabular}[c]{@{}c@{}}Practice\\ Days\end{tabular}}) row(1) col(3) location(`next')
flexmat addcell, data(\textbf{\begin{tabular}[c]{@{}c@{}}Practice\\ Time\end{tabular}}) row(1) col(4) location(`next')
flexmat addcell, data(\textbf{\begin{tabular}[c]{@{}c@{}}GPA\end{tabular}}) row(1) col(5) location(`next')

flexmat addrow, data(\begin{tabular}[c]{@{}l@{}}Counting Days\\ (vs. Questions)\end{tabular},\;,`r_2_counting_days_p_star',\;,\;) ///
dec(3) row(2) col(1) location(`next')

flexmat addrow, data(\begin{tabular}[c]{@{}l@{}}Counting\\ Days\#GPA\end{tabular},\;,`r_2_counting_days_gpa_p_star',\;,\;) ///
dec(3) row(3) col(1) location(`next')

flexmat addrow, data(\begin{tabular}[c]{@{}c@{}}Practice\\ Days\end{tabular},`r_1_practice_days_p_star',\;,`r_3_practice_days_p_star',\;) ///
dec(3) row(4) col(1) location(`next')
```

```
flexmat addrow, data(\begin{tabular}[c]{@{}l@{}}Practice\\ Days\#GPA\end{tabular},`r_1_practice_days_gpa_p_star',\;,\;,\;) ///
dec(3) row(5) col(1) location(`next')

flexmat addrow, data(\begin{tabular}[c]{@{}l@{}}Practice\\ Time\end{tabular},`r_1_practice_count_p_star',\;,\;,\;) ///
dec(3) row(6) col(1) location(`next')

flexmat addrow, data(\begin{tabular}[c]{@{}l@{}}Practice\\ Time\#GPA\end{tabular},`r_1_practice_count_gpa_p_star',\;,\;,\;) ///
dec(3) row(7) col(1) location(`next')

flexmat addrow, data(GPA,`r_1_gpa_p_star',`r_2_gpa_p_star',`r_3_gpa_p_star',\;) ///
dec(3) row(8) col(1) location(`next')

flexmat addrow, data(\begin{tabular}[c]{@{}l@{}}Female \\ (vs. Male)\end{tabular},`r_1_female_p_star',`r_2_female_p_star',`r_3_female_p_star',`r_4_female_p_star') dec(3) row(9) col(1) location(`next')
flexmat addrow, data(\begin{tabular}[c]{@{}l@{}}Asian \\ (vs. White)\end{tabular},`r_1_asian_p_star',`r_2_asian_p_star',`r_3_asian_p_star',`r_4_asian_p_star') dec(3) row(10) col(1) location(`next')
flexmat addrow, data(\begin{tabular}[c]{@{}l@{}}NonWhiteOrAsian \\ (vs. White)\end{tabular},`r_1_nonwhiteorasian_p_star',`r_2_nonwhiteorasian_p_star',`r_3_nonwhiteorasian_p_star',`r_4_nonwhiteorasian_p_star') dec(3) row(11) col(1) location(`next')
flexmat addrow, data(\begin{tabular}[c]{@{}l@{}}Junior \\ (vs. Sophomore)\end{tabular},`r_1_junior_p_star',`r_2_junior_p_star',`r_3_junior_p_star',`r_4_junior_p_star') dec(3) row(12) col(1) location(`next')
flexmat addrow, data(\begin{tabular}[c]{@{}l@{}}Senior \\ (vs. Sophomore)\end{tabular},`r_1_senior_p_star',`r_2_senior_p_star',`r_3_senior_p_star',`r_4_senior_p_star') dec(3) row(13) col(1) location(`next')
flexmat addrow, data(\begin{tabular}[c]{@{}l@{}}NonNativeEnglish \\ (vs. NativeEnglish)\end{tabular},`r_1_nonnativeenglish_p_star',`r_2_nonnativeenglish_p_star',`r_3_nonnativeenglish_p_star',`r_4_nonnativeenglish_p_star') dec(3) row(14) col(1) location(`next')
flexmat addrow, data([Intercept],`r_1_const_p_star',`r_2_const_p_star',`r_3_const_p_star',`r_4_const_p_star') dec(3) row(15) col(1) location(`nex
flexmat addrow, data([Paremeter],`r_1_parameter_p_star',`r_2_parameter_p_star',`r_3_parameter_p_star',`r_4_parameter_p_star') dec(3) row(16) col(
location(`next')

asdocx export, save(Tables.tex)

// Calculate the non-linear combination of the confidence intervals to approximate the statistical significance of the meditation effect.
nlcom (product: _b[final_0_1:practice_days]*_b[practice_days:1.counting_days])
nlcom (product: _b[final_0_1:c.gpa#practice_days]*_b[practice_days:1.counting_days])
nlcom (product: _b[final_0_1:practice_count]*_b[practice_count:practice_days]*_b[practice_days:1.counting_days])
nlcom (product: _b[final_0_1:c.gpa#practice_count]*_b[practice_count:practice_days]*_b[practice_days:1.counting_days])

/***
*** Fig. 5
Estimated effects of GPA on number of days practiced for each of the experimental conditions.
***/

// margins i.counting_days, predict(outcome(practice_days)) at(gpa=(2(0.5)4)) atmeans
//
// marginsplot, ///
// ci1opts(lcolor(navy) fcolor(navy*0.7)) ///
// ci2opts(lcolor(maroon) fcolor(maroon*0.7)) ///
// addplot( ///
// scatteri 58 2  58 `gpa_1st', connect(line) mcolor(none) lwidth(vvvthick) ///
// lcolor(red%40) || ///
// scatteri 54.382 2  54.382 `gpa_1st', connect(line) mcolor(none) lwidth(vvvthick) ///
// lcolor(red%40) || ///
// scatteri 58 `gpa_1st' 58 `gpa_2nd', connect(line) mcolor(none) lwidth(vvvthick) ///
// lcolor(yellow%40) || ///
// scatteri 54.382 `gpa_1st' 54.382 `gpa_2nd', connect(line) mcolor(none) lwidth(vvvthick) ///
// lcolor(yellow%40) || ///
// scatteri 58 `gpa_2nd'  58 4, connect(line) mcolor(none) lwidth(vvvthick) ///
// lcolor(green%40) || ///
// scatteri 54.382 `gpa_2nd'  54.382 4, connect(line) mcolor(none) lwidth(vvvthick) ///
// lcolor(green%40) || ///
// scatter practice_days gpa ///
// if counting_days == 0, mcolor(navy) || ///
// scatter practice_days gpa ///
// if counting_days == 1, mcolor(maroon) ///
// yscale(range(0 57.1)) ///
// legend(order(1 "Counting Questions" 2 "Counting Days")) ///
// text(58 2.65 "Low GPA" 58 `gpa_mid' "Mid GPA" 58 3.85 "High GPA" ///
//  54.382 2.65 "1st Tercile" 54.382 `gpa_mid' "2nd Tercile" ///
//  54.382 3.85 "3rd Tercile")) ///
// xmlabel(`gpa_1st' "`gpa_1st'" `gpa_2nd' "`gpa_2nd'") ///
// recast(line) recastci(rarea) ///
// xline(`gpa_1st' `gpa_2nd') ///
// title("") ///
// xtitle("GPA") ytitle("# of Days Practiced")
```

```
/***
*** Fig. F2:
Estimated effect of GPA on final exam scores for different numbers of days practiced.
***/

// Calculate the practice_days standard deviation.
sum practice_days

// Define min_practice_days as the minimum practice_days.
local min_practice_days = r(min)

// Define mean_practice_days as the average practice_days.
local mean_practice_days = r(mean)

// Define max_practice_days as the maximum practice_days.
local max_practice_days = r(max)

// Define the practice_days terciles.
pctile practice_days_terciles = practice_days, nq(3)
// GPA 1st tercile.
local practice_days_1st = round(r(r1), 0.01)
// GPA 2nd tercile.
local practice_days_2nd = round(r(r2), 0.01)

margins, predict(outcome(final_0_1)) at(gpa=(2(0.5)4) ///
practice_days=(`min_practice_days' `practice_days_1st' `practice_days_2nd' ///
`max_practice_days'))

marginsplot, plot1opts(lcolor(navy)) plot2opts(lcolor(dkgreen)) ///
plot3opts(lcolor(dkorange)) plot4opts(lcolor(maroon)) ///
ci1opts(lcolor(navy) fcolor(navy*0.7)) ///
ci2opts(lcolor(dkgreen) fcolor(dkgreen*0.7)) ///
ci3opts(lcolor(dkorange) fcolor(dkorange*0.7)) ///
ci4opts(lcolor(maroon) fcolor(maroon*0.7)) ///
addplot( ///
scatteri 0.2 2  0.2 `gpa_1st', connect(line) mcolor(none) lwidth(vvvthick) ///
lcolor(red%40) || ///
scatteri 0.1446 2  0.1446 `gpa_1st', connect(line) mcolor(none) lwidth(vvvthick) ///
lcolor(red%40) || ///
scatteri 0.2 `gpa_1st' 0.2 `gpa_2nd', connect(line) mcolor(none) lwidth(vvvthick) ///
lcolor(yellow%40) || ///
scatteri 0.1446 `gpa_1st' 0.1446 `gpa_2nd', connect(line) mcolor(none) lwidth(vvvthick) ///
lcolor(yellow%40) || ///
scatteri 0.2 `gpa_2nd' 0.2 4, connect(line) mcolor(none) lwidth(vvvthick) ///
lcolor(green%40) || ///
scatteri 0.1446 `gpa_2nd'  0.1446 4, connect(line) mcolor(none) lwidth(vvvthick) ///
lcolor(green%40) || ///
scatter final_0_1 gpa ///
if practice_days == 0 & gpa != 0 & final != 0 & ///
gradingtype == "Graded" & term == "FA 2018", mcolor(navy) || ///
scatter final_0_1 gpa ///
if practice_days > 0 & practice_days <= `practice_days_1st', mcolor(dkgreen) || ///
scatter final_0_1 gpa ///
if practice_days >= `practice_days_1st' & practice_days < `practice_days_2nd', ///
mcolor(dkorange) || ///
scatter final_0_1 gpa ///
if practice_days > `practice_days_2nd', mcolor(maroon) ///
legend(order(1 "Minimum = 0 Days" 2 "1st Tercile = 16 Days" ///
3 "2nd Tercile = 42 Days" 4 "Maximum = 49 Days")) ///
text(0.2 2.65 "Low GPA" 0.2 `gpa_mid' "Mid GPA" 0.2 3.85 "High GPA" ///
    0.1446 2.65 "1st Tercile" 0.1446 `gpa_mid' "2nd Tercile" ///
    0.1446 3.85 "3rd Tercile")) ///
ylab(.2 "20%" .4 "40%" .6 "60%" .8 "80%" 1 "100%") ///
xmlabel(`gpa_1st' "`gpa_1st'" `gpa_2nd' "`gpa_2nd'") ///
recast(line) recastci(rarea) ///
xline(`gpa_1st' `gpa_2nd', lcolor(gray)) ///
title("") ///
xtitle("GPA") ytitle("Predicted Mean (Final Exam Score)")


/***
*** Winter 2018 Data Analysis ***
***/

* Clear the memory before starting. *
clear
```

```
* Specify the path *
cd "$mypath"

// Load the dataset again.
import delimited "CountingDaysFourSemesterData.csv"

// The GPA field was populated from an inner join query with student data from
// the registrar. GPA field is blank when the student was not registered for
// this course in this semester (i.e., TAs and other system testers).

// We exclude those students who did not take the final exam; i.e., withdrew the
// course (at the end of the semester, after paying the tuition) or failed.

//We exclude those students who took the course for purposes other than getting
// a letter grade, such as auditing.
keep if gradingtype == "Graded" & final > 0 & final <= 100 & ///
gpa > 0 & gpa <= 4 & term == "WN 2018"

// After excluding those who have no GPA, a few Freshmen remained. We treat them
// as Sophomores because they had taken courses in the previous semester.
replace sophomore = 1 if freshman == 1
replace academiclevel = "Sophomore" if freshman == 1

// Calculate the GPA standard deviation.
sum gpa

// Define min_gpa as the minimum GPA.
local min_gpa = r(min)

// Define mean_gpa as the average GPA.
local mean_gpa = r(mean)

// Define max_gpa as the maximum GPA.
local max_gpa = r(max)

// Define low_gpa as one standard deviation below the mean GPA.
local low_gpa = r(mean) - r(sd)

// Define low_gpa as one standard deviation above the mean GPA.
local high_gpa = r(mean) + r(sd)

// Define the gap terciles.
pctile gap_terciles = gpa, nq(3)
// GPA 1st tercile.
local gpa_1st = round(r(r1), 0.01)
// GPA 2nd tercile.
local gpa_2nd = round(r(r2), 0.01)
local gpa_mid = round((`gpa_1st' + `gpa_2nd')/2, 0.01)

/***
*** Fig. G3
***/

betareg final_0_1 c.gpa ///
1.female 1.junior 1.senior 1.asian 1.nonwhiteorasian 1.nonnativeenglish

margins, at(gpa=(2(0.5)4)) atmeans

marginsplot, ///
addplot( ///
scatteri 0.2 2  0.2 `gpa_1st', connect(line) mcolor(none) lwidth(vvvthick) ///
lcolor(red%40) || ///
scatteri 0.1549 2  0.1549 `gpa_1st', connect(line) mcolor(none) lwidth(vvvthick) ///
lcolor(red%40) || ///
scatteri 0.2 `gpa_1st' 0.2 `gpa_2nd', connect(line) mcolor(none) lwidth(vvvthick) ///
lcolor(yellow%40) || ///
scatteri 0.1549 `gpa_1st' 0.1549 `gpa_2nd', connect(line) mcolor(none) lwidth(vvvthick) ///
lcolor(yellow%40) || ///
scatteri 0.2 `gpa_2nd' 0.2 4, connect(line) mcolor(none) lwidth(vvvthick) ///
lcolor(green%40) || ///
scatteri 0.1549 `gpa_2nd'  0.1549 4, connect(line) mcolor(none) lwidth(vvvthick) ///
lcolor(green%40) || ///
scatter final_0_1 gpa, ///
legend(off) ///
text(0.2 2.65 "Low GPA" 0.2 `gpa_mid' "Mid GPA" 0.2 3.85 "High GPA" ///
```

```
    0.1549 2.65 "1st Tercile" 0.1549 `gpa_mid' "2nd Tercile" ///
    0.1549 3.85 "3rd Tercile")) ///
ylab(.2 "20%" .4 "40%" .6 "60%" .8 "80%" 1 "100%") ///
xmlabel(`gpa_1st' "`gpa_1st'" `gpa_2nd' "`gpa_2nd'") ///
recast(line) plotopts(color(maroon)) ciopt(color(maroon)) recastci(rarea) ///
xline(`gpa_1st' `gpa_2nd', lcolor(gray)) ///
title("") ///
xtitle("GPA") ytitle("Conditional Mean of Final Exam Score")

/***
*** Winter 2019 Data Analysis ***
***/

* Clear the memory before starting. *
clear

* Specify the path *
cd "$mypath"

// Load the dataset again.
import delimited "CountingDaysFourSemesterData.csv"

// The GPA field was populated from an inner join query with student data from
// the registrar. GPA field is blank when the student was not registered for
// this course in this semester (i.e., TAs and other system testers).

// We exclude those students who did not take the final exam; i.e., withdrew the
// course (at the end of the semester, after paying the tuition) or failed.

//We exclude those students who took the course for purposes other than getting
// a letter grade, such as auditing.
keep if gradingtype == "Graded" & final > 0 & final <= 100 & ///
gpa > 0 & gpa <= 4 & term == "WN 2019"

// After excluding those who have no GPA, a few Freshmen remained. We treat them
// as Sophomores because they had taken courses in the previous semester.
replace sophomore = 1 if freshman == 1
replace academiclevel = "Sophomore" if freshman == 1

// Calculate the GPA standard deviation.
sum gpa

// Define min_gpa as the minimum GPA.
local min_gpa = r(min)

// Define mean_gpa as the average GPA.
local mean_gpa = r(mean)

// Define max_gpa as the maximum GPA.
local max_gpa = r(max)

// Define low_gpa as one standard deviation below the mean GPA.
local low_gpa = r(mean) - r(sd)

// Define low_gpa as one standard deviation above the mean GPA.
local high_gpa = r(mean) + r(sd)

// Define the gap terciles.
pctile gap_terciles = gpa, nq(3)
// GPA 1st tercile.
local gpa_1st = round(r(r1), 0.01)
// GPA 2nd tercile.
local gpa_2nd = round(r(r2), 0.01)
local gpa_mid = round((`gpa_1st' + `gpa_2nd')/2, 0.01)

/***
*** Fig. G4
***/

betareg final_0_1 c.gpa ///
1.female 1.junior 1.senior 1.asian 1.nonwhiteorasian 1.nonnativeenglish

margins, at(gpa=(2(0.5)4)) atmeans

marginsplot, ///
addplot( ///
```

```
scatteri 0.2 2  0.2 `gpa_1st', connect(line) mcolor(none) lwidth(vvvthick) ///
lcolor(red%40) || ///
scatteri 0.1549 2  0.1549 `gpa_1st', connect(line) mcolor(none) lwidth(vvvthick) ///
lcolor(red%40) || ///
scatteri 0.2 `gpa_1st' 0.2 `gpa_2nd', connect(line) mcolor(none) lwidth(vvvthick) ///
lcolor(yellow%40) || ///
scatteri 0.1549 `gpa_1st' 0.1549 `gpa_2nd', connect(line) mcolor(none) lwidth(vvvthick) ///
lcolor(yellow%40) || ///
scatteri 0.2 `gpa_2nd' 0.2 4, connect(line) mcolor(none) lwidth(vvvthick) ///
lcolor(green%40) || ///
scatteri 0.1549 `gpa_2nd'  0.1549 4, connect(line) mcolor(none) lwidth(vvvthick) ///
lcolor(green%40) || ///
scatter final_0_1 gpa, ///
legend(off) ///
text(0.2 2.65 "Low GPA" 0.2 `gpa_mid' "Mid GPA" 0.2 3.85 "High GPA" ///
    0.1549 2.65 "1st Tercile" 0.1549 `gpa_mid' "2nd Tercile" ///
    0.1549 3.85 "3rd Tercile")) ///
ylab(.2 "20%" .4 "40%" .6 "60%" .8 "80%" 1 "100%") ///
xmlabel(`gpa_1st' "`gpa_1st'" `gpa_2nd' "`gpa_2nd'") ///
recast(line) plotopts(color(maroon)) ciopt(color(maroon)) recastci(rarea) ///
xline(`gpa_1st' `gpa_2nd', lcolor(gray)) ///
title("") ///
xtitle("GPA") ytitle("Conditional Mean of Final Exam Score")

/***
*** Fall 2019 Data Analysis ***
***/

* Clear the memory before starting. *
clear

* Specify the path *
cd "$mypath"

// Load the dataset again.
import delimited "CountingDaysFourSemesterData.csv"

// The GPA field was populated from an inner join query with student data from
// the registrar. GPA field is blank when the student was not registered for
// this course in this semester (i.e., TAs and other system testers).

// We exclude those students who did not take the final exam; i.e., withdrew the
// course (at the end of the semester, after paying the tuition) or failed.

//We exclude those students who took the course for purposes other than getting
// a letter grade, such as auditing.
keep if gradingtype == "Graded" & final > 0 & final <= 100 & ///
gpa > 0 & gpa <= 4 & term == "FA 2019"

// After excluding those who have no GPA, a few Freshmen remained. We treat them
// as Sophomores because they had taken courses in the previous semester.
replace sophomore = 1 if freshman == 1
replace academiclevel = "Sophomore" if freshman == 1

// Calculate the GPA standard deviation.
sum gpa

// Define min_gpa as the minimum GPA.
local min_gpa = r(min)

// Define mean_gpa as the average GPA.
local mean_gpa = r(mean)

// Define max_gpa as the maximum GPA.
local max_gpa = r(max)

// Define low_gpa as one standard deviation below the mean GPA.
local low_gpa = r(mean) - r(sd)

// Define low_gpa as one standard deviation above the mean GPA.
local high_gpa = r(mean) + r(sd)

// Define the gap terciles.
pctile gap_terciles = gpa, nq(3)
// GPA 1st tercile.
local gpa_1st = round(r(r1), 0.01)
```

```
// GPA 2nd tercile.
local gpa_2nd = round(r(r2), 0.01)
local gpa_mid = round((`gpa_1st' + `gpa_2nd')/2, 0.01)

/***
*** Fig. G5
***/

betareg final_0_1 c.gpa ///
1.female 1.junior 1.senior 1.asian 1.nonwhiteorasian 1.nonnativeenglish

margins, at(gpa=(2(0.5)4)) atmeans

marginsplot, ///
addplot( ///
scatteri 0.2 2  0.2 `gpa_1st', connect(line) mcolor(none) lwidth(vvvthick) ///
lcolor(red%40) || ///
scatteri 0.1549 2  0.1549 `gpa_1st', connect(line) mcolor(none) lwidth(vvvthick) ///
lcolor(red%40) || ///
scatteri 0.2 `gpa_1st' 0.2 `gpa_2nd', connect(line) mcolor(none) lwidth(vvvthick) ///
lcolor(yellow%40) || ///
scatteri 0.1549 `gpa_1st' 0.1549 `gpa_2nd', connect(line) mcolor(none) lwidth(vvvthick) ///
lcolor(yellow%40) || ///
scatteri 0.2 `gpa_2nd' 0.2 4, connect(line) mcolor(none) lwidth(vvvthick) ///
lcolor(green%40) || ///
scatteri 0.1549 `gpa_2nd'  0.1549 4, connect(line) mcolor(none) lwidth(vvvthick) ///
lcolor(green%40) || ///
scatter final_0_1 gpa, ///
legend(off) ///
text(0.2 2.65 "Low GPA" 0.2 `gpa_mid' "Mid GPA" 0.2 3.85 "High GPA" ///
    0.1549 2.65 "1st Tercile" 0.1549 `gpa_mid' "2nd Tercile" ///
    0.1549 3.85 "3rd Tercile")) ///
ylab(.2 "20%" .4 "40%" .6 "60%" .8 "80%" 1 "100%") ///
xmlabel(`gpa_1st' "`gpa_1st'" `gpa_2nd' "`gpa_2nd'") ///
recast(line) plotopts(color(maroon)) ciopt(color(maroon)) recastci(rarea) ///
xline(`gpa_1st' `gpa_2nd', lcolor(gray)) ///
title("") ///
xtitle("GPA") ytitle("Conditional Mean of Final Exam Score")


/***
*** Between-instructor Experiment Analysis ***
***/

* Clear the memory before starting. *
clear

* Specify the path *
cd "$mypath"

// Load the dataset again.
import delimited "Between_Instructor_Data.csv"

gen counting_days = 0
replace counting_days = 1 if spacing == "Counting Days"

tabulate counting_days

/***
*** Table 9
***/
// Comparing the outcomes across the two conditions using two-sided t-test and
// Wilcoxon rank sum test.

*Store the first table in location 1
local next 1

flexmat addparts, title(Table E7) location(`next')
flexmat addcell, data(t-test) row(1) col(2) location(`next')
flexmat addcell, data(Wilcoxon rank-sum) row(1) col(3) location(`next')

// Practice Days
ttest practice_days, by(counting_days)

local r_t = string(`r(t)', "%7.3f")
local r_t_p = ""
```

```
if `r(p_l)' < 0.05 local r_t_p = "*"
if `r(p_l)' < 0.01 local r_t_p = "**"
if `r(p_l)' < 0.001 local r_t_p = "***"
local r_t_p_star = "`r_t'`r_t_p'"

ranksum practice_days, by(counting_days)

local r_z = string(`r(z)', "%7.3f")
local r_z_p = ""
if `r(p_l)' < 0.05 local r_z_p = "*"
if `r(p_l)' < 0.01 local r_z_p = "**"
if `r(p_l)' < 0.001 local r_z_p = "***"
local r_z_p_star = "`r_z'`r_z_p'"

flexmat addrow, data(Practice Days,`r_t_p_star',`r_z_p_star') ///
dec(3) row(3) col(1) location(`next')

// Practiced Questions
ttest practice_count, by(counting_days)

local r_t = string(`r(t)', "%7.3f")
local r_t_p = ""
if `r(p_l)' < 0.05 local r_t_p = "*"
if `r(p_l)' < 0.01 local r_t_p = "**"
if `r(p_l)' < 0.001 local r_t_p = "***"
local r_t_p_star = "`r_t'`r_t_p'"

ranksum practice_count, by(counting_days)

local r_z = string(`r(z)', "%7.3f")
local r_z_p = ""
if `r(p_l)' < 0.05 local r_z_p = "*"
if `r(p_l)' < 0.01 local r_z_p = "**"
if `r(p_l)' < 0.001 local r_z_p = "***"
local r_z_p_star = "`r_z'`r_z_p'"

flexmat addrow, data(Practiced Questions,`r_t_p_star',`r_z_p_star') ///
dec(3) row(4) col(1) location(`next')

asdocx export, save(Between-instructor.tex)

gen student_dummy = 1
collapse (count) students = student_dummy, by(instructor counting_days)
list
```

Supplementary Note 5: R-Studio Notebook

R-Studio Notebook

Iman YeckehZaare, Paul Resnick

Set up the environment variables

```r
r = getOption("repos")
r["CRAN"] = "http://cran.us.r-project.org"
options(repos = r)

options(digits=10)
options(width = 60)
matrix(runif(100), ncol = 20)
```

```
##              [,1]         [,2]          [,3]         [,4]
## [1,] 0.7104477731 0.6419925222 0.24211762054 0.6260075069
## [2,] 0.7792001443 0.4385726771 0.02229636908 0.1285179122
## [3,] 0.8325256384 0.5347376242 0.30413200962 0.3783289725
## [4,] 0.7538444069 0.9595521928 0.65559673682 0.2228777106
## [5,] 0.8497848280 0.9580490945 0.21202429733 0.3764549871
##              [,5]         [,6]         [,7]          [,8]
## [1,] 0.1074214133 0.4682945770 0.4853850878 0.14949537092
## [2,] 0.1843663387 0.6462973822 0.2518510674 0.05067741754
## [3,] 0.7378473482 0.9332648739 0.1009575243 0.81863919576
## [4,] 0.3165216711 0.4686003122 0.4224106730 0.18482653820
## [5,] 0.5441502086 0.1946938743 0.2302389310 0.86603297433
##              [,9]        [,10]        [,11]        [,12]
## [1,] 0.6098281660 0.2175962005 0.7655011136 0.8028564421
## [2,] 0.7153934203 0.6611402703 0.7316111205 0.4699584555
## [3,] 0.4038865848 0.4166100090 0.9886743072 0.7276728281
## [4,] 0.9909409850 0.2714083039 0.0164509567 0.3436831876
## [5,] 0.7046374448 0.2201043051 0.4222608611 0.7777677630
##             [,13]         [,14]        [,15]        [,16]
## [1,] 0.1730189547 0.85856138729 0.9025488272 0.9047292694
## [2,] 0.6946046054 0.03632859769 0.2610253110 0.7369202108
## [3,] 0.6244405254 0.10061051953 0.4533374019 0.6961306066
## [4,] 0.8791738853 0.33839941467 0.8619445777 0.8943506009
## [5,] 0.4985356061 0.32022955571 0.9460747021 0.3301742116
##              [,17]        [,18]        [,19]        [,20]
## [1,] 0.09624565952 0.1696030572 0.4022572690 0.3495692557
## [2,] 0.50484146969 0.2602905561 0.2459233259 0.4859863138
## [3,] 0.06639569206 0.7758916798 0.4028832009 0.2173206504
## [4,] 0.77479309752 0.6005955737 0.8642588498 0.4217446416
## [5,] 0.56076382473 0.1853601558 0.9341650119 0.9030852565
```

Install and load the necessary libraries

```r
if(!require('dplyr')) {
  install.packages('dplyr')
  library('dplyr')
}
```

```
## Loading required package: dplyr

##
## Attaching package: 'dplyr'

## The following objects are masked from 'package:stats':
##
##     filter, lag

## The following objects are masked from 'package:base':
##
##     intersect, setdiff, setequal, union
```

```r
if(!require('emmeans')) {
  install.packages('emmeans')
  library('emmeans')
}
```

```
## Loading required package: emmeans

## Welcome to emmeans.
## Caution: You lose important information if you filter this package's results.
## See '? untidy'
```

```r
if(!require('ggplot2')) {
  install.packages('ggplot2')
  library('ggplot2')
}
```

```
## Loading required package: ggplot2
```

```r
if(!require('sjPlot')) {
  install.packages('sjPlot')
  library('sjPlot')
}
```

```
## Loading required package: sjPlot
```

```r
if(!require('sjstats')) {
  install.packages('sjstats')
  library('sjstats')
}
```

```
## Loading required package: sjstats
```

```r
if(!require('betareg')) {
  install.packages('betareg')
  library('betareg')
}
```

```
## Loading required package: betareg
```

```r
if(!require('vtable')) {
  install.packages('vtable')
```

```r
  library('vtable')
}
```

```
## Loading required package: vtable

## Loading required package: kableExtra

##
## Attaching package: 'kableExtra'

## The following object is masked from 'package:dplyr':
##
##     group_rows
```

```r
if(!require('car')) {
  install.packages('car')
  library('car')
}
```

```
## Loading required package: car

## Loading required package: carData

##
## Attaching package: 'car'

## The following object is masked from 'package:dplyr':
##
##     recode
```

```r
if(!require('texreg')) {
  install.packages('texreg')
  library('texreg')
}
```

```
## Loading required package: texreg

## Version:  1.39.4
## Date:     2024-07-23
## Author:   Philip Leifeld (University of Manchester)
##
## Consider submitting praise using the praise or praise_interactive functions.
## Please cite the JSS article in your publications -- see citation("texreg").
```

```r
if(!require('gridExtra')) {
  install.packages('gridExtra')
  library('gridExtra')
}
```

```
## Loading required package: gridExtra

##
## Attaching package: 'gridExtra'

## The following object is masked from 'package:dplyr':
##
##     combine
```

```r
if(!require('MASS')) {
  install.packages('MASS')
```

```r
  library('MASS')
}
```

```
## Loading required package: MASS

##
## Attaching package: 'MASS'

## The following object is masked from 'package:dplyr':
##
##     select
```

```r
if(!require('broom')) {
  install.packages('broom')
  library('broom')
}
```

```
## Loading required package: broom

##
## Attaching package: 'broom'

## The following object is masked from 'package:sjstats':
##
##     bootstrap
```

```r
if(!require('kableExtra')) {
  install.packages('kableExtra')
  library('kableExtra')
}
if(!require('xtable')) {
  install.packages('xtable')
  library('xtable')
}
```

```
## Loading required package: xtable
```

```r
if(!require('tidyr')) {
  install.packages('tidyr')
  library('tidyr')
}
```

```
## Loading required package: tidyr

##
## Attaching package: 'tidyr'

## The following object is masked from 'package:texreg':
##
##     extract
```

```r
if(!require('scales')) {
  install.packages('scales')
  library('scales')
}
```

```
## Loading required package: scales
```

```r
if(!require('coin')) {
  install.packages('coin')
```

```
  library('coin')
}
```

```
## Loading required package: coin

## Loading required package: survival

##
## Attaching package: 'coin'

## The following object is masked from 'package:scales':
##
##     pvalue
```

Power Analysis

Simulate the dataset

```
# We want 1000 simulations
n_sims <- 1000
p_vals <- c()
# This vector will contain the power for each sample-size (it needs the initial 0 for the while-loop to
power_at_n <- c(0)
# Sample-size and start at 100 as we can be pretty sure this will not suffice for such a small effect
n <- 50
# By which step size should be increased
n_increase <- 5
i <- 2
power_crit <- 0.80
alpha <- 0.05
while(power_at_n[i-1] < power_crit){
  for(sim in 1:n_sims){
    # Total number of students
    n_students <- n
    # Total number of conditions
    n_conditions <- 2
    # The two conditions
    condition <- c("Counting Days", "Counting Questions")
    # The student indicators
    student <- factor(1:n_students)
    # We assume the GPA is Z-score transformed, so it has a standard normal distribution
    # with mean 0.0
    GPA <- rnorm(n_students, 0.0, 1)
    # The condition that each student is randomly assigned to:
    Spacing <- factor(sample(condition, n_students, replace=T))
    # We assume the final score for each student has a normal distribution
    # with a mean of 80%; be affected by the spacing condition and GPA; and the
    # interaction of GPA and the Spacing condition. We assume that:
    # 1) The Counting Days, compared to the Counting Questions condition,
    #    increases the final exam score by 5%.
    # 2) Every standard deviation increase in GPA, increases the final exam score by 10%
    # 3) Every standard deviation increase in GPA, for those under Counting Days, does
    # not increase the final exam score by 10%
    # 4) There is a normal variation (error term) in final observations with a mean of 0
```

```
    # and standard deviation of 15%
    final <- 80 +
      5 * ifelse(Spacing == "Counting Days", 1, 0) +
      10 * GPA * ifelse(Spacing == "Counting Questions", 1, 0) +
      rnorm(n_students, 0, 15)
    # Because the final exam score should be bounded as a percentage, we divide the
    # final exam scores by their maximum value and analyze them as a beta distribution
    # of proportions in range (0, 1).
    minF <- min(final)
    maxF <- max(final)
    final <- (final + ifelse(minF > 0, 0, 0.1 - minF)) /
      (maxF + 0.1 + ifelse(minF > 0, 0, 0.1 - minF))
    covars <- data.frame(student=student,
      Spacing=relevel(Spacing, ref = "Counting Questions"),
      GPA=GPA,
      final=final)
    # Fit the model with the interaction
    beta_model_int <- betareg(final ~ Spacing + GPA + Spacing:GPA, data=covars)
    # Put the p-values of the interaction effect in a list
    p_vals[sim] <- summary(beta_model_int)$coefficients$mean[4, 4]
  }
  print(n)
  # Check power (i.e. proportion of p-values that are smaller than alpha-level of 0.05)
  power_at_n[i] <- mean(p_vals < alpha)
  names(power_at_n)[i] <- n
  # Increase sample-size by 100 for low-resolution testing first
  n <- n + n_increase
  # Increase index of the while-loop by 1 to save power and Cohens d to vector
  i <- i + 1
}
```

```
## [1] 50
## [1] 55
## [1] 60
## [1] 65
## [1] 70
## [1] 75
## [1] 80
## [1] 85
```

Plot the power analysis

```
# Delete first 0 from the vector
power_at_n <- power_at_n[-1]
plot(as.numeric(names(power_at_n)), power_at_n, xlab = "Number of students",
  ylab = "Power", ylim = c(0,1), axes = TRUE)
abline(h = power_crit, col = "red")
```

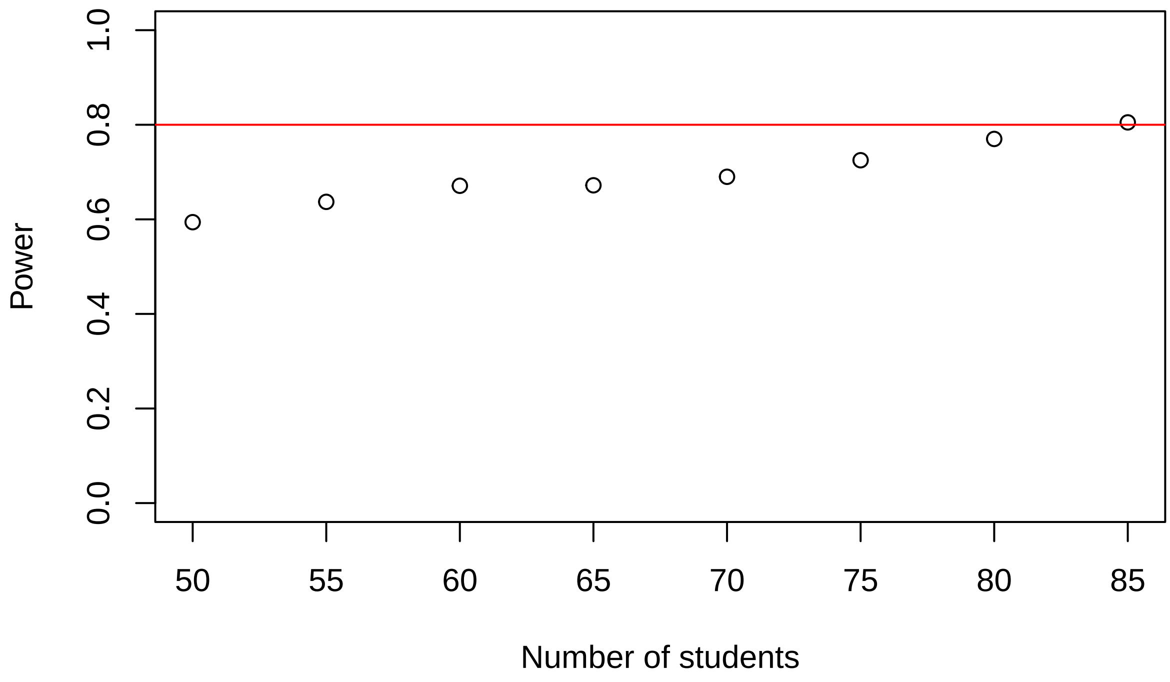

Analyze the Real Experiment Data

Load the CSV dataset

```
all_data <- read.csv("CountingDaysFourSemesterData.csv")
all_data$User_ID <- factor(all_data$User_ID)
all_data$Term <- factor(all_data$Term)
all_data$PracticeDate <- as.Date(all_data$PracticeDate)
all_data$PracticeMinutes <- all_data$Duration / 60
all_data$Q <- as.numeric(all_data$Q)
all_data$GradingType <- factor(all_data$GradingType)
all_data$Final <- as.numeric(all_data$Final)
all_data$Final_0_1 <- as.numeric(all_data$Final_0_1)
all_data$GPA <- as.numeric(all_data$GPA)
all_data$Sex <- factor(all_data$Sex)
all_data$Male <- factor(all_data$Male)
all_data$Female <- factor(all_data$Female)
all_data$Ethnicity <- factor(all_data$Ethnicity)
all_data$White <- factor(all_data$White)
all_data$Asian <- factor(all_data$Asian)
all_data$NonWhiteOrAsian <- factor(all_data$NonWhiteOrAsian)
all_data$NonNativeEnglish <- factor(all_data$NonNativeEnglish)
all_data$AcademicLevel <- factor(all_data$AcademicLevel)
all_data$Freshman <- factor(all_data$Freshman)
all_data$Junior <- factor(all_data$Junior)
all_data$Sophomore <- factor(all_data$Sophomore)
all_data$Senior <- factor(all_data$Senior)
```

Student-level Analysis

Table 2

Apply the data inclusion criteria to generate the student funnel

Only consider the data collected in the experiment semester, Fall 2018.

```
# {table:CountingDays_Participant_Funnel}
Fall2018_data <- subset(all_data, Term == "FA 2018")
Fall2018_data$Spacing = relevel(factor(Fall2018_data$Spacing),
                                ref = "Counting Questions")
```

Generate the student-level dataframe. Each row should represent a single student.

```
student_data <- Fall2018_data[Fall2018_data$Q > 0, ] %>%
  group_by(User_ID) %>%
  summarize(
    Spacing = Spacing[1],
    GradingType = GradingType[1],
    Final = Final[1],
    Final_0_1 = Final_0_1[1],
    GPA = GPA[1],
    Sex = Sex[1],
    Male = Male[1],
    Female = Female[1],
    Ethnicity = Ethnicity[1],
    White = White[1],
    Asian = Asian[1],
    NonWhiteOrAsian = NonWhiteOrAsian[1],
    NonNativeEnglish = NonNativeEnglish[1],
    Freshman = Freshman[1],
    Junior = Junior[1],
    Sophomore = Sophomore[1],
    Senior = Senior[1],
    AcademicLevel = AcademicLevel[1],
    Practice_Count = n(),
    Practice_Time = sum(PracticeMinutes) / 60,
    Practice_Days = n_distinct(PracticeDate))
```

The GPA field was populated from an inner join query with student data from the registrar. GPA field is blank when the student was not registered for this course in this semester (i.e., TAs and other system testers).

```
sumtable(student_data, vars = c("Spacing"), out="return",
title='Randomization of All students', summ=c('notNA(x)', 'mean(x)'),
summ.names=c('Number','Percent'))
```

```
## Warning in sumtable(student_data, vars = c("Spacing"), out = "return", title = "Randomization of All
## Beware combining factors with a custom summ unless factor.numeric = TRUE.
```

```
##                  Variable Number Percent
## 1                 Spacing    199
## 2 ... Counting Questions     89     45%
## 3      ... Counting Days    110     55%
```

```
withGPA_data <- subset(student_data, !is.na(GPA) & GPA != 0)
sumtable(withGPA_data, vars = c("Spacing"), out="return",
title='Randomization of All Students', summ=c('notNA(x)', 'mean(x)'),
```

```r
summ.names=c('Number','Percent'))
```

```
## Warning in sumtable(withGPA_data, vars = c("Spacing"), out = "return", title = "Randomization of All
## Beware combining factors with a custom summ unless factor.numeric = TRUE.
```

```
##                    Variable Number Percent
## 1                   Spacing    159
## 2 ... Counting Questions     70     44%
## 3      ... Counting Days     89     56%
```

After excluding those who have no GPA, a few Freshmen remained. We treat them as Sophomores because they had taken courses in the previous semester.

```r
withGPA_data$Sophomore <- factor(
ifelse(withGPA_data$Freshman == 1 | withGPA_data$Sophomore == 1, 1, 0))
withGPA_data$AcademicLevel <- factor(
ifelse(withGPA_data$Freshman == 1 | withGPA_data$Sophomore == 1, "Sophomore",
withGPA_data$AcademicLevel))
```

We exclude those students who did not take the final exam; i.e., withdrew the course (at the end of the semester, after paying the tuition).

```r
took_final_data <- subset(withGPA_data, !is.na(Final) & Final != 0)
sumtable(took_final_data, vars = c("Spacing"), out="return",
        title='Randomization of Students Who Took Final Exam',
        summ=c('notNA(x)', 'mean(x)'), summ.names=c('Number','Percent'))
```

```
## Warning in sumtable(took_final_data, vars = c("Spacing"), out = "return", : Factor variables ignore
## Beware combining factors with a custom summ unless factor.numeric = TRUE.
```

```
##                    Variable Number Percent
## 1                   Spacing    157
## 2 ... Counting Questions     68     43%
## 3      ... Counting Days     89     57%
```

We exclude those students who took the course for purposes other than getting a letter grade, such as auditing.

```r
data <- subset(took_final_data, GradingType == "Graded")
passFailData <- subset(took_final_data, GradingType != "Graded")
sumtable(data, vars = c("Spacing"), out="return",
        title='Randomization of Students Who Took Final Exam for a Letter Grade',
        summ=c('notNA(x)', 'mean(x)'), summ.names=c('Number','Percent'))
```

```
## Warning in sumtable(data, vars = c("Spacing"), out = "return", title = "Randomization of Students Who
## Beware combining factors with a custom summ unless factor.numeric = TRUE.
```

```
##                    Variable Number Percent
## 1                   Spacing    143
## 2 ... Counting Questions     60     42%
## 3      ... Counting Days     83     58%
```

Tables 3

Summary statistics of both input and output variables and randomization check.

```r
# {table:CountingDays_Demographics}
sumtable(data, vars = c("Final", "Practice_Days", "Practice_Time", "Practice_Count",
                        "GPA", "Female", "Ethnicity", "NonWhiteOrAsian", "AcademicLevel",
```

```
                        "NonNativeEnglish"), group="Spacing", group.long = TRUE,
        group.test = TRUE, out="return",
        summ=c('min(x)', 'pctile(x)[50]', 'max(x)', 'mean(x)', 'sd(x)'),
        summ.names=c('Min', 'Median', 'Max', 'Mean', 'Standard Deviation'))
```

```
## Warning in sumtable(data, vars = c("Final",
## "Practice_Days", "Practice_Time", : group.test is
## incompatible with group.long == TRUE and will be ignored.

## Warning in sumtable(data, vars = c("Final", "Practice_Days", "Practice_Time", : Factor variables igno
## Beware combining factors with a custom summ unless factor.numeric = TRUE.

##                        Variable Min Median Max Mean
## 1  Spacing: Counting Questions
## 2                         Final  49     83 100   82
## 3                 Practice_Days   4     17  40   17
## 4                 Practice_Time 3.7    9.8  19  9.7
## 5                Practice_Count 170    401 444  392
## 6                           GPA 1.9    3.5   4  3.4
## 7                        Female  60
## 8                         ... 0  24    40%
## 9                         ... 1  36    60%
## 10                    Ethnicity  60
## 11          ... African American   1     2%
## 12                    ... Asian  12    20%
## 13                 ... Hispanic   5     8%
## 14                    ... Other   5     8%
## 15                    ... White  37    62%
## 16               NonWhiteOrAsian  60
## 17                        ... 0  49    82%
## 18                        ... 1  11    18%
## 19                 AcademicLevel  60
## 20                        ... 2  10    17%
## 21                        ... 3  11    18%
## 22                ... Sophomore  39    65%
## 23              NonNativeEnglish  60
## 24                        ... 0  41    68%
## 25                        ... 1  19    32%
## 26
## 27      Spacing: Counting Days
## 28                         Final  36     87 100   85
## 29                 Practice_Days  16     39  48   39
## 30                 Practice_Time 3.1    8.6  21  9.1
## 31                Practice_Count 190    412 662  415
## 32                           GPA   2    3.6   4  3.5
## 33                        Female  83
## 34                        ... 0  29    35%
## 35                        ... 1  54    65%
## 36                    Ethnicity  83
## 37          ... African American   0     0%
## 38                    ... Asian  21    25%
## 39                 ... Hispanic   2     2%
## 40                    ... Other  10    12%
## 41                    ... White  50    60%
## 42               NonWhiteOrAsian  83
```

```
## 43                          ... 0  71    86%
## 44                          ... 1  12    14%
## 45               AcademicLevel  83
## 46                          ... 2  17    20%
## 47                          ... 3  15    18%
## 48               ... Sophomore  51    61%
## 49            NonNativeEnglish  83
## 50                          ... 0  66    80%
## 51                          ... 1  17    20%
##    Standard Deviation
## 1
## 2                  12
## 3                 7.4
## 4                 3.3
## 5                  46
## 6                0.49
## 7
## 8
## 9
## 10
## 11
## 12
## 13
## 14
## 15
## 16
## 17
## 18
## 19
## 20
## 21
## 22
## 23
## 24
## 25
## 26
## 27
## 28                 10
## 29                  5
## 30                3.3
## 31                 62
## 32                0.4
## 33
## 34
## 35
## 36
## 37
## 38
## 39
## 40
## 41
## 42
## 43
## 44
```

```
## 45
## 46
## 47
## 48
## 49
## 50
## 51
```

```r
sumtable(data, vars = c("Final", "Practice_Days", "Practice_Time", "Practice_Count",
                        "GPA", "Female", "Ethnicity", "NonWhiteOrAsian", "AcademicLevel",
                        "NonNativeEnglish"), group="Spacing", group.test = TRUE,
        out="return")
```

```
##                Variable                  N Mean   SD
## 1               Spacing Counting Questions
## 2                 Final                 60   82   12
## 3         Practice_Days                 60   17  7.4
## 4         Practice_Time                 60  9.7  3.3
## 5        Practice_Count                 60  392   46
## 6                   GPA                 60  3.4 0.49
## 7                Female                 60
## 8                 ... 0                 24  40%
## 9                 ... 1                 36  60%
## 10            Ethnicity                 60
## 11 ... African American                  1   2%
## 12           ... Asian                  12  20%
## 13        ... Hispanic                   5   8%
## 14           ... Other                   5   8%
## 15           ... White                  37  62%
## 16      NonWhiteOrAsian                 60
## 17                ... 0                 49  82%
## 18                ... 1                 11  18%
## 19        AcademicLevel                 60
## 20                ... 2                 10  17%
## 21                ... 3                 11  18%
## 22        ... Sophomore                 39  65%
## 23     NonNativeEnglish                 60
## 24                ... 0                 41  68%
## 25                ... 1                 19  32%
##                N Mean  SD         Test
## 1  Counting Days
## 2             83   85  10    F=3.954**
## 3             83   39   5 F=417.631***
## 4             83  9.1 3.3      F=0.954
## 5             83  415  62    F=5.664**
## 6             83  3.5 0.4     F=2.742*
## 7             83                X2=0.196
## 8             29  35%
## 9             54  65%
## 10            83                X2=4.774
## 11             0   0%
## 12            21  25%
## 13             2   2%
## 14            10  12%
## 15            50  60%
```

```
## 16              83              X2=0.154
## 17              71  86%
## 18              12  14%
## 19              83               X2=0.34
## 20              17  20%
## 21              15  18%
## 22              51  61%
## 23              83              X2=1.757
## 24              66  80%
## 25              17  20%
```

```r
sumtable(data, vars = c("Final", "Practice_Days", "Practice_Time", "Practice_Count",
                        "GPA", "Female", "Ethnicity", "NonWhiteOrAsian", "AcademicLevel",
                        "NonNativeEnglish"), out="return",
        summ=c('min(x)', 'pctile(x)[50]', 'max(x)', 'mean(x)', 'sd(x)'),
        summ.names=c('Min', 'Median', 'Max', 'Mean', 'Standard Deviation'))
```

```
## Warning in sumtable(data, vars = c("Final", "Practice_Days", "Practice_Time", : Factor variables igno
## Beware combining factors with a custom summ unless factor.numeric = TRUE.
```

```
##                Variable Min Median Max Mean
## 1                 Final  36     86 100   84
## 2         Practice_Days   4     34  48   30
## 3         Practice_Time 3.1    8.8  21  9.4
## 4        Practice_Count 170    402 662  406
## 5                   GPA 1.9    3.5   4  3.4
## 6                Female 143
## 7                 ... 0  53    37%
## 8                 ... 1  90    63%
## 9             Ethnicity 143
## 10 ... African American   1     1%
## 11            ... Asian  33    23%
## 12         ... Hispanic   7     5%
## 13            ... Other  15    10%
## 14            ... White  87    61%
## 15      NonWhiteOrAsian 143
## 16                ... 0 120    84%
## 17                ... 1  23    16%
## 18        AcademicLevel 143
## 19                ... 2  27    19%
## 20                ... 3  26    18%
## 21        ... Sophomore  90    63%
## 22     NonNativeEnglish 143
## 23                ... 0 107    75%
## 24                ... 1  36    25%
##    Standard Deviation
## 1                  11
## 2                  12
## 3                 3.3
## 4                  57
## 5                0.44
## 6
## 7
## 8
## 9
```

```
## 10
## 11
## 12
## 13
## 14
## 15
## 16
## 17
## 18
## 19
## 20
## 21
## 22
## 23
## 24
```

Table 4:

Summary statistics and randomization check of prior GPA in the within-class experiment.

```
# {CountingDays:priorGPA}
# Compute summary statistics for Prior GPA
summary_stats <- data %>%
  group_by(Group = Spacing) %>%
  summarise(
    Variable = "Prior GPA",
    Min = min(GPA, na.rm = TRUE),
    Median = median(GPA, na.rm = TRUE),
    Max = max(GPA, na.rm = TRUE),
    Mean = mean(GPA, na.rm = TRUE),
    SD = sd(GPA, na.rm = TRUE),
    .groups = 'drop'
  )

# Perform ANOVA to get F-statistic for Prior GPA between groups
anova_result <- aov(GPA ~ Spacing, data = data)
anova_summary <- summary(anova_result)
F_value <- anova_summary[[1]]$`F value`[1]
F_value_formatted <- sprintf("%.3f", F_value)

# Format the summary statistics to match the desired decimal places
summary_stats_formatted <- summary_stats %>%
  mutate(
    Min = sprintf("%.3f", Min),
    Median = sprintf("%.3f", Median),
    Max = sprintf("%.3f", Max),
    Mean = sprintf("%.3f", Mean),
    SD = sprintf("%.3f", SD)
  )

# Prepare the LaTeX table code
latex_table <- "\\begin{table*}[!htp]
\\centering
\\ra{1.0}
```

```
\\begin{tabular}{@{}p{1.3cm}p{1.51cm}rrrrrl@{}}
\\toprule
& Group & Min & Median & Max & Mean & SD & Test \\\\ \\midrule
"

# Since we have two groups, we use multirow of 2
latex_table <- paste0(latex_table,
"\\multirow[c.]{4}{1.3cm}{\\textbf{Prior GPA}} & ", summary_stats_formatted$Group[2], " & ",
summary_stats_formatted$Min[2], "& ", summary_stats_formatted$Median[2], "& ",
summary_stats_formatted$Max[2], "& ", summary_stats_formatted$Mean[2], "& ",
summary_stats_formatted$SD[2], " & \\multirow{4}{*}{\\begin{tabular}[c]{@{}l@{}}F=\\\\", F_value_format
" & ", summary_stats_formatted$Group[2], " & ",
summary_stats_formatted$Min[1], "& ", summary_stats_formatted$Median[1], "& ",
summary_stats_formatted$Max[1], "& ", summary_stats_formatted$Mean[1], "& ",
summary_stats_formatted$SD[1], " & \\\\ \n")

# Finish the table
latex_table <- paste0(latex_table,
"\\end{tabular}
\\caption{Summary statistics and randomization check of prior GPA in the within-class experiment.}
\\label{CountingDays:priorGPA}
\\end{table*}")

# Output the LaTeX code
cat(latex_table)
```

```
## \begin{table*}[!htp]
## \centering
## \ra{1.0}
## \begin{tabular}{@{}p{1.3cm}p{1.51cm}rrrrrl@{}}
## \toprule
## & Group & Min & Median & Max & Mean & SD & Test \\ \midrule
## \multirow[c.]{4}{1.3cm}{\textbf{Prior GPA}} & Counting Days & 1.967& 3.626& 3.954& 3.482& 0.404 & \mu
##  & Counting Days & 1.903& 3.469& 4.000& 3.358& 0.487 & \\
## \end{tabular}
## \caption{Summary statistics and randomization check of prior GPA in the within-class experiment.}
## \label{CountingDays:priorGPA}
## \end{table*}
```

Table 5:

Summary statistics of the outcome variables in the within-class experiment.

```
# {table:CountingDays_Within_Class_Experiment_Outcome_Summary_Statistics}
# Create a mapping of variable names to labels
variable_labels <- c(
  "Final" = "Final Exam Score (in \\%)",
  "Practice_Days" = "Practice Days",
  "Practice_Count" = "Practiced Questions"
)

# List of variables to summarize
variables_to_summarize <- c("Final", "Practice_Days", "Practice_Count")
```

```r
# Initialize an empty data frame to store the summary statistics
summary_stats <- data.frame()

# Compute summary statistics for each variable
for (var in variables_to_summarize) {
  stats <- data %>%
    group_by(Group = Spacing) %>%
    summarise(
      Variable = variable_labels[var],
      Min = min(.data[[var]], na.rm = TRUE),
      Median = median(.data[[var]], na.rm = TRUE),
      Max = max(.data[[var]], na.rm = TRUE),
      Mean = mean(.data[[var]], na.rm = TRUE),
      SD = sd(.data[[var]], na.rm = TRUE),
      .groups = 'drop'
    )
  summary_stats <- bind_rows(summary_stats, stats)
}

# Set the order of Variables and Groups
summary_stats <- summary_stats %>%
  mutate(
    Variable = factor(Variable, levels = c(
      "Final Exam Score (in \\%)",
      "Practice Days",
      "Practiced Questions"
    )),
    Group = factor(Group, levels = c("Counting Days", "Counting Questions"))
  ) %>%
  arrange(Variable, Group)

# Function to format numbers according to the desired format
format_stat <- function(value, variable, stat_name) {
  if (variable == "Final Exam Score (in \\%)") {
    if (stat_name %in% c("Min", "Median", "Mean", "SD")) {
      # One decimal place
      return(sprintf("%.1f", value))
    } else if (stat_name == "Max") {
      # No decimal places
      return(sprintf("%.0f", value))
    }
  } else if (variable == "Practice Days") {
    if (stat_name %in% c("Mean", "SD")) {
      return(sprintf("%.1f", value))
    } else {
      return(sprintf("%.0f", value))
    }
  } else if (variable == "Practiced Questions") {
    if (stat_name %in% c("Mean", "SD")) {
      return(sprintf("%.1f", value))
    } else {
      return(sprintf("%.0f", value))
    }
```

```r
  }
  return(as.character(value))
}

# Apply the formatting to the summary_stats data frame
summary_stats_formatted <- summary_stats %>%
  rowwise() %>%
  mutate(
    Min = format_stat(Min, Variable, "Min"),
    Median = format_stat(Median, Variable, "Median"),
    Max = format_stat(Max, Variable, "Max"),
    Mean = format_stat(Mean, Variable, "Mean"),
    SD = format_stat(SD, Variable, "SD")
  ) %>%
  ungroup()

# Prepare the LaTeX code manually
latex_table <- "\\begin{table*}[!htp]
\\centering
\\ra{1.3}
\\begin{tabular}{@{}p{2.2cm}p{1.51cm}rrrrr@{}}
\\toprule
& Group & Min & Median & Max & Mean & SD \\\\ \\midrule
"

variables <- unique(summary_stats_formatted$Variable)

for (var in variables) {
  data_sub <- summary_stats_formatted %>% filter(Variable == var)
  # Start the multirow for the variable
  latex_table <- paste0(latex_table, "\\multirow[c.]{4}{2.2cm}{\\textbf{", var, "}} ")
  for (i in 1:nrow(data_sub)) {
    row <- data_sub[i, ]
    if (i == 1) {
      # First row with multirow
      latex_table <- paste0(latex_table, "& ", row$Group, " & ", row$Min, "& ", row$Median, "& ", row$Ma
    } else {
      # Second row without multirow
      latex_table <- paste0(latex_table, "& ", row$Group, " & ", row$Min, "& ", row$Median, "& ", row$Ma
    }
  }
  # Add \hline after each variable block except the last one
  if (var != tail(variables, n = 1)) {
    latex_table <- paste0(latex_table, "\\hline \n")
  }
}

# Finish the table
latex_table <- paste0(latex_table, "\\bottomrule \\\\
\\end{tabular}
\\caption{Summary statistics of the outcome variables in the within-class experiment.}
\\label{table:CountingDays_Within_Class_Experiment_Outcome_Summary_Statistics}
\\end{table*}")
```

```
# Output the LaTeX code
cat(latex_table)
```

```
## \begin{table*}[!htp]
## \centering
## \ra{1.3}
## \begin{tabular}{@{}p{2.2cm}p{1.51cm}rrrrr@{}}
## \toprule
## & Group & Min & Median & Max & Mean & SD \\ \midrule
## \multirow[c.]{4}{2.2cm}{\textbf{Final Exam Score (in \%)}} & Counting Days & 35.6& 87.3& 100& 85.3& 1
## & Counting Questions & 49.1& 83.3& 100& 81.7& 11.6 \\
## \hline
## \multirow[c.]{4}{2.2cm}{\textbf{Practice Days}} & Counting Days & 16& 39& 48& 38.5& 5.0 \\ \cline{2-7
## & Counting Questions & 4& 17& 40& 17.3& 7.4 \\
## \hline
## \multirow[c.]{4}{2.2cm}{\textbf{Practiced Questions}} & Counting Days & 190& 412& 662& 415.0& 61.7 \\
## & Counting Questions & 170& 401& 444& 392.5& 46.3 \\
## \bottomrule \\
## \end{tabular}
## \caption{Summary statistics of the outcome variables in the within-class experiment.}
## \label{table:CountingDays_Within_Class_Experiment_Outcome_Summary_Statistics}
## \end{table*}
```

Table C1:

Summary statistics of the numeric variables in the within-class experiment, only for students who took the course Pass/Fail. Due to the small number of observations, we do not report statistical tests between the two conditions.

```
# {table:CountingDays_Summary_Statistics_With_Pass_Fail}
# Create a mapping of variable names to labels
variable_labels <- c(
  "Final" = "Final Exam Score (in \\%)",
  "Practice_Days" = "Practice Days",
  "Practice_Count" = "Practiced Questions"
)

# List of variables to summarize
variables_to_summarize <- c("Final", "Practice_Days", "Practice_Count")

# Initialize an empty data frame to store the summary statistics
summary_stats <- data.frame()

# Compute summary statistics for each variable
for (var in variables_to_summarize) {
  stats <- passFailData %>%
    group_by(Group = Spacing) %>%
    summarise(
      Variable = variable_labels[var],
      Min = min(.data[[var]], na.rm = TRUE),
      Median = median(.data[[var]], na.rm = TRUE),
      Max = max(.data[[var]], na.rm = TRUE),
      Mean = mean(.data[[var]], na.rm = TRUE),
      SD = sd(.data[[var]], na.rm = TRUE),
```

```r
      .groups = 'drop'
    )
  summary_stats <- bind_rows(summary_stats, stats)
}

# Set the order of Variables and Groups
summary_stats <- summary_stats %>%
  mutate(
    Variable = factor(Variable, levels = c(
      "Final Exam Score (in \\%)",
      "Practice Days",
      "Practiced Questions"
    )),
    Group = factor(Group, levels = c("Counting Days", "Counting Questions"))
  ) %>%
  arrange(Variable, Group)

# Function to format numbers according to the desired format
format_stat <- function(value, variable, stat_name) {
  if (variable == "Final Exam Score (in \\%)") {
    if (stat_name %in% c("Min", "Median", "Mean", "SD")) {
      # One decimal place
      return(sprintf("%.1f", value))
    } else if (stat_name == "Max") {
      # No decimal places
      return(sprintf("%.0f", value))
    }
  } else if (variable == "Practice Days") {
    if (stat_name %in% c("Mean", "SD")) {
      return(sprintf("%.1f", value))
    } else {
      return(sprintf("%.0f", value))
    }
  } else if (variable == "Practiced Questions") {
    if (stat_name %in% c("Mean", "SD")) {
      return(sprintf("%.1f", value))
    } else {
      return(sprintf("%.0f", value))
    }
  }
  return(as.character(value))
}

# Apply the formatting to the summary_stats data frame
summary_stats_formatted <- summary_stats %>%
  rowwise() %>%
  mutate(
    Min = format_stat(Min, Variable, "Min"),
    Median = format_stat(Median, Variable, "Median"),
    Max = format_stat(Max, Variable, "Max"),
    Mean = format_stat(Mean, Variable, "Mean"),
    SD = format_stat(SD, Variable, "SD")
  ) %>%
```

```
  ungroup()

# Prepare the LaTeX code manually
latex_table <- "\\begin{table*}[!htp]
\\centering
\\ra{1.3}
\\begin{tabular}{@{}p{2.2cm}p{1.51cm}rrrrr@{}}
\\toprule
& Group & Min & Median & Max & Mean & SD \\\\ \\midrule
"

variables <- unique(summary_stats_formatted$Variable)

for (var in variables) {
  data_sub <- summary_stats_formatted %>% filter(Variable == var)
  # Start the multirow for the variable
  latex_table <- paste0(latex_table, "\\multirow[c.]{4}{2.2cm}{\\textbf{", var, "}} ")
  for (i in 1:nrow(data_sub)) {
    row <- data_sub[i, ]
    if (i == 1) {
      # First row with multirow
      latex_table <- paste0(latex_table, "& ", row$Group, " & ", row$Min, "& ", row$Median, "& ", row$Ma
    } else {
      # Second row without multirow
      latex_table <- paste0(latex_table, "& ", row$Group, " & ", row$Min, "& ", row$Median, "& ", row$Ma
    }
  }
  # Add \hline after each variable block except the last one
  if (var != tail(variables, n = 1)) {
    latex_table <- paste0(latex_table, "\\hline \n")
  }
}

# Finish the table
latex_table <- paste0(latex_table, "\\bottomrule \\\\
\\end{tabular}
\\caption{Summary statistics of the numeric variables in the within-class experiment, only for students
\\label{table:CountingDays_Summary_Statistics_With_Pass_Fail}
\\end{table*}")

# Output the LaTeX code
cat(latex_table)
```

```
## \begin{table*}[!htp]
## \centering
## \ra{1.3}
## \begin{tabular}{@{}p{2.2cm}p{1.51cm}rrrrr@{}}
## \toprule
## & Group & Min & Median & Max & Mean & SD \\ \midrule
## \multirow[c.]{4}{2.2cm}{\textbf{Final Exam Score (in \%)}} & Counting Days & 51.5& 72.4& 83& 69.7& 1
## & Counting Questions & 16.0& 75.5& 96& 70.1& 25.8 \\
## \hline
## \multirow[c.]{4}{2.2cm}{\textbf{Practice Days}} & Counting Days & 27& 40& 44& 37.7& 6.0 \\ \cline{2-
## & Counting Questions & 1& 14& 22& 12.9& 7.3 \\
```

```
## \hline
## \multirow[c.]{4}{2.2cm}{\textbf{Practiced Questions}} & Counting Days & 287& 439& 574& 445.0& 94.2 \\
## & Counting Questions & 2& 400& 412& 313.8& 154.0 \\
## \bottomrule \\
## \end{tabular}
## \caption{Summary statistics of the numeric variables in the within-class experiment, only for studen
## \label{table:CountingDays_Summary_Statistics_With_Pass_Fail}
## \end{table*}
```

Calculate the percentiles of GPA.

```
quantile(data$GPA, probs = seq(.1, .9, by = .1))
```

```
##    10%    20%    30%    40%    50%    60%    70%    80%
## 2.8926 3.1214 3.2912 3.4448 3.5480 3.6376 3.7248 3.7926
##    90%
## 3.8774
```

Specify the treatment and control colors for all the diagrams.

```
Counting_Days_Group_Colors <- c("Counting Days" = "tomato1",
"Counting Questions" = "turquoise3")
```

Table 6

T-test and Two-sample Wilcoxon rank-sum (Mann–Whitney) test results for the within-class experiment. (*** $p < 0.001$; ** $p < 0.01$; * $p < 0.05$).

```
# {tab:within-course-ttest}
# Reverse the levels of Spacing for the t.test.
data$Spacing_R <- factor(data$Spacing, levels = c("Counting Questions", "Counting Days"))

# Initialize a data frame to store results
results <- data.frame(
  Variable = c("Final Exam Score", "Practiced Days", "Practiced Questions"),
  T_test = character(3),
  Wilcoxon_test = character(3),
  stringsAsFactors = FALSE
)

# Function to format significance stars
get_significance_stars <- function(p_value) {
  if (p_value < 0.001) {
    return("***")
  } else if (p_value < 0.01) {
    return("**")
  } else if (p_value < 0.05) {
    return("*")
  } else {
    return("")
  }
}

# Perform tests for each variable
variables <- list(
  list(name = "Final Exam Score", var = "Final"),
```

```r
  list(name = "Practiced Days", var = "Practice_Days"),
  list(name = "Practiced Questions", var = "Practice_Count")
)

for (i in seq_along(variables)) {
  var_name <- variables[[i]]$name
  var <- variables[[i]]$var

  # T-test
  t_test <- t.test(as.formula(paste(var, "~ Spacing_R")), data = data, alternative = "less", var.equal =
  t_value <- t_test$statistic
  p_value_t <- t_test$p.value
  stars_t <- get_significance_stars(p_value_t)
  t_stat_formatted <- paste0(sprintf("%.3f", t_value), stars_t)

  # Wilcoxon test using 'coin' package
  wilcox_test_result <- wilcox_test(
    as.formula(paste(var, "~ Spacing_R")),
    data = data,
    alternative = "less",
    distribution = "asymptotic"
  )
  z_value <- statistic(wilcox_test_result, type = "standardized")
  p_value_w <- pvalue(wilcox_test_result)
  stars_w <- get_significance_stars(p_value_w)
  wilcox_stat_formatted <- paste0(sprintf("%.3f", abs(z_value)), stars_w)

  # Store results
  results$T_test[results$Variable == var_name] <- t_stat_formatted
  results$Wilcoxon_test[results$Variable == var_name] <- wilcox_stat_formatted
}

# Prepare the LaTeX table code
latex_table <- "\\begin{table*}[!htp]
\\centering
\\ra{1.6}
\\begin{tabular}{@{}p{3.7cm}p{3.1cm}p{4.3cm}@{}}
\\toprule
& Two-sample t-test with equal variances & Two-sample Wilcoxon rank-sum (Mann--Whitney) test \\\\ \\mid
"

for (i in 1:nrow(results)) {
  latex_table <- paste0(
    latex_table,
    "\\textbf{", results$Variable[i], "}&",
    results$T_test[i], "&",
    results$Wilcoxon_test[i], " \\\\ \n"
  )
}

latex_table <- paste0(
  latex_table,
  "\\bottomrule \\\\
```

```
\\end{tabular}
\\caption{T-test and Two-sample Wilcoxon rank-sum (Mann--Whitney) test results for the within-class expe
\\label{tab:within-course-ttest}
\\end{table*}"
)

# Output the LaTeX code
cat(latex_table)
```

```
## \begin{table*}[!htp]
## \centering
## \ra{1.6}
## \begin{tabular}{@{}p{3.7cm}p{3.1cm}p{4.3cm}@{}}
## \toprule
## & Two-sample t-test with equal variances & Two-sample Wilcoxon rank-sum (Mann--Whitney) test \\ \mid
## \textbf{Final Exam Score}&-1.988*&1.968* \\
## \textbf{Practiced Days}&-20.436***&9.767*** \\
## \textbf{Practiced Questions}&-2.380**&2.745** \\
## \bottomrule \\
## \end{tabular}
## \caption{T-test and Two-sample Wilcoxon rank-sum (Mann--Whitney) test results for the within-class e
## \label{tab:within-course-ttest}
## \end{table*}
```

Table 7 - Column 2 - Regression results with controls of the within-class experiment. Dependent variables are specified in column headers.

```
# {table:CountingDays_Within_Class_Experiment_Regression_Results_with_controls}
Final_Model <- betareg(Final_0_1 ~ Spacing * GPA + Female + Asian + NonWhiteOrAsian +
                      Junior + Senior + NonNativeEnglish, data = data)
summary(Final_Model)
```

```
##
## Call:
## betareg(formula = Final_0_1 ~ Spacing * GPA + Female +
##     Asian + NonWhiteOrAsian + Junior + Senior + NonNativeEnglish,
##     data = data)
##
## Quantile residuals:
##        Min         1Q     Median         3Q        Max
## -3.8174275 -0.5879502  0.0147282  0.6666923  2.8502631
##
## Coefficients (mean model with logit link):
##                          Estimate  Std. Error  z value
## (Intercept)             -1.28017159  0.51981950 -2.46272
## SpacingCounting Days     2.21472141  0.82845073  2.67333
## GPA                      0.78408523  0.15527860  5.04954
## Female1                 -0.13877974  0.12120427 -1.14501
## Asian1                   0.09928694  0.13973216  0.71055
## NonWhiteOrAsian1        -0.02625044  0.14395509 -0.18235
## Junior1                  0.31197138  0.14545673  2.14477
## Senior1                  0.38748419  0.14887812  2.60269
## NonNativeEnglish1        0.34434523  0.13228846  2.60299
```

```
## SpacingCounting Days:GPA -0.60523857  0.24087198 -2.51270
##                            Pr(>|z|)
## (Intercept)               0.0137886 *
## SpacingCounting Days      0.0075103 **
## GPA                      4.4288e-07 ***
## Female1                   0.2522062
## Asian1                    0.4773620
## NonWhiteOrAsian1          0.8553068
## Junior1                   0.0319712 *
## Senior1                   0.0092494 **
## NonNativeEnglish1         0.0092415 **
## SpacingCounting Days:GPA  0.0119812 *
##
## Phi coefficients (precision model with identity link):
##       Estimate Std. Error z value   Pr(>|z|)
## (phi) 16.25183    1.91356 8.49298 < 2.22e-16 ***
## ---
## Signif. codes:  0 '***' 0.001 '**' 0.01 '*' 0.05 '.' 0.1 ' ' 1
##
## Type of estimator: ML (maximum likelihood)
## Log-likelihood: 156.4436 on 11 Df
## Pseudo R-squared: 0.2556824
## Number of iterations: 18 (BFGS) + 2 (Fisher scoring)
```

```r
vif(Final_Model)
```

```
##          Spacing              GPA           Female
##      64.566572534      1.930910726      1.281679186
##            Asian  NonWhiteOrAsian           Junior
##      1.218646381      1.084838028      1.131176671
##           Senior NonNativeEnglish      Spacing:GPA
##      1.128655281      1.148469778     68.197721633
```

```r
Final_Model_emtrends <- emtrends(Final_Model, revpairwise ~ Spacing, var = "GPA",
                                 delta.var = 2.893 - 3.548, type = "response")
summary(Final_Model_emtrends, infer = TRUE)
```

```
## $emtrends
##  Spacing            GPA.trend     SE  df asymp.LCL
##  Counting Questions    0.1076 0.0244 Inf    0.0597
##  Counting Days         0.0194 0.0216 Inf   -0.0229
##  asymp.UCL z.ratio p.value
##     0.1555   4.403  <.0001
##     0.0618   0.899  0.3688
##
## Results are averaged over the levels of: Female, Asian, NonWhiteOrAsian, Junior, Senior, NonNativeEn
## Confidence level used: 0.95
##
## $contrasts
##  contrast                             estimate     SE  df
##  Counting Days - Counting Questions  -0.0882 0.0326 Inf
##  asymp.LCL asymp.UCL z.ratio p.value
##     -0.152   -0.0244  -2.710  0.0067
##
## Results are averaged over the levels of: Female, Asian, NonWhiteOrAsian, Junior, Senior, NonNativeEn
```

```
## Confidence level used: 0.95
```

Figure 2

Predicted final exam scores with 95% Confidence Intervals. The estimated effect of GPA on final exam scores based on the Beta regression model predictions of the within-class experiment. To improve readability, we only plot 2 <= GPA <= 4 and 0.16 <= Final_0_1 <= 1

```r
# {fig:CountingDays_GPA_Effects_on_Final_Exam_Scores}
FA2018_GPA_Terciles <- quantile(data$GPA, c(0:3/3))

FA2018_Plot <- plot_model(Final_Model, type = "eff", terms = c("GPA", "Spacing"),
                          show.data = TRUE, value.size = 4, title = "") +
  scale_x_continuous(limits = c(2, 4)) +
  scale_y_continuous(limits = c(0.16, 1), labels=scales::percent) +
  geom_vline(xintercept=FA2018_GPA_Terciles[2]) +
  geom_vline(xintercept=FA2018_GPA_Terciles[3]) +
  annotate("rect", xmin=2, xmax=FA2018_GPA_Terciles[2], ymin=0.16, ymax=0.37, alpha=0.2,
  fill="orange") +
  annotate("rect", xmin=FA2018_GPA_Terciles[2], xmax=FA2018_GPA_Terciles[3], ymin=0.16,
  ymax=0.37, alpha=0.2, fill="yellow") +
  annotate("rect", xmin=FA2018_GPA_Terciles[3], xmax=FA2018_GPA_Terciles[4], ymin=0.16,
  ymax=0.37, alpha=0.2, fill="green") +
  annotate("text", x = 2.71, y = 0.28, label = "Low GPA") +
  annotate("text", x = 2.71, y = 0.22, label = "1st Tercile") +
  annotate("text", x = 3.55, y = 0.28, label = "Mid GPA") +
  annotate("text", x = 3.55, y = 0.22, label = "2nd Tercile") +
  annotate("text", x = 3.85, y = 0.28, label = "High GPA") +
  annotate("text", x = 3.85, y = 0.22, label = "3rd Tercile") +
  scale_fill_manual(values = Counting_Days_Group_Colors) +
  scale_color_manual(values = Counting_Days_Group_Colors) +
  # guides(color = guide_legend(override.aes = list(fill = Counting_Days_Group_Colors))) +
  labs(
  x = "GPA",
  y = "Final Exam Score") +
  theme(
  legend.position = "top",
  text = element_text(size=16),
  legend.title=element_blank(),
  legend.margin=margin(c(1,5,5,5))
)
```

```
## Data points may overlap. Use the `jitter` argument to
##   add some amount of random variation to the location of
##   data points and avoid overplotting.

## Scale for y is already present.
## Adding another scale for y, which will replace the existing
## scale.
## Scale for colour is already present.
## Adding another scale for colour, which will replace the
## existing scale.
```

```r
FA2018_Plot
```

```
## Warning: Removed 3 rows containing missing values or values outside
```

```
## the scale range (`geom_point()`).

## Warning: Removed 2 rows containing missing values or values outside
## the scale range (`geom_line()`).
```

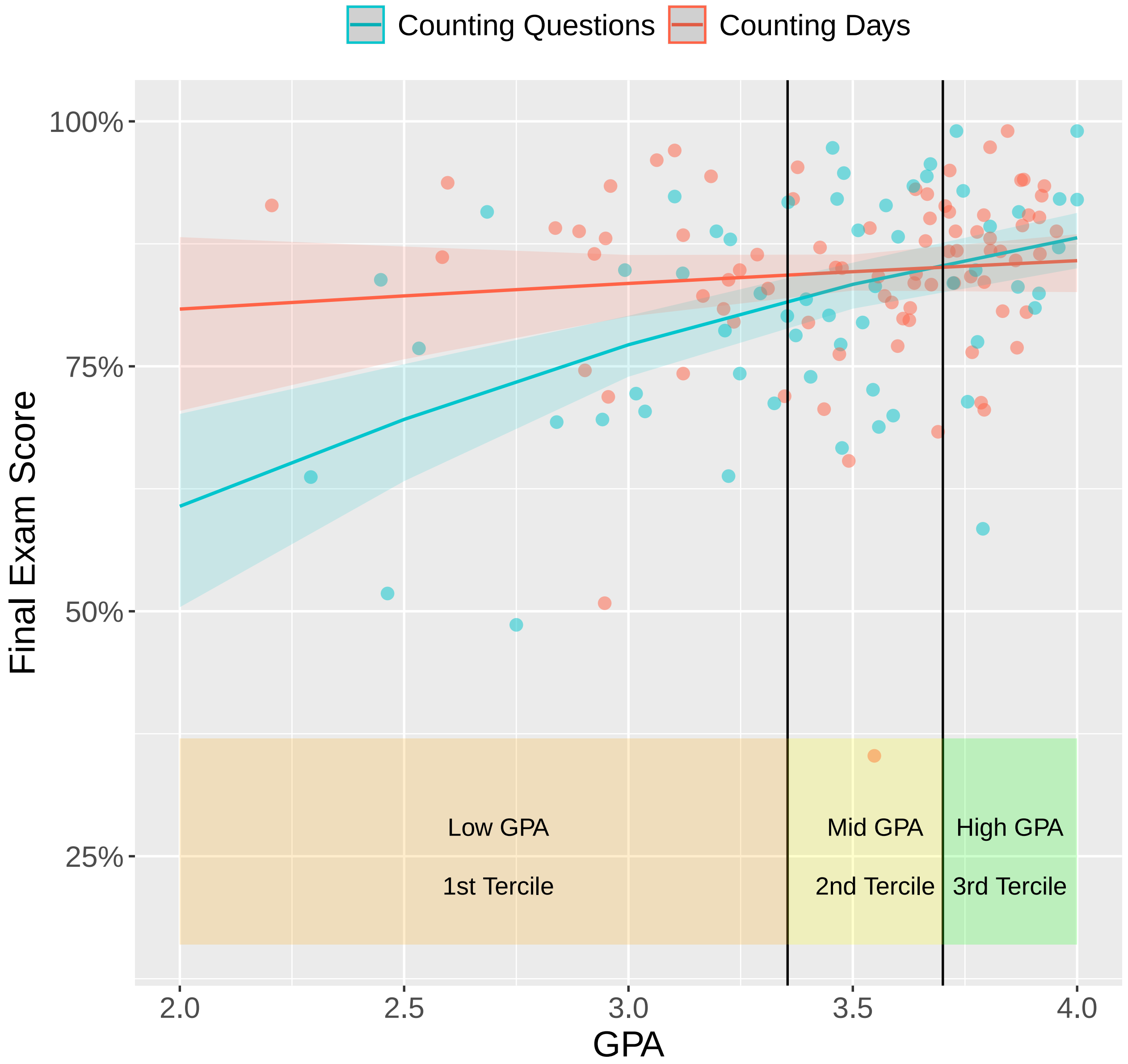

Table D2 - Column 2 - Beta regression results of the within-class experiment. The regression results without controls are included as a robustness check.

```
# {table:CountingDays_Within_Class_Experiment_Regression_Results_reduced}
Final_Model_No_Controls <- betareg(Final_0_1 ~ Spacing * GPA, data = data)
summary(Final_Model_No_Controls)
```

```
##
## Call:
## betareg(formula = Final_0_1 ~ Spacing * GPA, data = data)
##
```

```
## Quantile residuals:
##        Min         1Q     Median         3Q        Max
## -3.7945363 -0.5980623 -0.0343306  0.5902629  2.6880155
##
## Coefficients (mean model with logit link):
##                           Estimate Std. Error  z value
## (Intercept)              -0.7134386  0.5340216 -1.33597
## SpacingCounting Days      2.6247342  0.8417149  3.11832
## GPA                       0.6567482  0.1606800  4.08731
## SpacingCounting Days:GPA -0.7256544  0.2451123 -2.96050
##                           Pr(>|z|)
## (Intercept)               0.1815580
## SpacingCounting Days      0.0018189 **
## GPA                      4.3641e-05 ***
## SpacingCounting Days:GPA  0.0030714 **
##
## Phi coefficients (precision model with identity link):
##        Estimate Std. Error z value   Pr(>|z|)
## (phi) 13.572509   1.594368 8.51278 < 2.22e-16 ***
## ---
## Signif. codes:  0 '***' 0.001 '**' 0.01 '*' 0.05 '.' 0.1 ' ' 1
##
## Type of estimator: ML (maximum likelihood)
## Log-likelihood: 144.0492 on 5 Df
## Pseudo R-squared: 0.1048537
## Number of iterations: 10 (BFGS) + 2 (Fisher scoring)
```

```r
Final_Model_No_Controls_emtrends <- emtrends(Final_Model_No_Controls,
                                            revpairwise ~ Spacing, var = "GPA",
                                            delta.var = 2.893 - 3.548, type = "response")
summary(Final_Model_No_Controls_emtrends, infer = TRUE)
```

```
## $emtrends
##  Spacing            GPA.trend     SE  df asymp.LCL
##  Counting Questions   0.10900 0.0290 Inf    0.0522
##  Counting Days       -0.00902 0.0238 Inf   -0.0556
##  asymp.UCL z.ratio p.value
##     0.1658   3.764  0.0002
##     0.0376  -0.379  0.7045
##
## Confidence level used: 0.95
##
## $contrasts
##  contrast                           estimate     SE  df
##  Counting Days - Counting Questions   -0.118 0.0375 Inf
##  asymp.LCL asymp.UCL z.ratio p.value
##     -0.191   -0.0446  -3.150  0.0016
##
## Confidence level used: 0.95
```

Table D3 - Column 2 - Regression results with controls of the within-class experiment, including pass-fail students.

```
# {table:beta_with_pass_fail}
Final_Model_All_Students <- betareg(Final_0_1 ~ Spacing * GPA + Female + Asian + NonWhiteOrAsian +
                                    Junior + Senior + NonNativeEnglish, data = took_final_data)
summary(Final_Model_All_Students)
```

```
##
## Call:
## betareg(formula = Final_0_1 ~ Spacing * GPA + Female +
##     Asian + NonWhiteOrAsian + Junior + Senior + NonNativeEnglish,
##     data = took_final_data)
##
## Quantile residuals:
##        Min         1Q     Median         3Q        Max
## -5.0648585 -0.4332262  0.0473910  0.5924120  2.6930415
##
## Coefficients (mean model with logit link):
##                           Estimate  Std. Error  z value
## (Intercept)             -0.78931262  0.56610361 -1.39429
## SpacingCounting Days     1.52449163  0.87971031  1.73295
## GPA                      0.60436065  0.16536426  3.65472
## Female1                 -0.11238998  0.12616977 -0.89078
## Asian1                   0.21283618  0.15045156  1.41465
## NonWhiteOrAsian1        -0.03772234  0.15030389 -0.25097
## Junior1                  0.21290889  0.15535912  1.37043
## Senior1                  0.08513321  0.13746524  0.61931
## NonNativeEnglish1        0.37535330  0.14017562  2.67774
## SpacingCounting Days:GPA -0.39201708  0.25554434 -1.53405
##                          Pr(>|z|)
## (Intercept)             0.16323004
## SpacingCounting Days    0.08310503 .
## GPA                     0.00025746 ***
## Female1                 0.37304521
## Asian1                  0.15717139
## NonWhiteOrAsian1        0.80183433
## Junior1                 0.17055253
## Senior1                 0.53571402
## NonNativeEnglish1       0.00741216 **
## SpacingCounting Days:GPA 0.12501805
##
## Phi coefficients (precision model with identity link):
##        Estimate Std. Error z value   Pr(>|z|)
## (phi) 11.692429   1.307157 8.94493 < 2.22e-16 ***
## ---
## Signif. codes:  0 '***' 0.001 '**' 0.01 '*' 0.05 '.' 0.1 ' ' 1
##
## Type of estimator: ML (maximum likelihood)
## Log-likelihood: 144.4517 on 11 Df
## Pseudo R-squared: 0.1794082
## Number of iterations: 17 (BFGS) + 2 (Fisher scoring)
```

```r
vif(Final_Model_All_Students)
```

```
##         Spacing             GPA          Female
##    64.650310725     1.888410946     1.263030673
##           Asian NonWhiteOrAsian          Junior
##     1.193281927     1.078824107     1.143880950
##          Senior NonNativeEnglish     Spacing:GPA
##     1.127590108     1.113172390    67.478555044
```

```r
Final_Model_All_Students_emtrends <- emtrends(Final_Model_All_Students, revpairwise ~ Spacing, var = "G
                                              delta.var = 2.893 - 3.548, type = "response")
summary(Final_Model_All_Students_emtrends, infer = TRUE)
```

```
## $emtrends
##  Spacing            GPA.trend     SE  df asymp.LCL
##  Counting Questions    0.0927 0.0285 Inf    0.0369
##  Counting Days         0.0267 0.0258 Inf   -0.0239
##  asymp.UCL z.ratio p.value
##     0.1485   3.255  0.0011
##     0.0774   1.034  0.3012
##
## Results are averaged over the levels of: Female, Asian, NonWhiteOrAsian, Junior, Senior, NonNativeEn
## Confidence level used: 0.95
##
## $contrasts
##  contrast                           estimate     SE  df
##  Counting Days - Counting Questions  -0.0659 0.0387 Inf
##  asymp.LCL asymp.UCL z.ratio p.value
##     -0.142   0.00982  -1.706  0.0880
##
## Results are averaged over the levels of: Female, Asian, NonWhiteOrAsian, Junior, Senior, NonNativeEn
## Confidence level used: 0.95
```

Table 7 - Column 3 - Regression results with controls of the within-class experiment. Dependent variables are specified in column headers.

```r
# {table:CountingDays_Within_Class_Experiment_Regression_Results_with_controls}
Practice_Days_Model <- glm.nb(Practice_Days ~ Spacing * GPA + Female + Asian +
                              NonWhiteOrAsian + Junior + Senior + NonNativeEnglish,
                              data = data)
summary(Practice_Days_Model)
```

```
##
## Call:
## glm.nb(formula = Practice_Days ~ Spacing * GPA + Female + Asian +
##     NonWhiteOrAsian + Junior + Senior + NonNativeEnglish, data = data,
##     init.theta = 266.874665, link = log)
##
## Coefficients:
##                             Estimate  Std. Error  z value
## (Intercept)               2.05607518  0.24507226  8.38967
## SpacingCounting Days      1.63829475  0.30528019  5.36653
## GPA                       0.22262960  0.07160619  3.10908
## Female1                   0.13323689  0.03994598  3.33543
```

```
## Asian1                    -0.09148266  0.04347453 -2.10428
## NonWhiteOrAsian1          -0.06645826  0.04729146 -1.40529
## Junior1                   -0.05886352  0.04493183 -1.31006
## Senior1                   -0.03593569  0.04609380 -0.77962
## NonNativeEnglish1          0.01086901  0.04124817  0.26350
## SpacingCounting Days:GPA -0.24675300  0.08806500 -2.80194
##                            Pr(>|z|)
## (Intercept)              < 2.22e-16 ***
## SpacingCounting Days     8.0267e-08 ***
## GPA                      0.00187669 **
## Female1                  0.00085169 ***
## Asian1                   0.03535388 *
## NonWhiteOrAsian1         0.15993484
## Junior1                  0.19017460
## Senior1                  0.43561406
## NonNativeEnglish1        0.79216302
## SpacingCounting Days:GPA 0.00507961 **
## ---
## Signif. codes:
## 0 '***' 0.001 '**' 0.01 '*' 0.05 '.' 0.1 ' ' 1
##
## (Dispersion parameter for Negative Binomial(266.8747) family taken to be 1)
##
##     Null deviance: 742.26784  on 142  degrees of freedom
## Residual deviance: 195.00702  on 133  degrees of freedom
## AIC: 962.69579
##
## Number of Fisher Scoring iterations: 1
##
##
##                Theta:  267
##            Std. Err.:  412
##
##  2 x log-likelihood:  -940.696
```

```
vif(Practice_Days_Model)
```

```
## there are higher-order terms (interactions) in this model
## consider setting type = 'predictor'; see ?vif

##          Spacing              GPA           Female
##     67.261214538      3.233698015      1.334252203
##            Asian  NonWhiteOrAsian           Junior
##      1.248871205      1.084608802      1.133419390
##           Senior NonNativeEnglish      Spacing:GPA
##      1.158432526      1.120049785     71.593296004
```

```
Practice_Days_Model_emtrends <- emtrends(Practice_Days_Model, revpairwise ~ Spacing,
var = "GPA", delta.var = 2.920 - 3.879,
type = "response")
summary(Practice_Days_Model_emtrends, infer = TRUE)
```

```
## $emtrends
##  Spacing            GPA.trend     SE  df asymp.LCL
##  Counting Questions    0.2226 0.0716 Inf    0.0823
##  Counting Days        -0.0241 0.0544 Inf   -0.1307
```

```
##  asymp.UCL z.ratio p.value
##     0.3630   3.109  0.0019
##     0.0825  -0.444  0.6573
##
## Results are averaged over the levels of: Female, Asian, NonWhiteOrAsian, Junior, Senior, NonNativeEng
## Confidence level used: 0.95
##
## $contrasts
##  contrast                           estimate     SE  df
##  Counting Days - Counting Questions   -0.247 0.0881 Inf
##  asymp.LCL asymp.UCL z.ratio p.value
##     -0.419   -0.0741  -2.802  0.0051
##
## Results are averaged over the levels of: Female, Asian, NonWhiteOrAsian, Junior, Senior, NonNativeEng
## Confidence level used: 0.95
```

Figure 5

Predictive margins of counting days incentive with 95% confidence intervals. Estimated effects of GPA on number of days practiced for each of the experimental conditions in the within-class experiment. To improve readability, we only plot 2 <= GPA <= 4.

```
# {fig:CountingDays_GPA_Effects_on_Days_Practiced}
plot_model(Practice_Days_Model, type = "eff", terms = c("GPA", "Spacing"), title = "",
          show.data = TRUE) +
  scale_x_continuous(limits = c(2, 4)) +
  geom_vline(xintercept=FA2018_GPA_Terciles[2]) +
  geom_vline(xintercept=FA2018_GPA_Terciles[3]) +
  annotate("rect", xmin=2, xmax=FA2018_GPA_Terciles[2], ymin=0, ymax=10, alpha=0.2,
  fill="orange") +
  annotate("rect", xmin=FA2018_GPA_Terciles[2], xmax=FA2018_GPA_Terciles[3], ymin=0,
  ymax=10, alpha=0.2, fill="yellow") +
  annotate("rect", xmin=FA2018_GPA_Terciles[3], xmax=FA2018_GPA_Terciles[4], ymin=0,
  ymax=10, alpha=0.2, fill="green") +
  annotate("text", x = 2.71, y = 7, label = "Low GPA") +
  annotate("text", x = 2.71, y = 3.1, label = "1st Tercile") +
  annotate("text", x = 3.535, y = 7, label = "Mid GPA") +
  annotate("text", x = 3.535, y = 3.1, label = "2nd Tercile") +
  annotate("text", x = 3.85, y = 7, label = "High GPA") +
  annotate("text", x = 3.85, y = 3.1, label = "3rd Tercile") +
  scale_fill_manual(values = Counting_Days_Group_Colors) +
  scale_color_manual(values = Counting_Days_Group_Colors) +
  # guides(color = guide_legend(override.aes = list(fill = Counting_Days_Group_Colors))) +
  labs(
    x = "GPA",
    y = "Number of Days Practiced",
    color = "Counting_Days",
    fill = "Counting_Days") +
  theme(
    legend.position = "top",
    text = element_text(size=16),
    legend.title=element_blank(),
    legend.margin=margin(c(1,5,5,5))
)
```

```
## Data points may overlap. Use the `jitter` argument to
##   add some amount of random variation to the location of
##   data points and avoid overplotting.

## Scale for colour is already present.
## Adding another scale for colour, which will replace the
## existing scale.

## Warning: Removed 3 rows containing missing values or values outside
## the scale range (`geom_point()`).

## Warning: Removed 2 rows containing missing values or values outside
## the scale range (`geom_line()`).
```

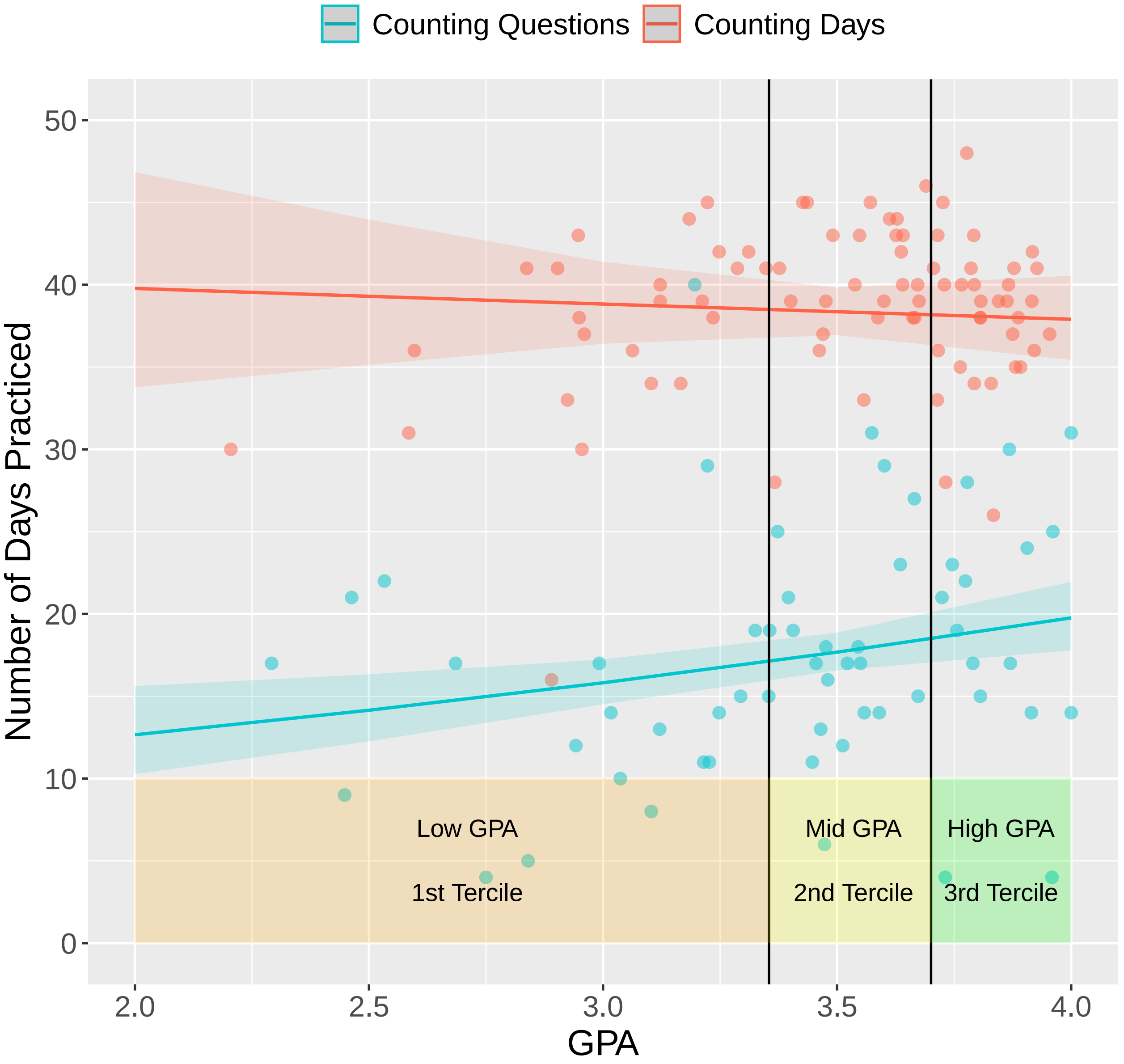

Table D2 - Column 3 - Beta regression results of the within-class experiment. The regression results without controls are included as a robustness check.

```
# {table:CountingDays_Within_Class_Experiment_Regression_Results_reduced}
Practice_Days_Model_No_Controls <- glm.nb(Practice_Days ~ Spacing * GPA, data = data)
summary(Practice_Days_Model_No_Controls)
```

```
##
## Call:
## glm.nb(formula = Practice_Days ~ Spacing * GPA, data = data,
##     init.theta = 95.89454937, link = log)
##
## Coefficients:
##                             Estimate  Std. Error  z value
## (Intercept)               2.01095877  0.25463266  7.89749
## SpacingCounting Days      1.35983223  0.31554752  4.30944
## GPA                       0.24798708  0.07411855  3.34582
## SpacingCounting Days:GPA -0.16771379  0.09114376 -1.84010
##                             Pr(>|z|)
## (Intercept)              2.8458e-15 ***
## SpacingCounting Days     1.6367e-05 ***
## GPA                      0.00082041 ***
## SpacingCounting Days:GPA 0.06575329 .
## ---
## Signif. codes:
## 0 '***' 0.001 '**' 0.01 '*' 0.05 '.' 0.1 ' ' 1
##
## (Dispersion parameter for Negative Binomial(95.8945) family taken to be 1)
##
##     Null deviance: 643.45634  on 142  degrees of freedom
## Residual deviance: 191.60169  on 139  degrees of freedom
## AIC: 970.20116
##
## Number of Fisher Scoring iterations: 1
##
##
##               Theta:  95.9
##           Std. Err.:  64.3
##
##  2 x log-likelihood:  -960.201
```

```
Practice_Days_Model_No_Controls_emtrends <- emtrends(Practice_Days_Model_No_Controls,
                                                     revpairwise ~ Spacing, var = "GPA",
                                                     delta.var = 2.920 - 3.879,
                                                     type = "response")
summary(Practice_Days_Model_No_Controls_emtrends, infer = TRUE)
```

```
## $emtrends
##  Spacing            GPA.trend     SE  df asymp.LCL
##  Counting Questions    0.2480 0.0741 Inf    0.1027
##  Counting Days         0.0803 0.0530 Inf   -0.0237
##  asymp.UCL z.ratio p.value
##      0.393   3.346  0.0008
##      0.184   1.513  0.1302
##
```

```
## Confidence level used: 0.95
##
## $contrasts
##  contrast                           estimate     SE  df
##  Counting Days - Counting Questions   -0.168 0.0911 Inf
##  asymp.LCL asymp.UCL z.ratio p.value
##     -0.346    0.0109  -1.840  0.0658
##
## Confidence level used: 0.95
```

Table D3 - Column 2 - Regression results with controls of the within-class experiment, including pass-fail students.

```r
# {table:beta_with_pass_fail}
Practice_Days_Model_All_Students <- glm.nb(Practice_Days ~ Spacing * GPA + Female + Asian +
                              NonWhiteOrAsian + Junior + Senior + NonNativeEnglish,
                              data = took_final_data)
summary(Practice_Days_Model_All_Students)
```

```
##
## Call:
## glm.nb(formula = Practice_Days ~ Spacing * GPA + Female + Asian +
##     NonWhiteOrAsian + Junior + Senior + NonNativeEnglish, data = took_final_data,
##     init.theta = 117.7378694, link = log)
##
## Coefficients:
##                              Estimate   Std. Error  z value
## (Intercept)                2.208321511  0.243481126  9.06979
## SpacingCounting Days       1.486702379  0.307384471  4.83662
## GPA                        0.169913310  0.070131632  2.42278
## Female1                    0.150719216  0.039839044  3.78320
## Asian1                    -0.069340467  0.044314045 -1.56475
## NonWhiteOrAsian1          -0.076595514  0.048105253 -1.59225
## Junior1                   -0.058802800  0.046154231 -1.27405
## Senior1                   -0.093754792  0.043198355 -2.17033
## NonNativeEnglish1          0.005016504  0.042082467  0.11921
## SpacingCounting Days:GPA -0.195442456  0.088429819 -2.21014
##                            Pr(>|z|)
## (Intercept)               < 2.22e-16 ***
## SpacingCounting Days      1.3206e-06 ***
## GPA                       0.01540238 *
## Female1                   0.00015482 ***
## Asian1                    0.11764120
## NonWhiteOrAsian1          0.11132890
## Junior1                   0.20264567
## Senior1                   0.02998167 *
## NonNativeEnglish1         0.90511174
## SpacingCounting Days:GPA 0.02709531 *
## ---
## Signif. codes:
## 0 '***' 0.001 '**' 0.01 '*' 0.05 '.' 0.1 ' ' 1
##
## (Dispersion parameter for Negative Binomial(117.7379) family taken to be 1)
##
```

```
##     Null deviance: 794.96369  on 156  degrees of freedom
## Residual deviance: 221.27434  on 147  degrees of freedom
## AIC: 1073.9677
##
## Number of Fisher Scoring iterations: 1
##
##
##               Theta:  117.7
##           Std. Err.:  91.7
##
##  2 x log-likelihood:  -1051.968
```

```
vif(Practice_Days_Model_All_Students)
```

```
## there are higher-order terms (interactions) in this model
## consider setting type = 'predictor'; see ?vif
```

```
##          Spacing              GPA           Female
##     67.875383015      3.029623899      1.282116337
##            Asian  NonWhiteOrAsian           Junior
##      1.186512557      1.076561429      1.138025478
##           Senior NonNativeEnglish      Spacing:GPA
##      1.128150373      1.088238607     71.163989957
```

```
Practice_Days_Model_All_Students_emtrends <- emtrends(Practice_Days_Model_All_Students, revpairwise ~ Sp
var = "GPA", delta.var = 2.920 - 3.879,
type = "response")
summary(Practice_Days_Model_All_Students_emtrends, infer = TRUE)
```

```
## $emtrends
##  Spacing            GPA.trend     SE  df asymp.LCL
##  Counting Questions    0.1699 0.0701 Inf    0.0325
##  Counting Days        -0.0255 0.0551 Inf   -0.1335
##  asymp.UCL z.ratio p.value
##     0.3074   2.423  0.0154
##     0.0825  -0.463  0.6432
##
## Results are averaged over the levels of: Female, Asian, NonWhiteOrAsian, Junior, Senior, NonNativeEng
## Confidence level used: 0.95
##
## $contrasts
##  contrast                           estimate     SE  df
##  Counting Days - Counting Questions   -0.195 0.0884 Inf
##  asymp.LCL asymp.UCL z.ratio p.value
##     -0.369   -0.0221  -2.210  0.0271
##
## Results are averaged over the levels of: Female, Asian, NonWhiteOrAsian, Junior, Senior, NonNativeEng
## Confidence level used: 0.95
```

Table 7 - Column 4 - Regression results with controls of the within-class experiment. Dependent variables are specified in column headers.

```
# {table:CountingDays_Within_Class_Experiment_Regression_Results_with_controls}
Practice_Count_Model <- glm.nb(Practice_Count ~ Spacing * GPA + Female + Asian +
                          NonWhiteOrAsian + Junior + Senior + NonNativeEnglish,
```

```r
                          data = data)
summary(Practice_Count_Model)
```

```
##
## Call:
## glm.nb(formula = Practice_Count ~ Spacing * GPA + Female + Asian +
##     NonWhiteOrAsian + Junior + Senior + NonNativeEnglish, data = data,
##     init.theta = 63.43101783, link = log)
##
## Coefficients:
##                              Estimate   Std. Error  z value
## (Intercept)                5.674737760  0.125574813 45.19010
## SpacingCounting Days       0.215552128  0.185863895  1.15973
## GPA                        0.092637796  0.037049317  2.50039
## Female1                    0.018084921  0.026814632  0.67444
## Asian1                    -0.040827798  0.030100096 -1.35640
## NonWhiteOrAsian1          -0.034635391  0.032238511 -1.07435
## Junior1                   -0.061717263  0.030983670 -1.99193
## Senior1                    0.004437875  0.031373063  0.14145
## NonNativeEnglish1         -0.003629757  0.027849652 -0.13033
## SpacingCounting Days:GPA -0.048804299  0.053758629 -0.90784
##                          Pr(>|z|)
## (Intercept)               < 2e-16 ***
## SpacingCounting Days     0.246158
## GPA                      0.012406 *
## Female1                  0.500030
## Asian1                   0.174972
## NonWhiteOrAsian1         0.282667
## Junior1                  0.046379 *
## Senior1                  0.887511
## NonNativeEnglish1        0.896302
## SpacingCounting Days:GPA 0.363962
## ---
## Signif. codes:
## 0 '***' 0.001 '**' 0.01 '*' 0.05 '.' 0.1 ' ' 1
##
## (Dispersion parameter for Negative Binomial(63.431) family taken to be 1)
##
##     Null deviance: 171.11547  on 142  degrees of freedom
## Residual deviance: 145.97428  on 133  degrees of freedom
## AIC: 1573.2691
##
## Number of Fisher Scoring iterations: 1
##
##
##               Theta:  63.43
##           Std. Err.:  8.82
##
##  2 x log-likelihood:  -1551.269
```

```r
vif(Practice_Count_Model)
```

```
## there are higher-order terms (interactions) in this model
## consider setting type = 'predictor'; see ?vif
```

```
##          Spacing              GPA           Female
##      65.882893205      2.082790428      1.312822819
##            Asian  NonWhiteOrAsian           Junior
##      1.257664382      1.097677431      1.145381025
##           Senior NonNativeEnglish      Spacing:GPA
##      1.148599206      1.142591879     68.996434246
```

```
Practice_Count_Model_emtrends <- emtrends(Practice_Count_Model, revpairwise ~ Spacing,
                                          var = "GPA", delta.var = 2.920 - 3.879,
                                          type = "response")
summary(Practice_Count_Model_emtrends, infer = TRUE)
```

```
## $emtrends
##  Spacing            GPA.trend     SE  df asymp.LCL
##  Counting Questions    0.0926 0.0370 Inf    0.0200
##  Counting Days         0.0438 0.0406 Inf   -0.0357
##  asymp.UCL z.ratio p.value
##      0.165   2.500  0.0124
##      0.123   1.080  0.2803
##
## Results are averaged over the levels of: Female, Asian, NonWhiteOrAsian, Junior, Senior, NonNativeEn
## Confidence level used: 0.95
##
## $contrasts
##  contrast                           estimate     SE  df
##  Counting Days - Counting Questions  -0.0488 0.0538 Inf
##  asymp.LCL asymp.UCL z.ratio p.value
##     -0.154    0.0566  -0.908  0.3640
##
## Results are averaged over the levels of: Female, Asian, NonWhiteOrAsian, Junior, Senior, NonNativeEn
## Confidence level used: 0.95
```

Table D2 - Column 4 - Beta regression results of the within-class experiment. The regression results without controls are included as a robustness check.

```
# {table:CountingDays_Within_Class_Experiment_Regression_Results_reduced}
Practice_Count_Model_No_Controls <- glm.nb(Practice_Count ~ Spacing * GPA, data = data)
summary(Practice_Count_Model_No_Controls)
```

```
##
## Call:
## glm.nb(formula = Practice_Count ~ Spacing * GPA, data = data,
##     init.theta = 59.10958215, link = log)
##
## Coefficients:
##                             Estimate  Std. Error  z value
## (Intercept)               5.64517530  0.12704007 44.43618
## SpacingCounting Days      0.13873991  0.18444550  0.75220
## GPA                       0.09714005  0.03741099  2.59656
## SpacingCounting Days:GPA -0.02708843  0.05342212 -0.50706
##                            Pr(>|z|)
## (Intercept)              < 2.22e-16 ***
## SpacingCounting Days      0.4519308
## GPA                       0.0094161 **
```

```
## SpacingCounting Days:GPA  0.6121100
## ---
## Signif. codes:
## 0 '***' 0.001 '**' 0.01 '*' 0.05 '.' 0.1 ' ' 1
##
## (Dispersion parameter for Negative Binomial(59.1096) family taken to be 1)
##
##     Null deviance: 161.10630  on 142  degrees of freedom
## Residual deviance: 145.65118  on 139  degrees of freedom
## AIC: 1569.7155
##
## Number of Fisher Scoring iterations: 1
##
##
##               Theta:  59.11
##           Std. Err.:  8.12
##
##  2 x log-likelihood:  -1559.716
```

```
Practice_Count_Model_No_Controls_emtrends <- emtrends(Practice_Count_Model_No_Controls,
                                                      revpairwise ~ Spacing, var = "GPA",
                                                      delta.var = 2.920 - 3.879,
                                                      type = "response")
summary(Practice_Count_Model_No_Controls_emtrends, infer = TRUE)
```

```
## $emtrends
##  Spacing            GPA.trend     SE  df asymp.LCL
##  Counting Questions    0.0971 0.0374 Inf   0.02382
##  Counting Days         0.0701 0.0381 Inf  -0.00469
##  asymp.UCL z.ratio p.value
##      0.170   2.597  0.0094
##      0.145   1.837  0.0662
##
## Confidence level used: 0.95
##
## $contrasts
##  contrast                            estimate     SE  df
##  Counting Days - Counting Questions   -0.0271 0.0534 Inf
##  asymp.LCL asymp.UCL z.ratio p.value
##     -0.132    0.0776  -0.507  0.6121
##
## Confidence level used: 0.95
```

Table D3 - Column 4 - Regression results with controls of the within-class experiment, including pass-fail students.

```
# {table:beta_with_pass_fail}
Practice_Count_Model_All_Students <- glm.nb(Practice_Count ~ Spacing * GPA + Female + Asian +
                          NonWhiteOrAsian + Junior + Senior + NonNativeEnglish, data = took_final_data)
summary(Practice_Count_Model_All_Students)
```

```
##
## Call:
## glm.nb(formula = Practice_Count ~ Spacing * GPA + Female + Asian +
```

```
##     NonWhiteOrAsian + Junior + Senior + NonNativeEnglish, data = took_final_data,
##     init.theta = 20.97594366, link = log)
##
## Coefficients:
##                            Estimate  Std. Error  z value
## (Intercept)               5.74532628  0.19888120 28.88823
## SpacingCounting Days      0.18573766  0.29392662  0.63192
## GPA                       0.06164845  0.05761566  1.06999
## Female1                   0.04623598  0.04148198  1.11460
## Asian1                   -0.03631541  0.04777237 -0.76018
## NonWhiteOrAsian1         -0.06490928  0.05014255 -1.29449
## Junior1                  -0.04847581  0.04952061 -0.97890
## Senior1                  -0.04985905  0.04506795 -1.10631
## NonNativeEnglish1         0.02266931  0.04392194  0.51613
## SpacingCounting Days:GPA -0.03151075  0.08493755 -0.37099
##                          Pr(>|z|)
## (Intercept)               < 2e-16 ***
## SpacingCounting Days      0.52744
## GPA                       0.28462
## Female1                   0.26502
## Asian1                    0.44715
## NonWhiteOrAsian1          0.19549
## Junior1                   0.32763
## Senior1                   0.26859
## NonNativeEnglish1         0.60577
## SpacingCounting Days:GPA  0.71065
## ---
## Signif. codes:
## 0 '***' 0.001 '**' 0.01 '*' 0.05 '.' 0.1 ' ' 1
##
## (Dispersion parameter for Negative Binomial(20.9759) family taken to be 1)
##
##     Null deviance: 197.01669  on 156  degrees of freedom
## Residual deviance: 183.74302  on 147  degrees of freedom
## AIC: 1898.653
##
## Number of Fisher Scoring iterations: 1
##
##
##               Theta:  20.98
##           Std. Err.:  2.75
##
##  2 x log-likelihood:  -1876.653
```

```
Practice_Count_Model_All_Students_emtrends <- emtrends(Practice_Count_Model_All_Students,
                                                      revpairwise ~ Spacing, var = "GPA",
                                                      delta.var = 2.920 - 3.879,
                                                      type = "response")
summary(Practice_Count_Model_All_Students_emtrends, infer = TRUE)
```

```
## $emtrends
##  Spacing             GPA.trend     SE  df asymp.LCL
##  Counting Questions     0.0616 0.0576 Inf   -0.0513
##  Counting Days          0.0301 0.0634 Inf   -0.0941
##  asymp.UCL z.ratio p.value
```

```
##      0.175   1.070  0.2846
##      0.154   0.475  0.6345
##
## Results are averaged over the levels of: Female, Asian, NonWhiteOrAsian, Junior, Senior, NonNativeEng
## Confidence level used: 0.95
##
## $contrasts
##  contrast                           estimate     SE  df
##  Counting Days - Counting Questions  -0.0315 0.0849 Inf
##  asymp.LCL asymp.UCL z.ratio p.value
##     -0.198     0.135  -0.371  0.7106
##
## Results are averaged over the levels of: Female, Asian, NonWhiteOrAsian, Junior, Senior, NonNativeEng
## Confidence level used: 0.95
```

Tables 7, D2, D3 - Defining functions to generate regression results of the within-class experiment.

```
# Function to extract model results and format them
format_model_results <- function(model, model_type) {
  if (model_type == "betareg") {
    tidy_model <- tidy(model)
    # Separate mean and precision coefficients
    mean_coefs <- tidy_model %>% dplyr::filter(component == "mean")
    mean_coefs$param_name <- NA  # Add param_name column
    precision_coefs <- tidy_model %>% dplyr::filter(component == "precision")
    # Rename the precision coefficient to 'Parameter' and set param_name
    precision_coefs$term <- "Parameter"
    precision_coefs$param_name <- "Scale"
    # Combine mean and precision coefficients
    tidy_model <- dplyr::bind_rows(mean_coefs, precision_coefs)
  } else if (model_type == "glm.nb") {
    tidy_model <- tidy(model)
    tidy_model$param_name <- NA  # Add param_name column
    # Add Alpha parameter
    alpha <- 1 / model$theta
    se_alpha <- model$SE.theta / model$theta^2
    p_alpha <- 2 * pnorm(-abs(alpha / se_alpha))
    alpha_row <- data.frame(
      term = "Parameter",
      estimate = alpha,
      std.error = se_alpha,
      statistic = NA,
      p.value = p_alpha,
      param_name = "Alpha",
      stringsAsFactors = FALSE
    )
    tidy_model <- dplyr::bind_rows(tidy_model, alpha_row)
  }
  return(tidy_model)
}

# Function to prepare results for LaTeX table
```

```r
prepare_table_data <- function(model_results, terms_map, terms_order) {
  # Merge with terms_map to get labels
  model_results <- model_results %>%
    dplyr::right_join(terms_map, by = "term") %>%
    dplyr::arrange(match(term, terms_order))

  # Calculate significance stars
  model_results <- model_results %>%
    dplyr::mutate(
      stars = dplyr::case_when(
        p.value < 0.001 ~ "***",
        p.value < 0.01 ~ "**",
        p.value < 0.05 ~ "*",
        TRUE ~ ""
      ),
      # Format estimate with stars
      estimate_fmt = sprintf("%.3f%s", estimate, stars),
      # Format standard error
      std.error_fmt = ifelse(!is.na(std.error), sprintf("(%.3f)", std.error), ""),
      # For 'Parameter', include param_name in the cell
      latex_cell = ifelse(term == "Parameter",
                          sprintf("\\begin{tabular}[c]{@{}l@{}}\\textbf{%s:}\\\\ %s\\\\ %s\\end{tabular}
                                  param_name, estimate_fmt, std.error_fmt),
                          sprintf("\\begin{tabular}[c]{@{}l@{}}%s\\\\ %s\\end{tabular}", estimate_fmt, s
      )
    )

  # Select only necessary columns
  model_results <- model_results %>%
    dplyr::select(label, latex_cell)
  return(model_results)
}

# Extract Log Likelihood, Number of Observations, and Chi-squared
get_model_stats <- function(model, model_type) {
  if (model_type == "betareg") {
    logLik_value <- as.numeric(logLik(model))
    nobs_value <- nobs(model)
    chi_sq_value <- summary(model)$pseudo.r.squared * 100  # Convert to percentage
    chi_sq_stars <- "***"  # Adjust as needed
    chi_sq_formatted <- sprintf("%.3f", chi_sq_value)
  } else if (model_type == "glm.nb") {
    logLik_value <- as.numeric(logLik(model))
    nobs_value <- nobs(model)
    chi_sq_value <- summary(model)$deviance
    chi_sq_stars <- ""  # Deviance is not a p-value; adjust as needed
    chi_sq_formatted <- sprintf("%.3f", chi_sq_value)
  }
  return(list(
    LogLik = sprintf("%.3f", logLik_value),
    Nobs = nobs_value,
    ChiSq = sprintf("\\begin{tabular}[c]{@{}l@{}}%s\\\\ %s\\end{tabular}", chi_sq_formatted, chi_sq_sta
  ))
```

```
}

# Generate the LaTeX code for the table
generate_latex_table <- function(table_data, header, footer) {
  # Body of the table
  body <- ""
  for (i in 1:nrow(table_data)) {
    row_label <- table_data$label[i]
    final_cell <- table_data$Final[i]
    days_cell <- table_data$Days[i]
    questions_cell <- table_data$Questions[i]
    # Add LaTeX line
    body <- paste0(body, row_label, " & ", final_cell, " & ", days_cell, " & ", questions_cell, " \\\\\[illegible]
    # Add \hline after specific rows
    if (i %in% c(1:10, nrow(table_data))) {  # Adjust as needed
      body <- paste0(body, "\\hline\n")
    }
  }
  # Combine header, body, and footer
  latex_table <- paste0(header, body, footer)
  return(latex_table)
}

# Generate the LaTeX code for the table
generate_latex_table_of_three_models <- function(
    model1,
    model1Type,
    model2,
    model2Type,
    model3,
    model3Type,
    header,
    footer,
    terms_order,
    terms_labels) {

  # Extract and format results for each model
  final_results <- format_model_results(model1, model1Type)
  days_results <- format_model_results(model2, model2Type)
  questions_results <- format_model_results(model3, model3Type)

  # Create the terms_map data frame
  terms_map <- data.frame(
    term = terms_order,
    label = terms_labels,
    stringsAsFactors = FALSE
  )

  # Prepare data for each model
  final_table <- prepare_table_data(final_results, terms_map, terms_order)
  days_table <- prepare_table_data(days_results, terms_map, terms_order)
  questions_table <- prepare_table_data(questions_results, terms_map, terms_order)
```

```
  # Combine all models into one table
  combined_table <- data.frame(
    label = final_table$label,
    Final = final_table$latex_cell,
    Days = days_table$latex_cell,
    Questions = questions_table$latex_cell,
    stringsAsFactors = FALSE
  )

  # Handle missing values (if any)
  combined_table[is.na(combined_table)] <- ""

  # Add goodness-of-fit statistics

  final_stats <- get_model_stats(model1, model1Type)
  days_stats <- get_model_stats(model2, model2Type)
  questions_stats <- get_model_stats(model3, model3Type)

  # Add stats to the table
  stats_labels <- c(
    "\\textbf{Log Likelihood}",
    "\\textbf{\\# of Obs.}",
    "\\textbf{$\\chi^2$}"
  )

  stats_table <- data.frame(
    label = stats_labels,
    Final = c(final_stats$LogLik, final_stats$Nobs, final_stats$ChiSq),
    Days = c(days_stats$LogLik, days_stats$Nobs, days_stats$ChiSq),
    Questions = c(questions_stats$LogLik, questions_stats$Nobs, questions_stats$ChiSq),
    stringsAsFactors = FALSE
  )

  # Combine stats with coefficients
  full_table <- dplyr::bind_rows(combined_table, stats_table)

  # Generate the LaTeX table code
  cat(generate_latex_table(full_table, header, footer))
}
```

Table 7 - Regression results with controls of the within-class experiment.

```
# {table:CountingDays_Within_Class_Experiment_Regression_Results_with_controls}
# Update your terms_order and terms_labels to match your models
terms_order <- c(
  "SpacingCounting Days",
  "GPA",
  "SpacingCounting Days:GPA",
  "Female1",
  "Asian1",
  "NonWhiteOrAsian1",
  "Junior1",
  "Senior1",
```

```r
  "NonNativeEnglish1",
  "(Intercept)",
  "Parameter"
)

# Corresponding labels
terms_labels <- c(
  "\\begin{tabular}[c]{@{}l@{}}\\textbf{Counting Days}\\\\ \\textbf{(vs. Questions)}\\end{tabular}",
  "\\textbf{GPA}",
  "\\begin{tabular}[c]{@{}l@{}}\\textbf{Counting Days}\\\\ \\textbf{$\\times$ GPA}\\end{tabular}",
  "\\begin{tabular}[c]{@{}l@{}}\\textbf{Female}\\\\ \\textbf{(vs. Male)}\\end{tabular}",
  "\\begin{tabular}[c]{@{}l@{}}\\textbf{Asian}\\\\ \\textbf{(vs. White)}\\end{tabular}",
  "\\begin{tabular}[c]{@{}l@{}}\\textbf{NonWhiteOrAsian}\\\\ \\textbf{(vs. White)}\\end{tabular}",
  "\\begin{tabular}[c]{@{}l@{}}\\textbf{Junior}\\\\ \\textbf{(vs. Sophomore)}\\end{tabular}",
  "\\begin{tabular}[c]{@{}l@{}}\\textbf{Senior}\\\\ \\textbf{(vs. Sophomore)}\\end{tabular}",
  "\\begin{tabular}[c]{@{}l@{}}\\textbf{NonNativeEnglish}\\\\ \\textbf{(vs. NativeEnglish)}\\end{tabula
  "\\textbf{[Intercept]}",
  "\\textbf{[Parameter]}"
)

# Header of the table
header <- "\\begin{table*}[!htp]
\\centering
\\ra{1.21}
\\begin{tabular}{@{}p{3.1cm}lll@{}}
\\toprule
\\multicolumn{1}{c}{\\textbf{}} & \\textbf{Final} & \\textbf{Days} & \\textbf{Questions} \\\\
\\multicolumn{1}{c}{\\textbf{}} & \\textbf{Exam Score} & \\textbf{Practiced} & \\textbf{Practiced} \\\\
\\multicolumn{1}{l}{\\textbf{}} & \\textbf{(Beta)} & \\textbf{(Neg. Bin.)} & \\textbf{(Neg. Bin.)} \\\\
\\midrule
"

# Footer of the table
footer <- "\\bottomrule
\\end{tabular}
\\caption{Regression results with controls of the within-class experiment. Dependent variables are spec
\\label{table:CountingDays_Within_Class_Experiment_Regression_Results_with_controls}
\\end{table*}
"

# Generate the LaTeX code for the table
generate_latex_table_of_three_models(
  Final_Model,
  "betareg",
  Practice_Days_Model,
  "glm.nb",
  Practice_Count_Model,
  "glm.nb",
  header,
  footer,
  terms_order,
  terms_labels
)
```

```
## \begin{table*}[!htp]
## \centering
## \ra{1.21}
## \begin{tabular}{@{}p{3.1cm}lll@{}}
## \toprule
## \multicolumn{1}{c}{\textbf{}} & \textbf{Final} & \textbf{Days} & \textbf{Questions} \\
## \multicolumn{1}{c}{\textbf{}} & \textbf{Exam Score} & \textbf{Practiced} & \textbf{Practiced} \\
## \multicolumn{1}{l}{\textbf{}} & \textbf{(Beta)} & \textbf{(Neg. Bin.)} & \textbf{(Neg. Bin.)} \\
## \midrule
## \begin{tabular}[c]{@{}l@{}}\textbf{Counting Days}\\ \textbf{(vs. Questions)}\end{tabular} & \begin{ta
## \hline
## \textbf{GPA} & \begin{tabular}[c]{@{}l@{}}0.784***\\ (0.155)\end{tabular} & \begin{tabular}[c]{@{}l@
## \hline
## \begin{tabular}[c]{@{}l@{}}\textbf{Counting Days}\\ \textbf{$\times$ GPA}\end{tabular} & \begin{tabul
## \hline
## \begin{tabular}[c]{@{}l@{}}\textbf{Female}\\ \textbf{(vs. Male)}\end{tabular} & \begin{tabular}[c]{@
## \hline
## \begin{tabular}[c]{@{}l@{}}\textbf{Asian}\\ \textbf{(vs. White)}\end{tabular} & \begin{tabular}[c]{@
## \hline
## \begin{tabular}[c]{@{}l@{}}\textbf{NonWhiteOrAsian}\\ \textbf{(vs. White)}\end{tabular} & \begin{tabu
## \hline
## \begin{tabular}[c]{@{}l@{}}\textbf{Junior}\\ \textbf{(vs. Sophomore)}\end{tabular} & \begin{tabular}
## \hline
## \begin{tabular}[c]{@{}l@{}}\textbf{Senior}\\ \textbf{(vs. Sophomore)}\end{tabular} & \begin{tabular}
## \hline
## \begin{tabular}[c]{@{}l@{}}\textbf{NonNativeEnglish}\\ \textbf{(vs. NativeEnglish)}\end{tabular} & \
## \hline
## \textbf{[Intercept]} & \begin{tabular}[c]{@{}l@{}}-1.280*\\ (0.520)\end{tabular} & \begin{tabular}[c
## \hline
## \textbf{[Parameter]} & \begin{tabular}[c]{@{}l@{}}\textbf{Scale:}\\ 16.252***\\ (1.914)\end{tabular}
## \textbf{Log Likelihood} & 156.444 & -470.348 & -775.635 \\
## \textbf{\# of Obs.} & 143 & 143 & 143 \\
## \textbf{$\chi^2$} & \begin{tabular}[c]{@{}l@{}}25.568\\ ***\end{tabular} & \begin{tabular}[c]{@{}l@{
## \hline
## \bottomrule
## \end{tabular}
## \caption{Regression results with controls of the within-class experiment. Dependent variables are sp
## \label{table:CountingDays_Within_Class_Experiment_Regression_Results_with_controls}
## \end{table*}
```

Table D2 - Beta regression results of the within-class experiment. The regression results without controls are included as a robustness check.

```r
# {table:CountingDays_Within_Class_Experiment_Regression_Results_reduced}
# List of terms based on actual term names
terms_order <- c(
  "SpacingCounting Days",
  "GPA",
  "SpacingCounting Days:GPA",
  "(Intercept)",
  "Parameter"
)
```

```r
# Corresponding labels
terms_labels <- c(
  "\\begin{tabular}[c]{@{}l@{}}\\textbf{Counting Days}\\\\ \\textbf{(vs. Questions)}\\end{tabular}",
  "\\textbf{GPA}",
  "\\begin{tabular}[c]{@{}l@{}}\\textbf{Counting Days}\\\\ \\textbf{$\\times$ GPA}\\end{tabular}",
  "\\textbf{[Intercept]}",
  "\\textbf{[Parameter]}"
)

# Header of the table
header <- "\\begin{table*}[!htp]
\\centering
\\ra{1.21}
\\begin{tabular}{@{}p{3.1cm}lll@{}}
\\toprule
\\multicolumn{1}{c}{\\textbf{}} & \\textbf{Final} & \\textbf{Days} & \\textbf{Questions} \\\\
\\multicolumn{1}{c}{\\textbf{}} & \\textbf{Exam Score} & \\textbf{Practiced} & \\textbf{Practiced} \\\\
\\multicolumn{1}{l}{\\textbf{}} & \\textbf{(Beta)} & \\textbf{(Neg. Bin.)} & \\textbf{(Neg. Bin.)} \\\\
\\midrule
"

# Footer of the table
footer <- "\\bottomrule
\\end{tabular}
\\caption{Beta regression results of the within-class experiment. The regression results without control
\\label{table:CountingDays_Within_Class_Experiment_Regression_Results_reduced}
\\end{table*}
"

# Generate the LaTeX code for the table
generate_latex_table_of_three_models(
  Final_Model_No_Controls,
  "betareg",
  Practice_Days_Model_No_Controls,
  "glm.nb",
  Practice_Count_Model_No_Controls,
  "glm.nb",
  header,
  footer,
  terms_order,
  terms_labels
  )
```

```
## \begin{table*}[!htp]
## \centering
## \ra{1.21}
## \begin{tabular}{@{}p{3.1cm}lll@{}}
## \toprule
## \multicolumn{1}{c}{\textbf{}} & \textbf{Final} & \textbf{Days} & \textbf{Questions} \\
## \multicolumn{1}{c}{\textbf{}} & \textbf{Exam Score} & \textbf{Practiced} & \textbf{Practiced} \\
## \multicolumn{1}{l}{\textbf{}} & \textbf{(Beta)} & \textbf{(Neg. Bin.)} & \textbf{(Neg. Bin.)} \\
## \midrule
## \begin{tabular}[c]{@{}l@{}}\textbf{Counting Days}\\ \textbf{(vs. Questions)}\end{tabular} & \begin{ta
## \hline
```

```
## \textbf{GPA} & \begin{tabular}[c]{@{}l@{}}0.657***\\ (0.161)\end{tabular} & \begin{tabular}[c]{@{}l@
## \hline
## \begin{tabular}[c]{@{}l@{}}\textbf{Counting Days}\\ \textbf{$\times$ GPA}\end{tabular} & \begin{tabu
## \hline
## \textbf{[Intercept]} & \begin{tabular}[c]{@{}l@{}}-0.713\\ (0.534)\end{tabular} & \begin{tabular}[c]
## \hline
## \textbf{[Parameter]} & \begin{tabular}[c]{@{}l@{}}\textbf{Scale:}\\ 13.573***\\ (1.594)\end{tabular}
## \hline
## \textbf{Log Likelihood} & 144.049 & -480.101 & -779.858 \\
## \hline
## \textbf{\# of Obs.} & 143 & 143 & 143 \\
## \hline
## \textbf{$\chi^2$} & \begin{tabular}[c]{@{}l@{}}10.485\\ ***\end{tabular} & \begin{tabular}[c]{@{}l@{
## \hline
## \bottomrule
## \end{tabular}
## \caption{Beta regression results of the within-class experiment. The regression results without cont
## \label{table:CountingDays_Within_Class_Experiment_Regression_Results_reduced}
## \end{table*}
```

Table D3 - Regression results with controls of the within-class experiment, including pass-fail students.

```r
# {table:beta_with_pass_fail}
# List of terms based on actual term names
terms_order <- c(
  "SpacingCounting Days",
  "GPA",
  "SpacingCounting Days:GPA",
  "Female1",
  "Asian1",
  "NonWhiteOrAsian1",
  "Junior1",
  "Senior1",
  "NonNativeEnglish1",
  "(Intercept)",
  "Parameter"
)

# Corresponding labels
terms_labels <- c(
  "\\begin{tabular}[c]{@{}l@{}}\\textbf{Counting Days}\\\\ \\textbf{(vs. Questions)}\\end{tabular}",
  "\\textbf{GPA}",
  "\\begin{tabular}[c]{@{}l@{}}\\textbf{Counting Days}\\\\ \\textbf{$\\times$ GPA}\\end{tabular}",
  "\\begin{tabular}[c]{@{}l@{}}\\textbf{Female}\\\\ \\textbf{(vs. Male)}\\end{tabular}",
  "\\begin{tabular}[c]{@{}l@{}}\\textbf{Asian}\\\\ \\textbf{(vs. White)}\\end{tabular}",
  "\\begin{tabular}[c]{@{}l@{}}\\textbf{NonWhiteOrAsian}\\\\ \\textbf{(vs. White)}\\end{tabular}",
  "\\begin{tabular}[c]{@{}l@{}}\\textbf{Junior}\\\\ \\textbf{(vs. Sophomore)}\\end{tabular}",
  "\\begin{tabular}[c]{@{}l@{}}\\textbf{Senior}\\\\ \\textbf{(vs. Sophomore)}\\end{tabular}",
  "\\begin{tabular}[c]{@{}l@{}}\\textbf{NonNativeEnglish}\\\\ \\textbf{(vs. NativeEnglish)}\\end{tabula
  "\\textbf{[Intercept]}",
  "\\textbf{[Parameter]}"
)
```

```r
# Header of the table
header <- "\\begin{table*}[!htp]
\\centering
\\ra{1.21}
\\begin{tabular}{@{}p{3.1cm}lll@{}}
\\toprule
\\multicolumn{1}{c}{\\textbf{}} & \\textbf{Final} & \\textbf{Days} & \\textbf{Questions} \\\\
\\multicolumn{1}{c}{\\textbf{}} & \\textbf{Exam Score} & \\textbf{Practiced} & \\textbf{Practiced} \\\\
\\multicolumn{1}{l}{\\textbf{}} & \\textbf{(Beta)} & \\textbf{(Neg. Bin.)} & \\textbf{(Neg. Bin.)} \\\\
\\midrule
"

# Footer of the table
footer <- "\\bottomrule
\\end{tabular}
\\caption{Regression results with controls of the within-class experiment, \\emph{including pass-fail s
\\label{table:beta_with_pass_fail}
\\end{table*}
"

# Generate the LaTeX code for the table
generate_latex_table_of_three_models(
  Final_Model_All_Students,
  "betareg",
  Practice_Days_Model_All_Students,
  "glm.nb",
  Practice_Count_Model_All_Students,
  "glm.nb",
  header,
  footer,
  terms_order,
  terms_labels
  )
```

```
## \begin{table*}[!htp]
## \centering
## \ra{1.21}
## \begin{tabular}{@{}p{3.1cm}lll@{}}
## \toprule
## \multicolumn{1}{c}{\textbf{}} & \textbf{Final} & \textbf{Days} & \textbf{Questions} \\
## \multicolumn{1}{c}{\textbf{}} & \textbf{Exam Score} & \textbf{Practiced} & \textbf{Practiced} \\
## \multicolumn{1}{l}{\textbf{}} & \textbf{(Beta)} & \textbf{(Neg. Bin.)} & \textbf{(Neg. Bin.)} \\
## \midrule
## \begin{tabular}[c]{@{}l@{}}\textbf{Counting Days}\\ \textbf{(vs. Questions)}\end{tabular} & \begin{ta
## \hline
## \textbf{GPA} & \begin{tabular}[c]{@{}l@{}}0.604***\\ (0.165)\end{tabular} & \begin{tabular}[c]{@{}l@
## \hline
## \begin{tabular}[c]{@{}l@{}}\textbf{Counting Days}\\ \textbf{$\times$ GPA}\end{tabular} & \begin{tabu
## \hline
## \begin{tabular}[c]{@{}l@{}}\textbf{Female}\\ \textbf{(vs. Male)}\end{tabular} & \begin{tabular}[c]{@
## \hline
## \begin{tabular}[c]{@{}l@{}}\textbf{Asian}\\ \textbf{(vs. White)}\end{tabular} & \begin{tabular}[c]{@
## \hline
```

```
## \begin{tabular}[c]{@{}l@{}}\textbf{NonWhiteOrAsian}\\ \textbf{(vs. White)}\end{tabular} & \begin{tabu
## \hline
## \begin{tabular}[c]{@{}l@{}}\textbf{Junior}\\ \textbf{(vs. Sophomore)}\end{tabular} & \begin{tabular}
## \hline
## \begin{tabular}[c]{@{}l@{}}\textbf{Senior}\\ \textbf{(vs. Sophomore)}\end{tabular} & \begin{tabular}
## \hline
## \begin{tabular}[c]{@{}l@{}}\textbf{NonNativeEnglish}\\ \textbf{(vs. NativeEnglish)}\end{tabular} & \
## \hline
## \textbf{[Intercept]} & \begin{tabular}[c]{@{}l@{}}-0.789\\ (0.566)\end{tabular} & \begin{tabular}[c]
## \hline
## \textbf{[Parameter]} & \begin{tabular}[c]{@{}l@{}}\textbf{Scale:}\\ 11.692***\\ (1.307)\end{tabular}
## \textbf{Log Likelihood} & 144.452 & -525.984 & -938.327 \\
## \textbf{\# of Obs.} & 157 & 157 & 157 \\
## \textbf{$\chi^2$} & \begin{tabular}[c]{@{}l@{}}17.941\\ ***\end{tabular} & \begin{tabular}[c]{@{}l@{
## \hline
## \bottomrule
## \end{tabular}
## \caption{Regression results with controls of the within-class experiment, \emph{including pass-fail
## \label{table:beta_with_pass_fail}
## \end{table*}
```

Fig. F2

Note that we do not have GSEM capabilities in R and the following regression results may not be reliable. So, although very similar, we rely on the results of the GSEM analysis in STATA.

```
Final_Practice_Days_Count_Model <- betareg(Final_0_1 ~ Practice_Days + Practice_Count +
GPA + Female + Asian + NonWhiteOrAsian +
Junior + Senior + NonNativeEnglish,
data = data)
vif(Final_Practice_Days_Count_Model)
```

```
##    Practice_Days   Practice_Count              GPA
##      1.320220183      1.361887658      1.273205867
##           Female            Asian  NonWhiteOrAsian
##      1.259405272      1.172814060      1.082025636
##           Junior           Senior NonNativeEnglish
##      1.154235578      1.137071391      1.132256651
```

```
Final_Practice_Days_Count_Model <- betareg(Final_0_1 ~ (Practice_Days + Practice_Count) *
GPA +
Female + Asian + NonWhiteOrAsian + Junior +
Senior + NonNativeEnglish, data = data)
summary(Final_Practice_Days_Count_Model)
```

```
##
## Call:
## betareg(formula = Final_0_1 ~ (Practice_Days + Practice_Count) *
##     GPA + Female + Asian + NonWhiteOrAsian + Junior +
##     Senior + NonNativeEnglish, data = data)
##
## Quantile residuals:
##        Min         1Q     Median         3Q        Max
## -3.8040573 -0.5874842  0.0765136  0.6990938  2.8654517
##
## Coefficients (mean model with logit link):
```

```
##                          Estimate    Std. Error  z value
## (Intercept)        -1.1009628165  2.5643004627 -0.42934
## Practice_Days       0.0878281171  0.0391305956  2.24449
## Practice_Count     -0.0036339389  0.0073493705 -0.49446
## GPA                 0.8443290490  0.7971484405  1.05919
## Female1            -0.1491579993  0.1244939931 -1.19811
## Asian1              0.1216250096  0.1405047462  0.86563
## NonWhiteOrAsian1   -0.0259599803  0.1459054362 -0.17792
## Junior1             0.3134381562  0.1493381606  2.09885
## Senior1             0.3914816707  0.1495615473  2.61753
## NonNativeEnglish1   0.3397255463  0.1323148961  2.56755
## Practice_Days:GPA  -0.0236531478  0.0114798207 -2.06041
## Practice_Count:GPA  0.0007028901  0.0022460982  0.31294
##                     Pr(>|z|)
## (Intercept)        0.6676741
## Practice_Days      0.0248011 *
## Practice_Count     0.6209843
## GPA                0.2895147
## Female1            0.2308726
## Asian1             0.3866935
## NonWhiteOrAsian1   0.8587832
## Junior1            0.0358303 *
## Senior1            0.0088569 **
## NonNativeEnglish1  0.0102419 *
## Practice_Days:GPA  0.0393593 *
## Practice_Count:GPA 0.7543276
##
## Phi coefficients (precision model with identity link):
##        Estimate Std. Error z value   Pr(>|z|)
## (phi) 16.108097   1.896575 8.49326 < 2.22e-16 ***
## ---
## Signif. codes:  0 '***' 0.001 '**' 0.01 '*' 0.05 '.' 0.1 ' ' 1
##
## Type of estimator: ML (maximum likelihood)
## Log-likelihood: 155.8676 on 13 Df
## Pseudo R-squared: 0.2541658
## Number of iterations: 25 (BFGS) + 1 (Fisher scoring)
```

```
quantile(data$Practice_Days, c(0:3/3))
```

```
##          0%   33.33333%   66.66667%        100%
##  4.00000000 22.33333333 39.00000000 48.00000000
```

Figure F2

Predictive Margins of Days Practiced with 95% CIs for the within-class experiment. Estimated effect of GPA on final exam scores for different numbers of days practiced.

```
# {fig:CountingDays_Final_Exam_Practice_Days}
Final_Practice_Days_Model <- plot_model(Final_Practice_Days_Count_Model, type = "eff",
                                        terms = c("GPA", "Practice_Days [0,21,39,49]"),
                                        show.data = TRUE, value.size = 4, title = "") +
  scale_x_continuous(limits = c(2, 4)) +
  scale_y_continuous(limits = c(0.16, 1), labels=scales::percent) +
  geom_vline(xintercept=FA2018_GPA_Terciles[2]) +
```

```r
  geom_vline(xintercept=FA2018_GPA_Terciles[3]) +
  annotate("rect", xmin=2, xmax=FA2018_GPA_Terciles[2], ymin=0.16, ymax=0.37, alpha=0.2,
  fill="orange") +
  annotate("rect", xmin=FA2018_GPA_Terciles[2], xmax=FA2018_GPA_Terciles[3], ymin=0.16,
  ymax=0.37, alpha=0.2, fill="yellow") +
  annotate("rect", xmin=FA2018_GPA_Terciles[3], xmax=FA2018_GPA_Terciles[4], ymin=0.16,
  ymax=0.37, alpha=0.2, fill="green") +
  annotate("text", x = 2.71, y = 0.28, label = "Low GPA") +
  annotate("text", x = 2.71, y = 0.22, label = "1st Tercile") +
  annotate("text", x = 3.535, y = 0.28, label = "Mid GPA") +
  annotate("text", x = 3.535, y = 0.22, label = "2nd Tercile") +
  annotate("text", x = 3.85, y = 0.28, label = "High GPA") +
  annotate("text", x = 3.85, y = 0.22, label = "3rd Tercile") +
  scale_fill_gradientn(colours = c("tomato1", "purple", "blue"),
  values = c(1.0, 0.7, 0)) +
  scale_color_gradientn(colours = c("tomato1", "purple", "blue"),
  values = c(1.0, 0.7, 0)) +
  labs(
  x = "Fall 2018 - GPA",
  y = "Final Exam Score") +
  theme(
  legend.position = "top",
  text = element_text(size=16)
)
```

```
## Data points may overlap. Use the `jitter` argument to
##   add some amount of random variation to the location of
##   data points and avoid overplotting.
```

```
## Scale for y is already present.
## Adding another scale for y, which will replace the existing
## scale.
## Scale for colour is already present.
## Adding another scale for colour, which will replace the
## existing scale.
```

```r
Final_Practice_Days_Model
```

```
## Warning: Removed 3 rows containing missing values or values outside
## the scale range (`geom_point()`).
```

```
## Warning: Removed 4 rows containing missing values or values outside
## the scale range (`geom_line()`).
```

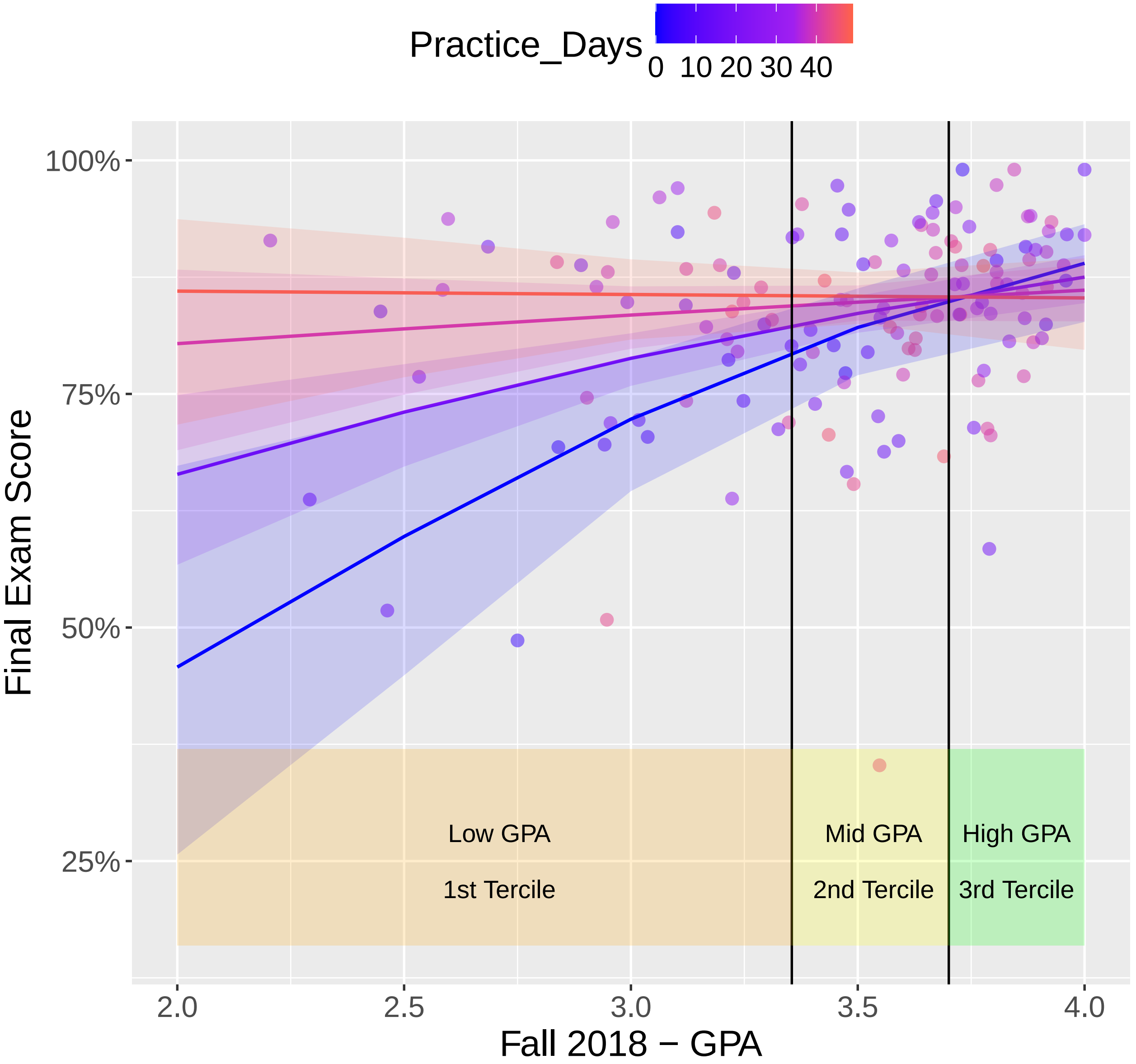

Daily Practice Analysis

Only consider the data collected in the experiment semester, Fall 2018.

```
daily_data <- subset(all_data, Term == "FA 2018")
```

The GPA field was populated from an inner join query with student data from the registrar. GPA field is blank when the student was not registered for this course in this semester (i.e., TAs and other system testers).

```
daily_data <- subset(daily_data, !is.na(GPA) & GPA != 0)
```

Exclude those students who did not take the final exam; i.e., withdrew the course (at the end of the semester, after paying the tuition) or failed.

```
daily_data <- subset(daily_data, !is.na(Final) & Final != 0)
```

Exclude those students who took the course for purposes other than getting a letter grade, such as auditing.

```r
daily_data <- subset(daily_data, GradingType == "Graded")
```

Calculate the difference in the daily number of questions practiced across the counting days and counting questions conditions.

```r
daily_comparison_data <- daily_data %>%
  mutate(Counting_Days_practice = ifelse(Spacing == "Counting Days", 1, 0),
         Counting_Questions_practice = ifelse(Spacing == "Counting Days", 0, 1)) %>%
  group_by(PracticeDate) %>%
  summarise(PracticeCountDiff = (sum(Counting_Days_practice) / n_distinct(X)) -
            (sum(Counting_Questions_practice) / n_distinct(X)))
```

Figure 3

Comparing procrastination patterns between students in the counting days and counting questions conditions in the within-class experiment. Each dot represents the daily difference between the average number of questions answered by students in the counting days and counting questions groups. Dots above the red line indicate that more questions were answered, on average, in the counting days than in the counting questions condition. The blue line shows the midterm exam date and the green line the final exam date.

```r
ggplot(daily_comparison_data) +
  geom_point(aes(x=PracticeDate, y=PracticeCountDiff)) +
  labs(
  x = 'More Answered in Counting Questions Condition',
  y = 'Difference in # of Questions Answered') +
  ggtitle("More Answered in Counting Days Condition") +
  geom_hline(yintercept = 0, color = "red") +
  geom_vline(xintercept = as.Date("2018-10-29"), color = "blue") +
  geom_vline(xintercept = as.Date("2018-12-19"), color = "green") +
  theme(
  text = element_text(size=19)
  )
```

```
## Warning: Removed 1 row containing missing values or values outside
## the scale range (`geom_point()`).
```

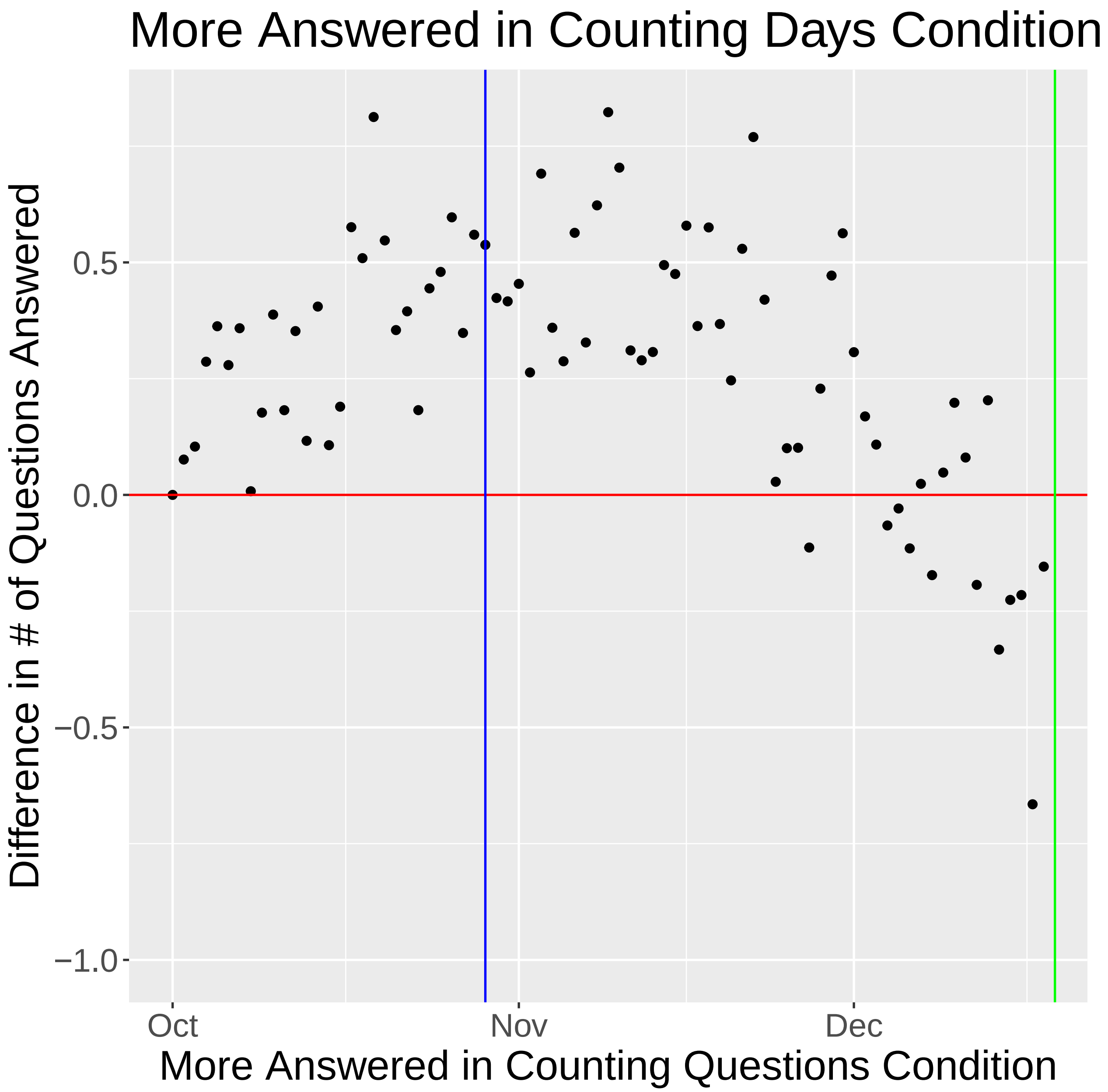

Four-semester Data Analysis

For Fall 2018, we should only include the counting days group.

```
four_semester_data <- all_data
```

Apply the data inclusion criteria to generate the student funnel

Generate the student-level dataframe. Each row should represent a single student.

```
four_semester_data <- four_semester_data[four_semester_data$Q > 0, ] %>%
  group_by(User_ID) %>%
  summarize(
    Term = Term[1],
```

```r
    GradingType = GradingType[1],
    Final = Final[1],
    Final_0_1 = Final_0_1[1],
    GPA = GPA[1],
    Sex = Sex[1],
    Male = Male[1],
    Female = Female[1],
    Ethnicity = Ethnicity[1],
    White = White[1],
    Asian = Asian[1],
    NonWhiteOrAsian = NonWhiteOrAsian[1],
    NonNativeEnglish = NonNativeEnglish[1],
    Freshman = Freshman[1],
    Junior = Junior[1],
    Sophomore = Sophomore[1],
    Senior = Senior[1],
    AcademicLevel = AcademicLevel[1],
    Practice_Count = n(),
    Practice_Time = sum(PracticeMinutes) / 60,
    Practice_Days = n_distinct(PracticeDate))
```

The GPA field was populated from an inner join query with student data from the registrar. GPA field is blank when the student was not registered for this course in this semester (i.e., TAs and other system testers).

```r
four_semester_data <- subset(four_semester_data, !is.na(four_semester_data$GPA) &
four_semester_data$GPA != 0)
```

After excluding those who have no GPA, a few Freshmen remained. We treat them as Sophomores because they had taken courses in the previous semester.

```r
four_semester_data$Sophomore <- factor(ifelse(four_semester_data$Freshman == 1 |
                                              four_semester_data$Sophomore == 1, 1, 0))
four_semester_data$AcademicLevel <- factor(ifelse(four_semester_data$Freshman == 1 |
                                                  four_semester_data$Sophomore == 1,
                                                  "Sophomore",
                                                  four_semester_data$AcademicLevel))
```

We exclude those students who did not take the final exam; i.e., withdrew the course (at the end of the semester, after paying the tuition) or failed.

```r
four_semester_data <- subset(four_semester_data, !is.na(four_semester_data$Final) &
                             four_semester_data$Final != 0)
```

We exclude those students who took the course for purposes other than getting a letter grade, such as auditing.

```r
four_semester_data <- subset(four_semester_data,
four_semester_data$GradingType == "Graded")
```

```r
WN2018_data <- subset(four_semester_data, Term=="WN 2018")
WN2018_GPA_Terciles <- quantile(WN2018_data$GPA, c(0:3/3))

WN2018_Model <- betareg(Final_0_1 ~ GPA + Female + Asian + NonWhiteOrAsian + Junior +
Senior + NonNativeEnglish, data = WN2018_data)
summary(WN2018_Model)
```

```
##
```

```
## Call:
## betareg(formula = Final_0_1 ~ GPA + Female + Asian +
##     NonWhiteOrAsian + Junior + Senior + NonNativeEnglish,
##     data = WN2018_data)
##
## Quantile residuals:
##        Min         1Q     Median         3Q        Max
## -1.8354146 -0.7175438 -0.1254896  0.7811536  3.1349072
##
## Coefficients (mean model with logit link):
##                     Estimate  Std. Error  z value
## (Intercept)       -1.51473837  0.60695277 -2.49564
## GPA                0.66681439  0.17429658  3.82575
## Female1           -0.12408284  0.13321878 -0.93142
## Asian1             0.25634394  0.15693953  1.63339
## NonWhiteOrAsian1  -0.07737357  0.18582664 -0.41638
## Junior1            0.08255238  0.16619507  0.49672
## Senior1            0.60208250  0.16599564  3.62710
## NonNativeEnglish1 -0.06904730  0.14297641 -0.48293
##                     Pr(>|z|)
## (Intercept)       0.01257285 *
## GPA               0.00013038 ***
## Female1           0.35163554
## Asian1            0.10238636
## NonWhiteOrAsian1  0.67713559
## Junior1           0.61938667
## Senior1           0.00028662 ***
## NonNativeEnglish1 0.62914690
##
## Phi coefficients (precision model with identity link):
##        Estimate Std. Error z value   Pr(>|z|)
## (phi) 12.158572   1.695537 7.17093 7.4492e-13 ***
## ---
## Signif. codes:  0 '***' 0.001 '**' 0.01 '*' 0.05 '.' 0.1 ' ' 1
##
## Type of estimator: ML (maximum likelihood)
## Log-likelihood: 68.95674 on 9 Df
## Pseudo R-squared: 0.2811442
## Number of iterations: 16 (BFGS) + 2 (Fisher scoring)
```

```r
WN2018_Plot <- plot_model(WN2018_Model, type = "eff", terms = c("GPA"), show.data = TRUE,
                          value.size = 4, title = "") +
  geom_vline(xintercept=WN2018_GPA_Terciles[2]) +
  geom_vline(xintercept=WN2018_GPA_Terciles[3]) +
  scale_x_continuous(limits = c(2, 4)) +
  scale_y_continuous(limits = c(0.16, 1), labels=scales::percent) +
  annotate("rect", xmin=2, xmax=WN2018_GPA_Terciles[2], ymin=0.16, ymax=0.37, alpha=0.2,
           fill="orange") +
  annotate("rect", xmin=WN2018_GPA_Terciles[2], xmax=WN2018_GPA_Terciles[3], ymin=0.16,
           ymax=0.37, alpha=0.2, fill="yellow") +
  annotate("rect", xmin=WN2018_GPA_Terciles[3], xmax=WN2018_GPA_Terciles[4], ymin=0.16,
           ymax=0.37, alpha=0.2, fill="green") +
  annotate("text", x = 2.71, y = 0.28, label = "Low GPA") +
  annotate("text", x = 2.71, y = 0.22, label = "1st Tercile") +
```

```r
  annotate("text", x = 3.454, y = 0.28, label = "Mid GPA") +
  annotate("text", x = 3.454, y = 0.22, label = "2nd Tercile") +
  annotate("text", x = 3.79, y = 0.28, label = "High GPA") +
  annotate("text", x = 3.79, y = 0.22, label = "3rd Tercile") +
  labs(
    x = "Winter 2018 - GPA",
    y = "") +
  theme(
    legend.position = "top",
    text = element_text(size=13),
  )
```

```
## Data points may overlap. Use the `jitter` argument to
##   add some amount of random variation to the location of
##   data points and avoid overplotting.
```

```
## Scale for y is already present.
## Adding another scale for y, which will replace the existing
## scale.
```

```r
WN2019_data <- subset(four_semester_data, Term=="WN 2019")
WN2019_GPA_Terciles <- quantile(WN2019_data$GPA, c(0:3/3))

WN2019_Model <- betareg(Final_0_1 ~ GPA + Female + Asian + NonWhiteOrAsian + Junior +
                        Senior + NonNativeEnglish, data = WN2019_data)
summary(WN2019_Model)
```

```
##
## Call:
## betareg(formula = Final_0_1 ~ GPA + Female + Asian +
##     NonWhiteOrAsian + Junior + Senior + NonNativeEnglish,
##     data = WN2019_data)
##
## Quantile residuals:
##        Min         1Q     Median         3Q        Max
## -2.8743517 -0.5910970 -0.0081177  0.7182177  2.2136856
##
## Coefficients (mean model with logit link):
##                     Estimate  Std. Error  z value
## (Intercept)       -2.40108935  0.87685925 -2.73828
## GPA                1.05135604  0.25195649  4.17277
## Female1           -0.57695993  0.21077511 -2.73732
## Asian1            -0.18593972  0.25936301 -0.71691
## NonWhiteOrAsian1   0.02873231  0.26081183  0.11016
## Junior1            0.36929090  0.26649378  1.38574
## Senior1            0.36889046  0.40825316  0.90358
## NonNativeEnglish1  0.11136858  0.23514443  0.47362
##                     Pr(>|z|)
## (Intercept)        0.0061761 **
## GPA               3.0092e-05 ***
## Female1            0.0061941 **
## Asian1             0.4734301
## NonWhiteOrAsian1   0.9122786
## Junior1            0.1658265
## Senior1            0.3662168
```

```
## NonNativeEnglish1  0.6357725
##
## Phi coefficients (precision model with identity link):
##       Estimate Std. Error z value Pr(>|z|)
## (phi) 7.004410   1.194488 5.86394 4.52e-09 ***
## ---
## Signif. codes:  0 '***' 0.001 '**' 0.01 '*' 0.05 '.' 0.1 ' ' 1
##
## Type of estimator: ML (maximum likelihood)
## Log-likelihood: 36.69042 on 9 Df
## Pseudo R-squared: 0.3265458
## Number of iterations: 16 (BFGS) + 2 (Fisher scoring)
```

```
WN2019_Plot <- plot_model(WN2019_Model, type = "eff", terms = c("GPA"), show.data = TRUE,
                          value.size = 4, title = "") +
  scale_y_continuous(limits = c(2, 4)) +
  geom_vline(xintercept=WN2019_GPA_Terciles[2]) +
  geom_vline(xintercept=WN2019_GPA_Terciles[3]) +
  scale_x_continuous(limits = c(2, 4)) +
  scale_y_continuous(limits = c(0.16, 1), labels=scales::percent) +
  annotate("rect", xmin=2, xmax=WN2019_GPA_Terciles[2], ymin=0.16, ymax=0.37, alpha=0.2,
           fill="orange") +
  annotate("rect", xmin=WN2019_GPA_Terciles[2], xmax=WN2019_GPA_Terciles[3], ymin=0.16,
           ymax=0.37, alpha=0.2, fill="yellow") +
  annotate("rect", xmin=WN2019_GPA_Terciles[3], xmax=WN2019_GPA_Terciles[4], ymin=0.16,
           ymax=0.37, alpha=0.2, fill="green") +
  annotate("text", x = 2.71, y = 0.28, label = "Low GPA") +
  annotate("text", x = 2.71, y = 0.22, label = "1st Tercile") +
  annotate("text", x = 3.46, y = 0.28, label = "Mid GPA") +
  annotate("text", x = 3.46, y = 0.22, label = "2nd Tercile") +
  annotate("text", x = 3.826, y = 0.28, label = "High GPA") +
  annotate("text", x = 3.826, y = 0.22, label = "3rd Tercile") +
  labs(
    x = "Winter 2019 - GPA",
    y = "") +
  theme(
    legend.position = "top",
    text = element_text(size=13),
  )
```

```
## Data points may overlap. Use the `jitter` argument to
##   add some amount of random variation to the location of
##   data points and avoid overplotting.

## Scale for y is already present.
## Adding another scale for y, which will replace the existing
## scale.
## Scale for y is already present.
## Adding another scale for y, which will replace the existing
## scale.
```

```
FA2019_data <- subset(four_semester_data, Term=="FA 2019")
FA2019_GPA_Terciles <- quantile(FA2019_data$GPA, c(0:3/3))

FA2019_Model <- betareg(Final_0_1 ~ GPA + Female + Asian + NonWhiteOrAsian + Junior +
                          Senior + NonNativeEnglish, data = FA2019_data)
```

```r
summary(FA2019_Model)
```

```
##
## Call:
## betareg(formula = Final_0_1 ~ GPA + Female + Asian +
##     NonWhiteOrAsian + Junior + Senior + NonNativeEnglish,
##     data = FA2019_data)
##
## Quantile residuals:
##        Min         1Q     Median         3Q        Max
## -2.7403123 -0.6491304  0.0631686  0.5184916  2.0554468
##
## Coefficients (mean model with logit link):
##                     Estimate Std. Error  z value  Pr(>|z|)
## (Intercept)        2.1425399  1.1158870  1.92003 0.0548537
## GPA               -0.3246051  0.3164549 -1.02575 0.3050072
## Female1            0.3617716  0.2363598  1.53060 0.1258691
## Asian1            -0.1898362  0.3505263 -0.54157 0.5881115
## NonWhiteOrAsian1  -0.2810990  0.2798080 -1.00461 0.3150827
## Junior1            1.4711285  0.6935248  2.12123 0.0339021
## Senior1            1.3015828  0.3452965  3.76946 0.0001636
## NonNativeEnglish1 -0.4429366  0.2559750 -1.73039 0.0835606
##
## (Intercept)       .
## GPA
## Female1
## Asian1
## NonWhiteOrAsian1
## Junior1           *
## Senior1           ***
## NonNativeEnglish1 .
##
## Phi coefficients (precision model with identity link):
##        Estimate Std. Error z value   Pr(>|z|)
## (phi) 10.295139   2.252447 4.57065 4.8622e-06 ***
## ---
## Signif. codes:  0 '***' 0.001 '**' 0.01 '*' 0.05 '.' 0.1 ' ' 1
##
## Type of estimator: ML (maximum likelihood)
## Log-likelihood: 33.63576 on 9 Df
## Pseudo R-squared: 0.3709205
## Number of iterations: 17 (BFGS) + 1 (Fisher scoring)
```

```r
FA2019_Plot <- plot_model(FA2019_Model, type = "eff", terms = c("GPA"), show.data = TRUE,
                          value.size = 4, title = "") +
  scale_y_continuous(limits = c(2, 4)) +
  geom_vline(xintercept=FA2019_GPA_Terciles[2]) +
  geom_vline(xintercept=FA2019_GPA_Terciles[3]) +
  scale_x_continuous(limits = c(2, 4)) +
  scale_y_continuous(limits = c(0.16, 1), labels=scales::percent) +
  annotate("rect", xmin=2, xmax=FA2019_GPA_Terciles[2], ymin=0.16, ymax=0.37, alpha=0.2,
           fill="orange") +
  annotate("rect", xmin=FA2019_GPA_Terciles[2], xmax=FA2019_GPA_Terciles[3], ymin=0.16,
           ymax=0.37, alpha=0.2, fill="yellow") +
```

```
  annotate("rect", xmin=FA2019_GPA_Terciles[3], xmax=FA2019_GPA_Terciles[4], ymin=0.16,
           ymax=0.37, alpha=0.2, fill="green") +
  annotate("text", x = 2.71, y = 0.28, label = "Low GPA") +
  annotate("text", x = 2.71, y = 0.22, label = "1st Tercile") +
  annotate("text", x = 3.49, y = 0.28, label = "Mid GPA") +
  annotate("text", x = 3.49, y = 0.22, label = "2nd Tercile") +
  annotate("text", x = 3.82, y = 0.28, label = "High GPA") +
  annotate("text", x = 3.82, y = 0.22, label = "3rd Tercile") +
  labs(
    x = "Fall 2019 - GPA",
    y = "") +
  theme(
    legend.position = "top",
    text = element_text(size=13),
  )
```

```
## Data points may overlap. Use the `jitter` argument to
##   add some amount of random variation to the location of
##   data points and avoid overplotting.

## Scale for y is already present.
## Adding another scale for y, which will replace the existing
## scale.
## Scale for y is already present.
## Adding another scale for y, which will replace the existing
## scale.
```

Figures G3, G4, G5

```
# {fig:CountingDays_Winter_2018}
# Adjusted Predictions with 95\% CIs in Winter 2018. Estimated effect of GPA on final exam scores based
WN2018_Plot
```

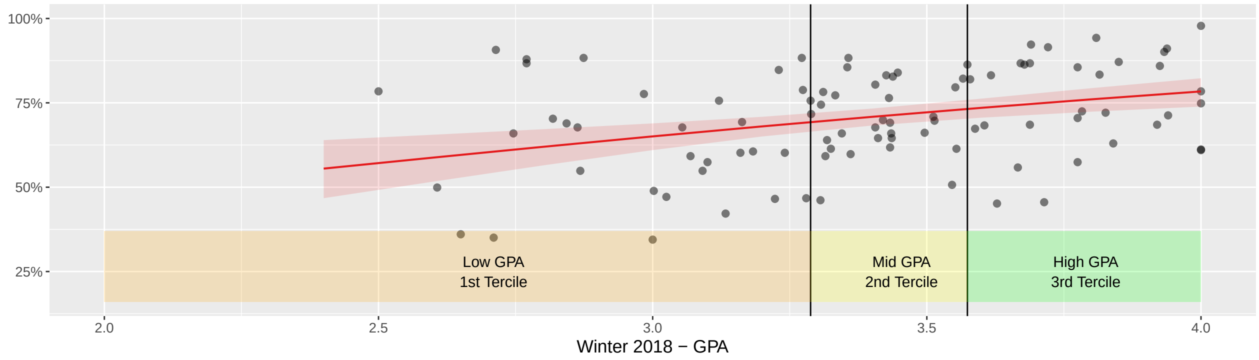


```
# {fig:CountingDays_Winter_2019}
# Adjusted Predictions with 95\% CIs in Winter 2019. Estimated effect of GPA on final exam scores based
WN2019_Plot
```

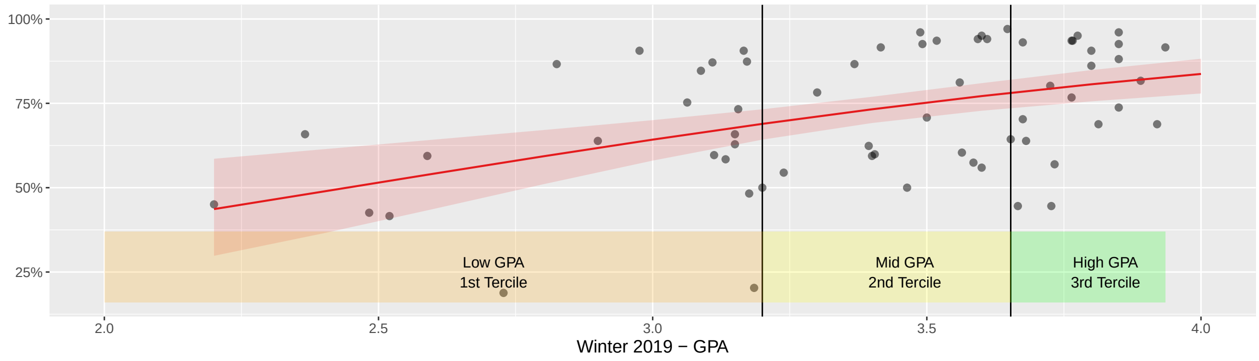


```
# {fig:CountingDays_Beta_Regression_GPA_Effect_on_Final_Exam_Scores}
# Adjusted Predictions with 95\% CIs in Fall 2019. Estimated effect of GPA on final exam scores based o
FA2019_Plot
```

```
## Warning: Removed 1 row containing missing values or values outside
## the scale range (`geom_point()`).

## Warning: Removed 1 row containing missing values or values outside
## the scale range (`geom_line()`).
```

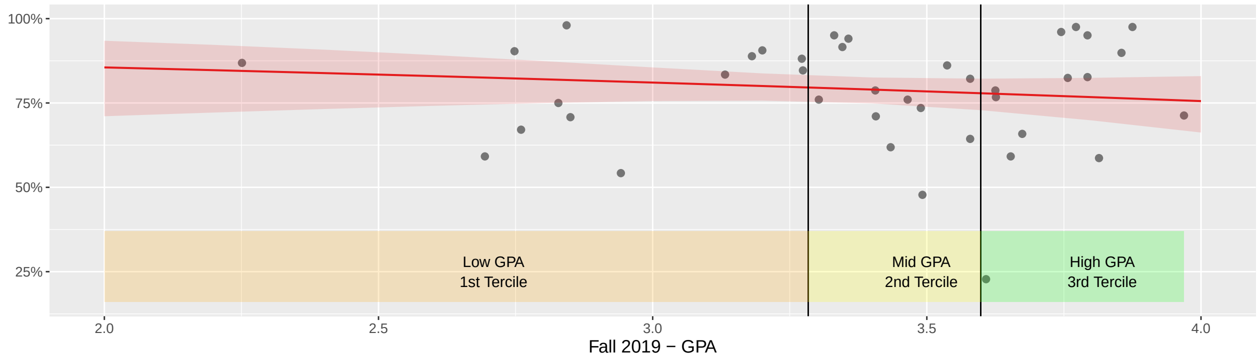

Between-instructor Experiment Data Analysis

Loading the data.

```
between_instructor_data <- read.csv("Between-Instructor Experiment Dataset.csv")
between_instructor_data$Course_Name <- factor(between_instructor_data$Course_Name)
between_instructor_data$Instructor <- factor(between_instructor_data$Instructor)
between_instructor_data$User_ID <- factor(between_instructor_data$User_ID)
between_instructor_data$Spacing <- factor(between_instructor_data$Spacing)
between_instructor_data$Spacing <- relevel(between_instructor_data$Spacing,
                                            ref = "Counting Questions")
```

Five instructors, three under the Counting Questions condition, and two under the Counting Days condition created test accounts and tried opening the practice tool before setting up the sections of the textbook to be practiced. That is indicated by the number 1 value for the Practice_Days variable while Practice_Count having the value 0. We excluded these records from our analysis.

```
subset(between_instructor_data, Practice_Count == 0 & Practice_Days == 1)
```

```
##          X Course_Name Instructor User_ID            Spacing
## 278    278          15      97650  251436 Counting Questions
## 524    524          27      38527  136284 Counting Questions
```

```
## 614   614           31      20117  300204 Counting Questions
## 1217 1217           69      52287  196847      Counting Days
## 1364 1364           79         24  167338      Counting Days
##      Practice_Days Practice_Count max_practice_days
## 278              1              0                50
## 524              1              0                50
## 614              1              0                50
## 1217             1              0                50
## 1364             1              0                60
##      max_practice_questions day_points
## 278                     500          2
## 524                   10000          2
## 614                     500          2
## 1217                    500          2
## 1364                    500          5
##      questions_to_complete_day students question_points
## 278                         10       10             0.2
## 524                         10       11             0.2
## 614                         10       30             0.2
## 1217                         5        8             0.2
## 1364                         5       28             0.2
```

```
between_instructor_data <- subset(between_instructor_data, Practice_Count != 0 | Practice_Days != 1)
```

2.2 Participants

Experiment 2: Between-Instructors

Report the number of instructors, courses, and students under the experimental conditions.

```
instructor_conditions <- between_instructor_data %>%
  group_by(Spacing) %>%
  summarise(
    instructors = n_distinct(Instructor),
    courses = n_distinct(Course_Name),
    students = n()
  )
instructor_conditions
```

```
## # A tibble: 2 x 4
##   Spacing            instructors courses students
##   <fct>                    <int>   <int>    <int>
## 1 Counting Questions          27      39      654
## 2 Counting Days               44      58      994
```

Table 1

Summary statistics for student counts and instructor configurations in between-instructors experiment.

```
# {table:Between_Instructor_Statistics_Student_Counts_Instructor_Configurations}
# Ensure that 'Spacing' is treated as a factor variable
the_dataframe <- between_instructor_data %>%
  mutate(
    spacing = ifelse(Spacing == "Counting Days", 1, 0),
    Condition = ifelse(spacing == 1, "Counting Days", "Counting Questions"),
    # Convert relevant variables to numeric
```

```
    max_practice_days = as.numeric(max_practice_days),
    max_practice_questions = as.numeric(max_practice_questions),
    day_points = as.numeric(day_points),
    questions_to_complete_day = as.numeric(questions_to_complete_day),
    students = as.numeric(students),
    practice_count = as.numeric(Practice_Count),
    practice_days = as.numeric(Practice_Days),
    question_points = as.numeric(question_points)
  )

# Compute summary statistics for each variable
stats_students <- the_dataframe %>%
  group_by(Condition) %>%
  summarise(
    Variable = "\\# of Students",
    Min = min(students, na.rm = TRUE),
    Median = median(students, na.rm = TRUE),
    Mean = mean(students, na.rm = TRUE),
    Max = max(students, na.rm = TRUE),
    SD = sd(students, na.rm = TRUE),
    .groups = 'drop'
  )

stats_max_practice_days <- the_dataframe %>%
  filter(Condition == "Counting Days") %>%
  summarise(
    Variable = "Max \\# of Days to Earn Points",
    Condition = "Counting Days",
    Min = min(max_practice_days, na.rm = TRUE),
    Median = median(max_practice_days, na.rm = TRUE),
    Mean = mean(max_practice_days, na.rm = TRUE),
    Max = max(max_practice_days, na.rm = TRUE),
    SD = sd(max_practice_days, na.rm = TRUE)
  )

stats_day_points <- the_dataframe %>%
  filter(Condition == "Counting Days") %>%
  summarise(
    Variable = "Points Per Day of Practice",
    Condition = "Counting Days",
    Min = min(day_points, na.rm = TRUE),
    Median = median(day_points, na.rm = TRUE),
    Mean = mean(day_points, na.rm = TRUE),
    Max = max(day_points, na.rm = TRUE),
    SD = sd(day_points, na.rm = TRUE)
  )

stats_questions_to_complete_day <- the_dataframe %>%
  filter(Condition == "Counting Days") %>%
  summarise(
    Variable = "\\# of Questions to Earn Day's Points",
    Condition = "Counting Days",
    Min = min(questions_to_complete_day, na.rm = TRUE),
```

```
    Median = median(questions_to_complete_day, na.rm = TRUE),
    Mean = mean(questions_to_complete_day, na.rm = TRUE),
    Max = max(questions_to_complete_day, na.rm = TRUE),
    SD = sd(questions_to_complete_day, na.rm = TRUE)
  )

stats_question_points <- the_dataframe %>%
  filter(Condition == "Counting Questions") %>%
  summarise(
    Variable = "Points Per Question Practiced",
    Condition = "Counting Questions",
    Min = min(question_points, na.rm = TRUE),
    Median = median(question_points, na.rm = TRUE),
    Mean = mean(question_points, na.rm = TRUE),
    Max = max(question_points, na.rm = TRUE),
    SD = sd(question_points, na.rm = TRUE)
  )

stats_max_practice_questions <- the_dataframe %>%
  filter(Condition == "Counting Questions") %>%
  summarise(
    Variable = "Max \\# of Questions",
    Condition = "Counting Questions",
    Min = min(max_practice_questions, na.rm = TRUE),
    Median = median(max_practice_questions, na.rm = TRUE),
    Mean = mean(max_practice_questions, na.rm = TRUE),
    Max = max(max_practice_questions, na.rm = TRUE),
    SD = sd(max_practice_questions, na.rm = TRUE)
  )

# Combine all statistics into one data frame
stats_combined <- bind_rows(
  stats_students,
  stats_max_practice_days,
  stats_day_points,
  stats_questions_to_complete_day,
  stats_question_points,
  stats_max_practice_questions
)

# Format numbers
stats_combined <- stats_combined %>%
  mutate(
    Mean = round(Mean, 2),
    SD = round(SD, 2),
    Max = ifelse(Variable %in% c("Max \\# of Questions"), comma(Max, accuracy = 1), Max),
    Max = ifelse(Variable == "\\# of Students", as.character(Max), Max),
    Max = ifelse(Variable == "Points Per Question Practiced", as.character(Max), Max),
    Min = as.character(Min),
    Median = as.character(Median),
    Max = as.character(Max)
  )
```

```r
# Prepare data for LaTeX table
# Manually create rows to match the desired table format
table_rows <- ""

# Helper function to add rows to the LaTeX table
add_variable_rows <- function(variable_name, data, multirow_rows = NULL, include_cmidrule = FALSE) {
  rows <- ""
  data_var <- data %>% filter(Variable == variable_name)

  if (nrow(data_var) > 0) {
    if (is.null(multirow_rows)) {
      multirow_rows <- ifelse(nrow(data_var) > 1, 2, 2)
    }
    # Start the multirow
    rows <- paste0("\\multirow{", multirow_rows, "}{2.5cm}{", variable_name, "} ")
    for (i in 1:nrow(data_var)) {
      row <- data_var[i, ]
      if (i == 1) {
        # First row with multirow
        rows <- paste0(rows, "& ", row$Condition, " & ", row$Min, " & ", row$Median,
                       " & ", row$Mean, " & ", row$Max, " & ", row$SD, " \\\\ \n")
        if (include_cmidrule) {
          rows <- paste0(rows, "\\cmidrule(lr){2-7}\n")
        }
      } else {
        # Subsequent rows
        rows <- paste0("& ", row$Condition, " & ", row$Min, " & ", row$Median,
                       " & ", row$Mean, " & ", row$Max, " & ", row$SD, " \\\\ ")
        if (i < nrow(data_var)) {
          rows <- paste0(rows, "\n")
        } else {
          rows <- paste0(rows, "\\midrule\n")
        }
      }
    }
    # Add empty rows if multirow_rows > nrow(data_var)
    if (multirow_rows > nrow(data_var)) {
      for (j in 1:(multirow_rows - nrow(data_var))) {
        rows <- paste0(rows, "& & & & & & \\\\ \n")
      }
      rows <- paste0(rows, "\\midrule\n")
    }
  } else {
    # Variable not found
    rows <- ""
  }
  return(rows)
}

# Build the LaTeX table rows
table_rows <- ""

# Handle the special case for \# of Students
```

```r
data_var <- stats_combined %>% filter(Variable == "\\# of Students")

if (nrow(data_var) == 2) {
  rows <- paste0("\\multirow{4}{2.5cm}{\\# of Students} & ", data_var$Condition[1], " & ", data_var$Min
                 " & ", data_var$Mean[1], " & ", data_var$Max[1], " & ", data_var$SD[1], " \\\\ \n")
  rows <- paste0(rows, "\\cmidrule(lr){2-7}\n")
  rows <- paste0(rows, "& ", data_var$Condition[2], " & ", data_var$Min[2], " & ", data_var$Median[2],
                 " & ", data_var$Mean[2], " & ", data_var$Max[2], " & ", data_var$SD[2], " \\\\ \\midrul
  table_rows <- paste0(table_rows, rows)
}

# For the other variables
table_rows <- paste0(table_rows, add_variable_rows("Max \\# of Days to Earn Points", stats_combined))
table_rows <- paste0(table_rows, add_variable_rows("Points Per Day of Practice", stats_combined))
table_rows <- paste0(table_rows, add_variable_rows("\\# of Questions to Earn Day's Points", stats_combi
table_rows <- paste0(table_rows, add_variable_rows("Points Per Question Practiced", stats_combined))
table_rows <- paste0(table_rows, add_variable_rows("Max \\# of Questions", stats_combined))

# Remove the last \\midrule
table_rows <- sub("\\\\midrule\n$", "", table_rows)

# Assemble the full LaTeX table
latex_table <- paste0("\\begin{table*}[!htp]
\\centering
\\ra{1.0}
\\begin{tabular}{@{}p{2.2cm}p{1.6cm}rrrrr@{}}
\\toprule
\\textbf{Variable} & \\textbf{Condition} & \\textbf{Min} & \\textbf{Median} & \\textbf{Mean} & \\textbf
", table_rows, "
\\bottomrule
\\end{tabular}
\\caption{Summary statistics for student counts and instructor configurations in between-instructors ex
\\label{table:Between_Instructor_Statistics_Student_Counts_Instructor_Configurations}
\\end{table*}")

# Output the LaTeX code
cat(latex_table)
```

```
## \begin{table*}[!htp]
## \centering
## \ra{1.0}
## \begin{tabular}{@{}p{2.2cm}p{1.6cm}rrrrr@{}}
## \toprule
## \textbf{Variable} & \textbf{Condition} & \textbf{Min} & \textbf{Median} & \textbf{Mean} & \textbf{Ma
## \multirow{4}{2.5cm}{\# of Students} & Counting Days & 5 & 19 & 25.92 & 84 & 20.68 \\
## \cmidrule(lr){2-7}
## & Counting Questions & 5 & 23 & 23.82 & 54 & 12.59 \\ \midrule
## \multirow{2}{2.5cm}{Max \# of Days to Earn Points} & Counting Days & 8 & 50 & 63.17 & 200 & 37.54 \\
## & & & & & & \\
## \midrule
## \multirow{2}{2.5cm}{Points Per Day of Practice} & Counting Days & 1 & 2 & 3.97 & 100 & 9.93 \\
## & & & & & & \\
## \midrule
## \multirow{2}{2.5cm}{\# of Questions to Earn Day's Points} & Counting Days & 2 & 7 & 7.47 & 20 & 2.87
```

```
## & & & & & & \\
## \midrule
## \multirow{2}{2.5cm}{Points Per Question Practiced} & Counting Questions & 0.1 & 0.2 & 0.44 & 2 & 0.3
## & & & & & & \\
## \midrule
## \multirow{2}{2.5cm}{Max \# of Questions} & Counting Questions & 50 & 500 & 630.99 & 10,000 & 1191.93
## & & & & & & \\
##
## \bottomrule
## \end{tabular}
## \caption{Summary statistics for student counts and instructor configurations in between-instructors
## \label{table:Between_Instructor_Statistics_Student_Counts_Instructor_Configurations}
## \end{table*}
```

Table 8

Summary statistics of both input and output variables and randomization check.

```r
# {table:Between_Instructor_Summary_Statistics}
# Compute summary statistics for Practice_Days
summary_days <- between_instructor_data %>%
  group_by(Condition = Spacing) %>%
  summarise(
    Variable = "\\# of Days Practiced Per Student",
    Min = min(Practice_Days),
    Median = median(Practice_Days),
    Mean = mean(Practice_Days),
    Max = max(Practice_Days),
    SD = sd(Practice_Days),
    .groups = 'keep'
  )

# Compute summary statistics for Practice_Count
summary_count <- between_instructor_data %>%
  group_by(Condition = Spacing) %>%
  summarise(
    Variable = "\\# of Questions Practiced Per Student",
    Min = min(Practice_Count),
    Median = median(Practice_Count),
    Mean = mean(Practice_Count),
    Max = max(Practice_Count),
    SD = sd(Practice_Count),
    .groups = 'keep'
  )

# Combine and format the summary statistics
summary_stats <- bind_rows(summary_days, summary_count) %>%
  # Order the Variables as desired
  mutate(
    Variable = factor(Variable, levels = c(
      "\\# of Days Practiced Per Student",
      "\\# of Questions Practiced Per Student"
    )),
    Condition = factor(Condition, levels = c("Counting Days", "Counting Questions"))
```

```
  ) %>%
  arrange(Variable, Condition) %>%
  # Format numbers
  mutate(
    Min = as.character(Min),
    Median = as.character(Median),
    Mean = sprintf("%.1f", Mean),
    Max = comma(Max),
    SD = sprintf("%.2f", SD)
  )

# Prepare the LaTeX code manually
latex_table <- "\\begin{table*}[!htp]
\\centering
\\ra{1.0}
\\begin{tabular}{@{}p{2.2cm}p{1.6cm}rrrrr@{}}
\\toprule
\\textbf{Variable} & \\textbf{Condition} & \\textbf{Min} & \\textbf{Median} & \\textbf{Mean} & \\textbf
"

# Add rows for each Variable
variables <- unique(summary_stats$Variable)
for (var_index in seq_along(variables)) {
  var <- variables[var_index]
  # Get the subset of data for this variable
  data_sub <- summary_stats %>% filter(Variable == var)
  # Start the multirow for the first variable
  latex_table <- paste0(latex_table, "\\multirow{2}{2.5cm}{", var, "} ")
  for (i in 1:nrow(data_sub)) {
    row <- data_sub[i, ]
    # For the first row, the multirow is already started
    if (i == 1) {
      latex_table <- paste0(latex_table, "& ", row$Condition, " & ", row$Min, " & ", row$Median,
                            " & ", row$Mean, " & ", row$Max, " & ", row$SD, " \\\\ \n")
    } else {
      # Second row without multirow
      latex_table <- paste0(latex_table, "& ", row$Condition, " & ", row$Min, " & ", row$Median,
                            " & ", row$Mean, " & ", row$Max, " & ", row$SD, " \\\\ \n")
    }
  }
  # After each variable block, except the last one, add \midrule
  if (var_index < length(variables)) {
    latex_table <- paste0(latex_table, "\\midrule\n")
  }
}

# Finish the table
latex_table <- paste0(latex_table, "\\bottomrule
\\end{tabular}
\\caption{Summary statistics of the outcome variables for between-instructors experiment.}
\\label{table:Between_Instructor_Summary_Statistics}
\\end{table*}")
```

```
# Output the LaTeX code
cat(latex_table)
```

```
## \begin{table*}[!htp]
## \centering
## \ra{1.0}
## \begin{tabular}{@{}p{2.2cm}p{1.6cm}rrrrr@{}}
## \toprule
## \textbf{Variable} & \textbf{Condition} & \textbf{Min} & \textbf{Median} & \textbf{Mean} & \textbf{Ma
## \multirow{2}{2.5cm}{\# of Days Practiced Per Student} & Counting Days & 1 & 7 & 17.4 & 254 & 21.52 \
## & Counting Questions & 1 & 6 & 12.0 & 80 & 14.58 \\
## \midrule
## \multirow{2}{2.5cm}{\# of Questions Practiced Per Student} & Counting Days & 1 & 100 & 217.0 & 2,971
## & Counting Questions & 1 & 104 & 197.4 & 1,040 & 209.75 \\
## \bottomrule
## \end{tabular}
## \caption{Summary statistics of the outcome variables for between-instructors experiment.}
## \label{table:Between_Instructor_Summary_Statistics}
## \end{table*}
```

Table 9

T-test and Two-sample Wilcoxon rank-sum (Mann–Whitney) test results for the between-instructor experiment (*** $p < 0.001$; ** $p < 0.01$; * $p < 0.05$).

```
# {table:CountingDays_Between_Instructor_T_Test_Wilcoxon_Rank_Sum}
# Reverse the levels of Spacing for the t.test.
between_instructor_data$Spacing_R <- factor(between_instructor_data$Spacing,
                                            levels = c("Counting Days",
                                                       "Counting Questions"))

# Initialize a data frame to store results
results <- data.frame(
  Variable = c("Practiced Days", "Practiced Questions"),
  T_test = character(2),
  Wilcoxon_test = character(2),
  stringsAsFactors = FALSE
)

# Function to format significance stars
get_significance_stars <- function(p_value) {
  if (p_value < 0.001) {
    return("***")
  } else if (p_value < 0.01) {
    return("**")
  } else if (p_value < 0.05) {
    return("*")
  } else {
    return("")
  }
}

# Perform tests for each variable
variables <- list(
```

```r
  list(name = "Practiced Days", var = "Practice_Days"),
  list(name = "Practiced Questions", var = "Practice_Count")
)

for (i in seq_along(variables)) {
  var_name <- variables[[i]]$name
  var <- variables[[i]]$var

  # T-test
  t_test <- t.test(as.formula(paste(var, "~ Spacing_R")),
                   data = between_instructor_data,
                   alternative = "greater",
                   var.equal = TRUE)
  t_value <- t_test$statistic
  p_value_t <- t_test$p.value
  stars_t <- get_significance_stars(p_value_t)
  t_stat_formatted <- paste0(sprintf("%.3f", t_value), stars_t)

  # Wilcoxon test using 'coin' package
  wilcox_test_result <- wilcox_test(
    as.formula(paste(var, "~ Spacing_R")),
    data = data,
    alternative = "less",
    distribution = "asymptotic"
  )
  z_value <- statistic(wilcox_test_result, type = "standardized")
  p_value_w <- pvalue(wilcox_test_result)
  stars_w <- get_significance_stars(p_value_w)
  wilcox_stat_formatted <- paste0(sprintf("%.3f", abs(z_value)), stars_w)

  # Store results
  results$T_test[results$Variable == var_name] <- t_stat_formatted
  results$Wilcoxon_test[results$Variable == var_name] <- wilcox_stat_formatted
}

# Prepare the LaTeX table code
latex_table <- "\\begin{table*}[!htp]
\\centering
\\ra{1.6}
\\begin{tabular}{@{}p{3.7cm}p{3.1cm}p{4.3cm}@{}}
\\toprule
& Two-sample t-test with equal variances & Two-sample Wilcoxon rank-sum (Mann--Whitney) test \\\\ \\mid
"

for (i in 1:nrow(results)) {
  latex_table <- paste0(
    latex_table,
    "\\textbf{", results$Variable[i], "}&",
    results$T_test[i], "&",
    results$Wilcoxon_test[i], " \\\\ \n"
  )
}
```

```r
latex_table <- paste0(
  latex_table,
  "\\bottomrule \\\\
\\end{tabular}
\\caption{T-test and Two-sample Wilcoxon rank-sum (Mann-Whitney) test results for the between-instructo
\\label{table:CountingDays_Between_Instructor_T_Test_Wilcoxon_Rank_Sum}
\\end{table*}"
)

# Output the LaTeX code
cat(latex_table)
```

```
## \begin{table*}[!htp]
## \centering
## \ra{1.6}
## \begin{tabular}{@{}p{3.7cm}p{3.1cm}p{4.3cm}@{}}
## \toprule
## & Two-sample t-test with equal variances & Two-sample Wilcoxon rank-sum (Mann--Whitney) test \\ \mid
## \textbf{Practiced Days}&5.616***&9.767*** \\
## \textbf{Practiced Questions}&1.544&2.745** \\
## \bottomrule \\
## \end{tabular}
## \caption{T-test and Two-sample Wilcoxon rank-sum (Mann-Whitney) test results for the between-instruct
## \label{table:CountingDays_Between_Instructor_T_Test_Wilcoxon_Rank_Sum}
## \end{table*}
```
